# Synthesis of Chiral Helic[1]triptycene[3]arenes and Their Enantioselective Recognition towards Chiral Guests Containing Aminoindan Groups

**DOI:** 10.3390/molecules26030536

**Published:** 2021-01-20

**Authors:** Jing Li, Ying Han, Chuan-Feng Chen

**Affiliations:** 1 Beijing National Laboratory for Molecular Sciences, CAS Key Laboratory of Molecular Recognition and Function, Institute of Chemistry, Chinese Academy of Sciences, Beijing 100190, China; lijing2015@iccas.ac.cn (J.L.); hanying463@iccas.ac.cn (Y.H.); 2 University of Chinese Academy of Sciences, Beijing 100049, China

**Keywords:** macrocyclic arene, enantioselective recognition, helic[1]triptycene[3]arene, ammonium salts

## Abstract

Starting from the enantiopure precursors, a pair of chiral macrocyclic arenes named helic[1]triptycene[3]arenes were conveniently synthesized. The circular dichroism (CD) spectra of the enantiomeric macrocyclic arenes exhibited mirror images, and the X-ray single crystal structures confirmed their absolute conformations as well. Moreover, the macrocyclic arenes showed strong complexation with secondary ammonium and primary ammonium salts containing aminoindan groups. In particular, the chiral macrocyclic arenes exhibited enantioselective recognition ability towards the chiral secondary ammonium salts containing aminoindan groups with an enantioselective ratio up to 3.89.

## 1. Introduction

Rasagiline is the second generation of monoamine oxidase inhibitor, which exhibits efficient capacity in blocking the breakdown of the neurotransmitter dopamine, and improving therapeutic effect on patients that suffered the decline in potency of long-term applications of dopamine [1]. Furthermore, the metabolite of rasagiline is an inactive non-benzedrinum with few side effects [2]. Azilect, namely rasagiline mesylate, is essentially a chiral secondary ammonium salt with excellent medicinal cure effects. However, there is little research into the recognition, and especially the enantioselective recognition, towards these bioactive guests based on synthetic receptors so far [3].

Macrocyclic arenes are a type of macrocycles which all composed of hydroxyl substituted aromatic rings bridged by methylene or methenyl groups [4]. Macrocyclic arenes have exhibited wide applications in host–guest chemistry [5,6,7,8,9,10], self-assembly [11,12], molecular machines [13], and material sciences [14,15,16]. Novel macrocyclic arenes have been reported successively in recent years to enrich the macrocyclic arene library [17]. Although the macrocyclic arene’s recognition ability towards ammonium salts have been well studied [18,19,20,21,22], their enantioselective recognition towards ammonium salts, and especially those bioactive ammonium salts, have been rarely reported [23].

**Figure 1:**
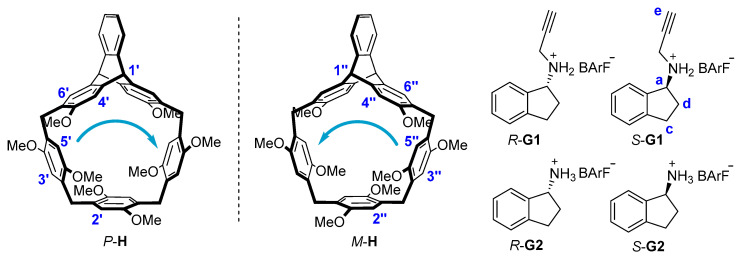
Structures and proton designations of the hosts (*P*-**H**/*M*-**H**) and the guests (*R*-**G1**/*S*-**G1** and *R*-**G2**/*S*-**G2**).

Previously, we [24] reported a new macrocyclic arene named helic[1]triptycene[3]arene that exhibited excellent recognition ability towards the organic ammonium salts. Since the protonated rasagiline and its metabolite were chiral ammonium salts, we deduced that enantiopure macrocyclic arenes based on the helic[1]triptycene[3]arene could show enantioselective recognition ability toward the chiral guests containing aminoindan groups. In this paper, we report a pair of enantiopure helic[1]triptycene[3]arenes *P*-**H** and *M*-**H**, which could be conveniently synthesized by one-pot reaction starting from the enantiopure precursors. *P*-**H** and *M*-**H** showed mirror CD spectra and they could also self-assemble into herringbone-like architecture in the solid states. The enantiopure macrocycles exhibited enantioselective recognition abilities towards chiral secondary ammonium salts (*R/S*-**G1**) and primary ammonium salts (*R/S***-G2**, Figure 1). In particular, the *P*-**H** showed high enantioselectivity towards *R*-**G1** than *S*-**G1** with the ratio up to 3.89:1.

## 2. Results and Discussion

### 2.1. Synthesis and Structures

**Scheme 1:**
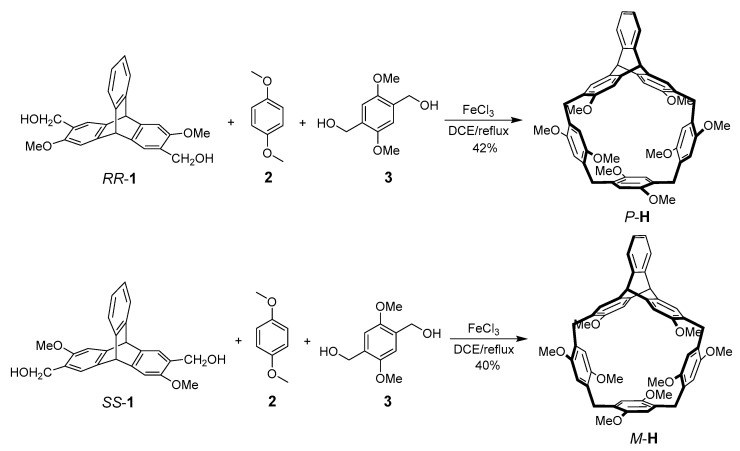
Synthetic Routes to *P*-**H** and *M*-**H**.

Synthesis of enantiomeric helic[1]triptycene[3]arenes *P*-**H**/*M*-**H** was depicted in Scheme 1. *RR*-**1** and *SS*-**1** were first obtained by efficient chiral resolution of the racemic triptycene derivative with HPLC on chiral column (Figures S1–S3, in the Supplementary Materials). By the reaction of *RR*-**1**, 1,4-dimethoxybenzene **2** and 1,4-dihydroxymethyl-2,5-dimethoxybenzene **3** [25] with a catalytic amount of FeCl_3_ in dichloroethane, we conveniently obtained *P*-**H** with 42% yield. Similarly, starting from *SS*-**1** and **2** and **3**, we obtained *M*-**H** with 40% yield. *P*-**H** and *M*-**H** both exhibited good solubility in common solvents including chloroform, dichloromethane, dichloroethane and tetrahydrofuran. Starting from commercial materials, the guests were conveniently obtained by two-step reactions as well (see Materials and Methods for details).

**Figure 2:**
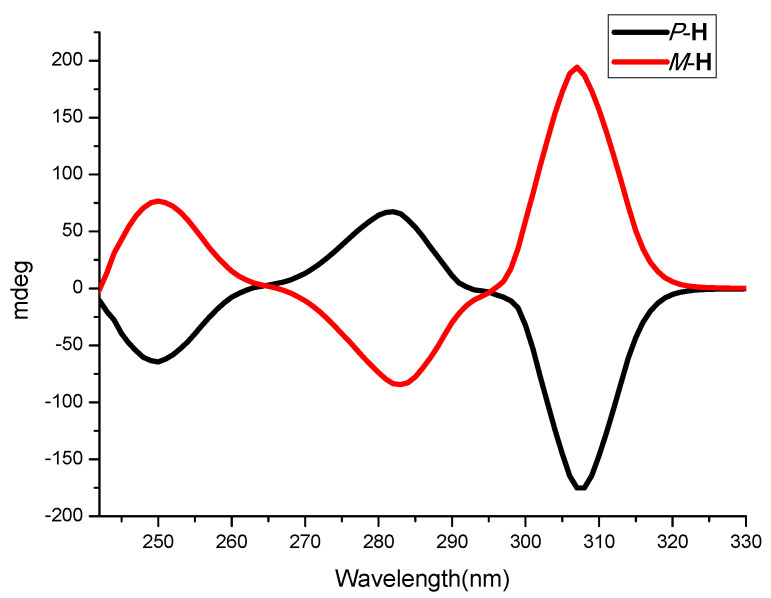
CD spectra of *P*-**H** and *M*-**H** (CH_2_Cl_2_, *c* = 2×10^−5^ *M^−1^*, T = 298 K).

The specific rotation [α]_D_^25^ of *RR*-**1** in dichloromethane was 21°. By cyclization reaction of *RR*-**1** with **2** and **3**, the resulting product *P*-**H** showed its [α]_D_^25^ of 123° in dichloromethane, which was significantly bigger than that of *RR*-**1**. Similarly, the specific rotation [α]_D_^25^ of *SS*-**1** in dichloromethane was −21°, while [α]_D_^25^ of the macrocyclic arene *M*-**H** in dichloromethane was −128°. Moreover, the circular dichroism (CD) spectra of *P*-**H** and *M*-**H** showed mirror images (Figure 2), which confirmed their enantiomeric structures. The enantiopure hosts and guests were further characterized by their ^1^H NMR, ^13^C NMR and HRMS spectra.

### 2.2. Crystal Structures

**Figure 3:**
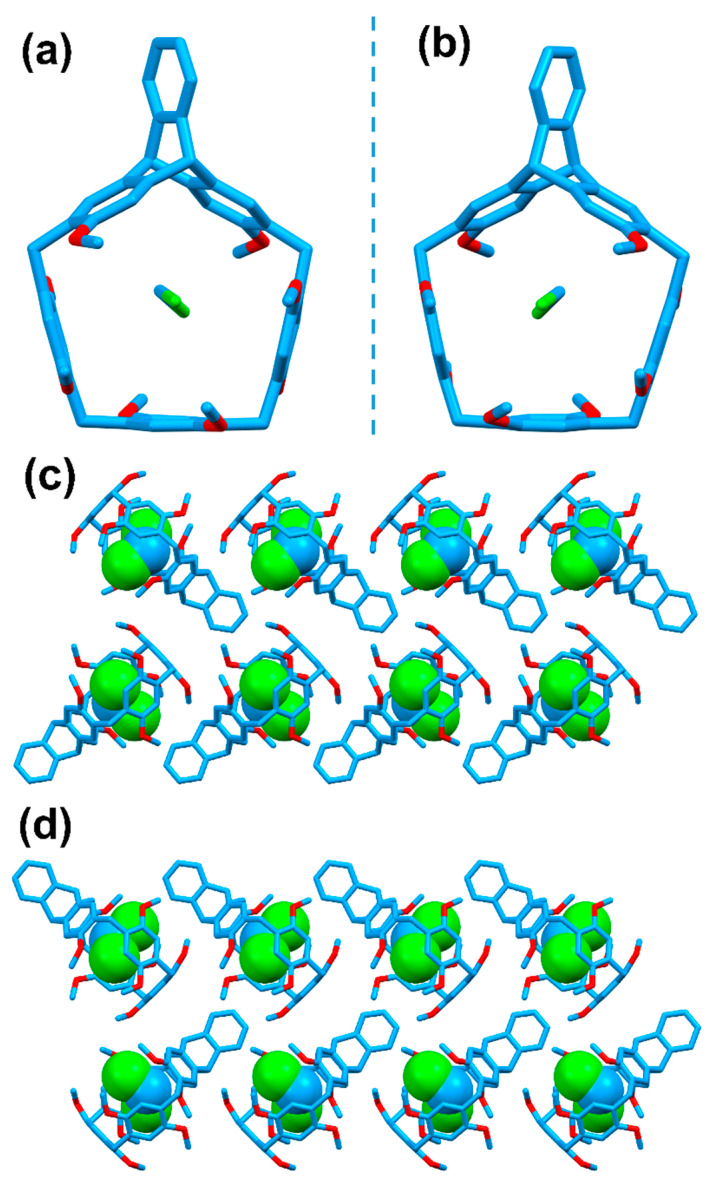
Crystal structures of (**a**) *P*-**H**, (**b**) *M*-**H** and packing mode of (**c**) *P*-**H**, (**d**) *M*-**H** along the *c* axis. Hydrogen atoms were omitted for clarity.

By slow evaporation of the macrocyclic hosts in mixture solution of dichloromethane and isopropyl ether at room temperature, we obtained the colorless single crystals. As shown in Figure 3, the macrocycles adopted the bucket-like structures with the mirror image correspondence. For *P*-**H**, the methoxy groups on one rim adopted the clockwise orientation, while for *M*-**H**, the methoxy groups on one rim adopted the anticlockwise orientation. This result was in accordance with their mirror images reflected in CD spectra. Moreover, one dichloromethane molecule was encapsulated in the center of the macrocyclic cavity. It was further found that both *P*-**H** and *M*-**H** could self-assemble into herringbone-like architectures along *c* axis while the orientations were different from each other (Figure 3c,d).

**Figure 4:**
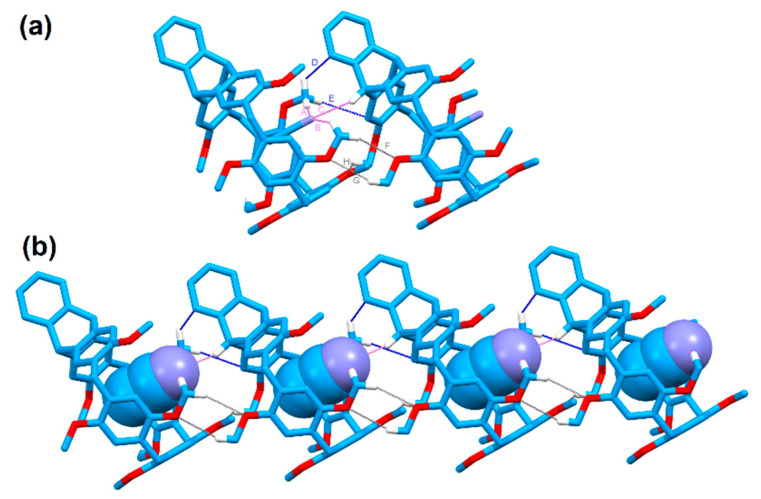
(**a**) Crystal structure and (**b**) packing mode along the *c* axis of *P*-**H**. Dotted lines denoted the noncovalent interactions between *P*-**H** and CH_3_CN (violet for C−H···N interactions, grey for C−H···O interactions and blue for C−H···π interactions). Hydrogen atoms not involved in the noncovalent interactions were omitted for clarity.

We also obtained the colorless single crystal of *P*-**H** by slow evaporation of acetonitrile and isopropyl ether solution at room temperature. As shown in Figure 4, one acetonitrile molecule was encapsulated in the center of the *P*-**H**’s cavity. There existed multiple C−H···N interactions between acetonitrile and the two adjacent macrocycles in the distance of 2.662 Å for A, 2.736 Å for B and 2.738 Å for C, respectively. Since the encapsulated acetonitrile showed noncovalent interactions with the adjacent macrocycle, the two adjacent macrocycles were thus drawing closer compared with the macrocycle that encapsulated dichloromethane. There existed multiple C−H···π interactions (2.871 Å for D and 2.858 Å for E) and multiple C−H···O interactions (2.579 Å for F, 2.667 Å for G and 2.656 Å for H) between the two adjacent macrocycles. Moreover, the macrocycles could pack in a herringbone pattern along the *c* axis in a more closely packed mode compared with the aforementioned macrocycle that encapsulated dichloromethane.

### 2.3. Enantioselective Recognition Ability of the Macrocyclic Hosts towards Ammonium Salts

To investigate the enantioselective complexation of enantiomeric helic[1]triptycene[3]arenes *P*-**H**/*M*-**H,** two pairs of enantiopure bioactive ammonium salts containing aminoindan groups have been chosen as the guests. *R*-**G1** is the protonated derivative of rasagiline, which is an important monoamine oxidase inhibitor [1]. *R*-**G2** is the protonated derivative of the metabolite of rasagiline, which is an inactive non-benzedrinum with few side effects [2].

**Figure 5:**
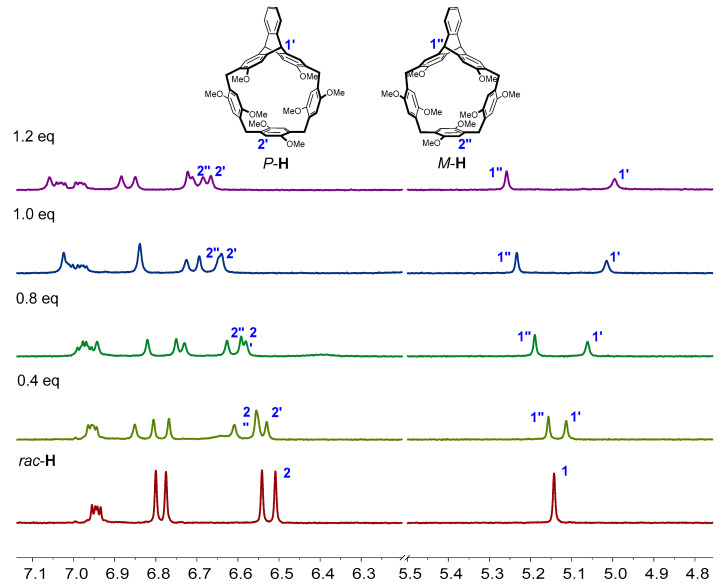
Partial ^1^H NMR spectra (400MHz, 298 K, CDCl_3_) of 2.0 mM *rac*-**H** with different amounts of *R*-**G1**.

Firstly, we tested the enantioselective recognition ability towards the guests by the racemic host through ^1^H NMR spectra. When *R*-**G1** was added into *rac*-**H** in CDCl_3_ solution, a new set of proton signals which were different from those of the free host and the free guest appeared, indicating the host-guest complexation was the fast exchange process in NMR timescale (Figure 5). When *R*-**G1** was added into the host’s solution, the proton signal of H_1_ of *rac*-**H** split. With the increasing amount of *R*-**G1** into the host’s solution, the splitting trend was even greater. The proton signal of H_2_ of *rac*-**H** also split after the addition of *R*-**G1** into the host’s solution. These phenomena indicated that helic[1]triptycene[3]arene showed enantioselective recognition ability towards **G1**. Similarly, upon addition of *R*-**G2** into *rac*-**H** in CD_2_Cl_2_ solution, the proton signals of *rac*-**H** also split (Figure S20, in the Supplementary Materials), which confirmed the chiral recognition ability of the macrocycle towards **G2**.

**Figure 6:**
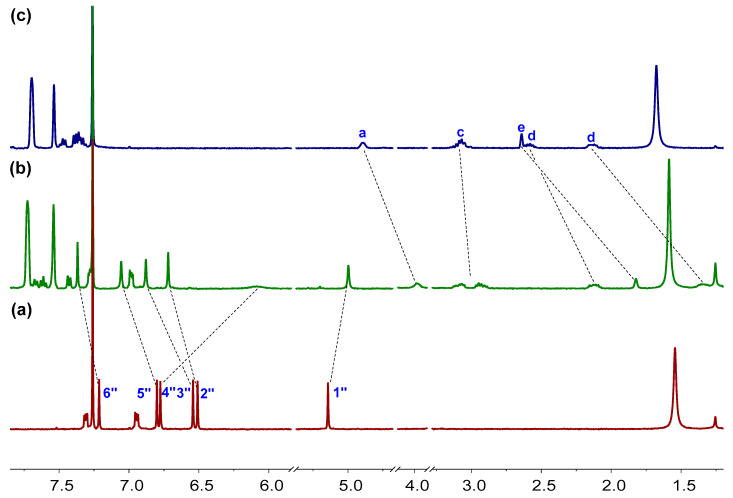
Partial ^1^H NMR spectra (500 MHz, 298 K, CDCl_3_) of (**a**) *M*-**H**, (**b**) *M*-**H** + 1.0 equiv. *S*-**G1**, (**c**) *S*-**G1**. [*M*-**H**]_0_ = 3.0 mM.

The host–guest complexations between *P*-**H**/*M*-**H** and the chiral guests were then investigated by ^1^H NMR spectroscopy. When equimolar *M*-**H** and *S*-**G1** were mixed in CDCl_3_ solution, a new set of proton signals different from free host and free guest appeared, suggesting the complexation was fast exchange process in NMR timescale (Figure 6). The proton signals of H_1_^″^ and H_4_^″^ of *M*-**H** showed upfield shifts while other proton signals of *M*-**H** showed downfield shifts. The proton signals of H_a_–H_e_ of *S*-**G1** all showed upfield shifts probably due to the shielding effect of *M*-**H**. Other host–guest complexes also showed similar complexation mode and the complexations were all fast exchange processes in NMR timescale (Figures S21–S28, in the Supplementary Materials). Besides, the CD spectra of the enantiomeric hosts in the presence of the guests exhibited mirror images as well (Figure S72, in the Supplementary Materials). Compared with the individual hosts, the CD spectra of the host-guest complexes exhibited slight enhanced cotton effects at 248, 288, and 308 nm.

In order to quantitively study the complexation strength between the hosts and the guests, ^1^H NMR titration experiments were performed, and the association constants were calculated by the non-linear fitting method [26]. The complex-ratios were all calculated to be 1:1 through Job plot method (Figures S37, S40, S43, S46, S49, S52, S55 and S58, in the Supplementary Materials), and the association constants were summarized in Table 1. The association constants between the hosts and the secondary ammonium salts were in the vicinity of 10^3^ M^−1^ in chloroform. *P*-**H** showed biggest association constant with *R*-**G1** in the value of (7.63 ± 0.75) × 10^3^ M^−1^. Meanwhile, *P*-**H** showed moderate association constant with *S*-**G1** in the value of (1.96 ± 0.18) × 10^3^ M^−1^. It’s noteworthy that the association constant of *P*-**H** towards *R*-**G1** was 3.89 times of *P*-**H** towards *S*-**G1**. The association constants between the hosts and the primary ammonium salts were in the vicinity of 10^2^ M^−1^ in dichloromethane. And the association constant of *P*-**H** towards *R*-**G2** was 1.61 times of *P*-**H** towards *S*-**G2**.

HRMS analysis also confirmed the 1:1 complexation ratio of those host-guest complexes in gas phase. The strongest peak at *m/z* 948.4447 for [*P*-**H** *R*-**G1**-BArF]^+^ was found by using a solution of *P*-**H** and *R*-**G1** in chloroform, which was in accordance with formation of the 1:1 complex in solution. Similarly, the strongest peaks at *m/z* 948.4444, 948.4446, 948.4442, 910.4316, 910.4319, 910.4323, and 910.4319 for [*P*-**H**·*S*-**G1**-BArF]^+^, [*M*-**H**·*R*-**G1**-BArF]^+^, [*M*-**H**·*S*-**G1**-BArF]^+^, [*P*-**H**·*R*-**G2**-BArF]^+^, [*P*-**H**·*S*-**G2**-BArF]^+^, [*M*-**H**·*R*-**G2**-BArF]^+^, [*M*-**H**·*S*-**G2**-BArF]^+^ were also observed, respectively (Figures S29–S36, in the Supplementary Materials), which further verified formation of the 1:1 complexes.

### 2.4. DFT and IGM Analyses of Complexes P-**H**·R-**G1** and P-**H**·S-**G1**

**Figure 7:**
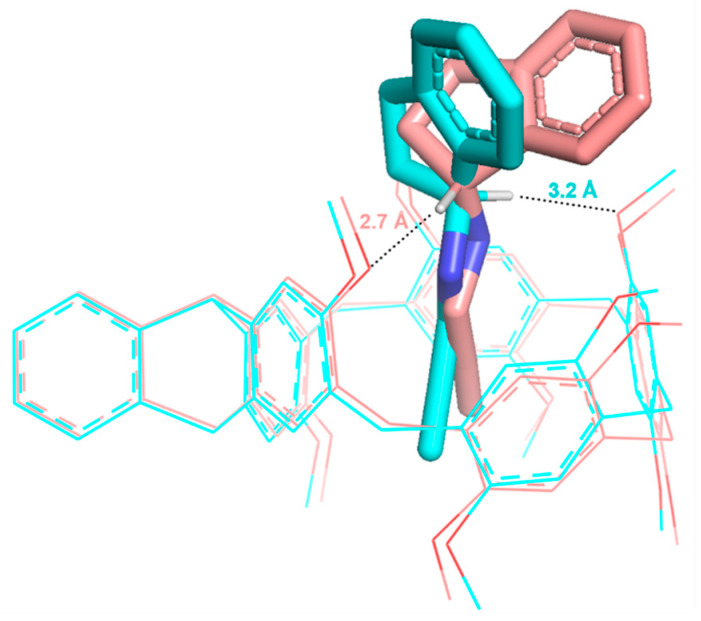
Superimposed views of optimized structures for the complexes between host *P*-**H** and the enantiomers *R*-**G1**/*S*-**G1**. Pink and light blue denote guests *R*-**G1** and *S*-**G1**, respectively. Dotted lines denote the C-H···O hydrogen bonds in the host-guest complexes.

To further investigate the enantioselective recognition of the chiral macrocycles towards *R*-**G1** and *S*-**G1**, DFT calculations for the host-guest complexes without consideration of the counter anions were carried out by using B3LYP/6-31G(d) method [27]. As shown in Figure 7, the alkynyl group and ammonium group of the guests were both located inside the cavity of *P*-**H**, while the indane group was outside the cavity. According to the superimposed views of optimized structures for the complexes *P*-**H**·*R*-**G1** and *P*-**H**·*S*-**G1**, the main difference for the complexes lay in the noncovalent interactions between the chiral center of the guests and the methoxy group at the rim of the host. For complex *P*-**H**·*R*-**G1**, the C-H···O hydrogen bonds between the hydrogen of the chiral carbon of *R*-**G1** and the oxygen at the rim of the triptycene unit was in the distance of 2.7 Å. For complex *P*-**H**·*S*-**G1**, the C-H···O hydrogen bonds between the hydrogen of the chiral carbon of *S*-**G1** and the oxygen at the rim of the phenyl unit was in the distance of 3.2 Å, which was essentially weak noncovalent interactions compared with *P*-**H**·*R*-**G1** [28]. Moreover, complex *P*-**H**·*R*-**G1** showed lower energy (−13.0 kcal mol^−1^) than that of *P*-**H**·*S*-**G1** (−12.1 kcal mol^−1^), which indicated that complex *P*-**H**·*R*-**G1** was thermodynamically stable adduct than complex *P*-**H**·*R*-**G1** (Table S8, in the Supplementary Materials). This was consistent with the result of *P*-**H**’s preference for *R*-**G1** over *S*-**G1**.

**Figure 8:**
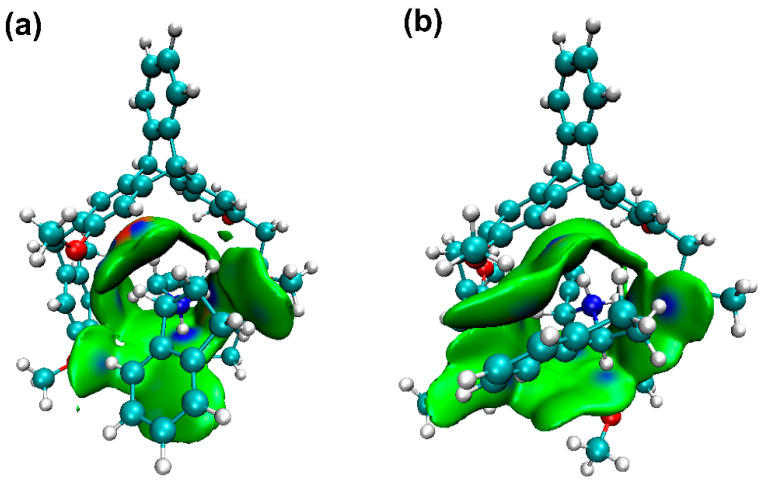
IGM analyses of the complexes (**a**) *P*-**H**·*R*-**G1**, (**b**) *P*-**H**·*S*-**G1** (*δ*ginter = 0.002).

Moreover, IGM [29] analyses for complexes *P*-**H**·*R*-**G1** and *P*-**H**·*S*-**G1** were also carried out based on their optimal structures. As shown in Figure 8, there existed not only the dispersion force (the green region) but also multiple C−H···π interactions and N−H···O and C−H···O hydrogen binding interactions (the blue region) between *P*-**H** and *R*-**G1**, thus generating the strong interactions in this host-guest complex. For *P*-**H**·*S*-**G1**, there existed weak noncovalent intermolecular interactions in the host-guest complex, which might result in the lower association constant for *P*-**H**·*S*-**G1** compared with that of *P*-**H**·*R*-**G1**.

## 3. Conclusions

In summary, we have conveniently synthesized a pair of enantiopure helic[1]triptycene[3]arenes by one-pot reaction. The CD spectra of the chiral macrocycles showed mirror images, and X-ray crystal structures showed that one dichloromethane or acetonitrile molecule could be encapsulated in the cavities of the macrocycles, while the macrocyclic molecules could pack into the herringbone-like architectures along the *c* axis. Moreover, the macrocycles exhibited strong complexation with the bioactive guests containing aminoindan groups. It was further found that the chiral macrocyclic hosts showed enantioselective recognition ability towards chiral guests **G1** and **G2**. In particular, *P*-**H** showed preference for *R*-**G1** over *S*-**G1** with an enantioselective ratio up to 3.89. The selectivity might originate from the noncovalent interactions between the chiral carbon center of the guests and the methoxy groups at the rim of the hosts.

## 4. Materials and Methods

### 4.1. General Methods

All the reagents were commercially available and used without further purification. Reactions were carried out under inert and anhydrous conditions unless otherwise noted. Anhydrous solvents were dried from 4 Å molecular sieves. Flash column chromatography was performed on 200–300 mesh silica gel. ^1^H and ^13^C NMR spectra were measured at 298 K. The ionization methods used in mass spectrometry were atmospheric pressure chemical ionization (APCI) and electrospray ionization (ESI). Melting points, taken on an electrothermal melting point apparatus, were uncorrected. X-Ray single crystal data were measured by XtaLAB Synergy-R at 170 K. Circular dichroism spectra were measured by J-810 at 298 K.

### 4.2. General Synthesis Procedure for the Hosts

To a solution of *RR*-**1** (1.00 g, 2.65 mmol), **2** (0.75 g, 5.30 mmol), and **3** (0.55 g, 2.65 mmol) in dichloroethane (400 mL) was added FeCl_3_ (0.05 g, 0.25 mmol). Then the mixture was refluxed in the oil bath under argon atmosphere for 12 h. After cooling to room temperature, the mixture was evaporated under vacuum and the residue was further purified by flash column chromatography on silica gel (eluent: petroleum ether/ethyl acetate = 4:1, *R_f_* = 0.4) to afford *P*-**H** as white solid (0.85 g, 42%).

According to the above method, *M*-**H** was obtained as white solid in 40% yield.

### 4.3. General Synthesis Procedure for the Guests

To the solution of (*R*)-*N*-(2-Propynyl)-2,3-dihydroinden-1-amine (171.2 mg, 1.0 mmol) in DCM (5 mL) was added excessive hydrochloric acid. The mixture was stirred for 30 min under room temperature and then filtrated to collect the residue. Then the residue was dissolved in DCM/H_2_O (1:1, *v/v*, 10 mL) and sodium tetrakis [3,5-bis(trifluoromethyl)phenyl]borate (NaBArF) (886.2 mg, 1.0 mmol) was added subsequently. The mixture was stirred for 2 h under room temperature and then extracted with CH_2_Cl_2_. The organic phase was dried with MgSO_4_, and concentrated to give *R*-**G1** (994.1 mg, 96%) as the white solid.

According to the similar method, *S*-**G1** was prepared as white solid in 97% yield, *R*-**G2** was prepared as white solid in 98% yield, and *S*-**G2** was prepared as white solid in 96% yield.

### 4.4. Characterization Data

*RR*-**1**.

^1^H NMR (300 MHz, CDCl_3_): *δ* 7.35–7.32 (m, 2H), 7.29 (s, 2H), 6.99 (s, 2H), 6.98-6.93 (m, 2H), 4.56 (s, 4H), 3.81 (s, 6H), 2.07 (s, 2H). ^13^C NMR (75 MHz, CDCl_3_): *δ* 155.0, 147.0, 145.4, 137.4, 125.2, 124.9, 124.1, 123.3, 107.1, 61.9, 55.6, 53.6. M.p.: 156.7–159.8 °C. [α]_D_^25^ = +21° (*c* = 0.25, CH_2_Cl_2_). HRMS (APCI) *m/z*: [M-H]^+^ calcd for C_24_H_21_O_4_^−^ 373.1434; found 373.1429.

SS-**1**.

^1^H NMR (400 MHz, CDCl_3_): *δ* 7.33 (dd, J = 5.3, 3.2 Hz, 2H), 7.29 (s, 2H), 7.00–6.97 (m, 2H), 6.97–6.92 (m, 2H), 4.55 (s, 4H), 3.78 (s, 6H), 2.10 (s, 2H). ^13^C NMR (100 MHz, CDCl_3_): *δ* 154.9, 147.0, 145.5, 137.5, 125.2, 125.0, 124.1, 123.3, 107.1, 61.8, 55.6, 53.6. M.p.: 159.2-161.6 °C. [α]_D_^25^ = −21° (*c* = 0.25, CH_2_Cl_2_). HRMS (APCI) *m/z*: [M-H]^+^ calcd for C_24_H_21_O_4_^−^ 373.1434; found 373.1428.

*P*-**H**.

^1^H NMR (400 MHz, CDCl_3_): *δ* 7.32–7.30 (m, 2H), 7.21 (s, 2H), 6.96-6.93 (m, 2H), 6.80 (s, 2H), 6.78 (s, 2H), 6.54 (s, 2H), 6.51 (s, 2H), 5.14 (s, 2H), 3.81 (d, *J* = 12.9 Hz, 2H), 3.75 (s, 4H), 3.69 (s, 6H), 3.64 (d, *J* = 12.9 Hz, 2H), 3.51 (s, 6H), 3.48 (s, 6H), 3.39 (s, 6H). ^13^C NMR (125 MHz, CDCl_3_): *δ* 154.0, 151.0, 150.9, 145.7, 145.2, 137.3, 129.0, 128.8, 128.5, 126.3, 125.9, 124.8, 123.1, 114.5, 114.5, 114.1, 106.9, 56.1, 56.0, 55.9, 55.3, 53.4, 29.9, 29.6. M.p.: 221.8–223.9 °C. [α]_D_^25^ = + 123° (*c* = 0.25, CH_2_Cl_2_). HRMS (ESI) *m/z*: [M+Na]^+^ calcd for C_50_H_48_O_8_Na^+^ 799.3241; found 799.3252.

*M*-**H**.

^1^H NMR (500 MHz, CDCl_3_): *δ* 7.32–7.30 (m, 2H), 7.21 (s, 2H), 6.95–6.94 (m, 2H), 6.80 (s, 2H), 6.77 (s, 2H), 6.54 (s, 2H), 6.51 (s, 2H), 5.14 (s, 2H), 3.81 (d, *J* = 12.9 Hz, 2H), 3.76 (s, 4H), 3.68 (s, 6H), 3.64 (d, *J* = 13.0 Hz, 2H), 3.51 (s, 6H), 3.47 (s, 6H), 3.39 (s, 6H). ^13^C NMR (125 MHz, CDCl_3_): *δ* 154.0, 151.0, 150.9, 145.7, 145.2, 137.3, 129.0, 128.8, 128.5, 126.3, 125.9, 124.8, 123.1, 114.5, 114.5, 114.1, 106.9, 56.1, 56.0, 55.9, 55.3, 53.4, 29.9, 29.6. M.p.: 219.8-221.2 °C. [α]_D_^25^ = −128° (*c* = 0.25, CH_2_Cl_2_). HRMS (ESI) *m/z*: [M+Na]^+^ calcd for C_50_H_48_O_8_Na^+^ 799.3241; found 799.3221.

*R*-**G1**.

^1^H NMR (400 MHz, CDCl_3_): *δ* 7.72 (s, 8H), 7.56 (s, 4H), 7.52–7.48 (m, 1H), 7.44–7.30 (m, 3H), 7.02 (s, 2H), 4.93 (d, *J* = 6.7 Hz, 1H), 3.79–3.78 (m, 2H), 3.11-3.07 (m, 2H), 2.67 (t, *J* = 2.5 Hz, 1H), 2.65-2.55 (m, 1H), 2.16–2.12 (m, 1H). ^13^C{^1^H} NMR (100 MHz, CDCl_3_): *δ* 161.7 (q, ^1^J_CB_ = 50 Hz), 144.4, 134.8, 133.3, 132.1, 129.0 (q, ^2^J_CF_ = 32 Hz), 128.3, 126.5, 124.9, 124.6 (q, ^1^J_CF_ = 271 Hz), 117.6, 80.3, 70.5, 64.3, 35.7, 29.6, 29.2. M.p.: 81.8–82.9 °C. [α]_D_^25^ = +4° (*c* = 0.25, CH_2_Cl_2_). HRMS (ESI) *m/z*: [M-BArF]^+^ calcd for C_12_H_14_N^+^ 172.1121; found 172.1118.

*S*-**G1**.

^1^H NMR (400 MHz, CDCl_3_): *δ* 7.72 (s, 8H), 7.56 (s, 4H), 7.49–7.42 (m, 1H), 7.38 (d, *J* = 8.5 Hz, 2H), 7.32 (t, *J* = 7.5 Hz, 1H), 4.92 (dd, *J* = 7.5, 2.4 Hz, 1H), 3.80 (d, *J* = 2.6 Hz, 2H), 3.15-3.00 (m, 2H), 2.63 (t, *J* = 2.8 Hz, 1H), 2.59-2.56 (m, 1H), 2.19–2.12 (m, 1H). ^13^C{^1^H} NMR (100 MHz, CDCl_3_): *δ* 161.7 (q, ^1^J_CB_ = 50 Hz), 144.4, 134.8, 133.3, 132.0, 128.9 (q, ^2^J_CF_ = 31 Hz), 128.3, 126.5, 124.8, 124.6 (q, ^1^J_CF_ = 271 Hz), 117.6, 80.3, 70.6, 64.2, 35.7, 29.6, 29.2. M.p.: 78.9–80.7 °C. [α]_D_^25^ = −3° (*c* = 0.25, CH_2_Cl_2_). HRMS (ESI) *m/z*: [M-BArF]^+^ calcd for C_12_H_14_N^+^ 172.1121; found 172.1119.

*R*-**G2**.

^1^H NMR (500 MHz, CD_2_Cl_2_): *δ* 7.74 (s, 8H), 7.58 (s, 4H), 7.48 (t, *J* = 7.5 Hz, 1H), 7.42 (d, *J* = 7.5 Hz, 2H), 7.37 (t, *J* = 7.5 Hz, 1H), 6.31-5.98 (m, 3H), 5.20–5.17 (m, 1H), 3.26-3.10 (m, 2H), 2.80–2.75 (m, 1H), 2.26-2.18 (m, 1H). ^13^C{^1^H} NMR: (125 MHz, CD_2_Cl_2_) *δ* 163.1 (q, ^1^J_CB_ = 50 Hz), 145.6, 136.2, 135.6, 133.4, 130.2 (q, ^2^J_CF_ = 31 Hz), 129.8, 127.8, 126.0 (q, ^1^J_CF_ = 270 Hz), 125.4, 118.9, 62.1, 32.1, 31.0. M.p.: 66.5–67.5 °C. [α]_D_^25^ = + 3° (*c* = 0.25, CH_2_Cl_2_). HRMS (ESI) *m/z*: [M-BArF]^+^ calcd for C_9_H_12_N^+^ 134.0964; found 134.0965.

*S*-**G2**.

^1^H NMR (500 MHz, CD_2_Cl_2_): *δ* 7.74 (s, 8H), 7.58 (s, 4H), 7.48 (t, *J* = 7.2 Hz, 1H), 7.41 (d, *J* = 8.3 Hz, 2H), 7.37 (t, *J* = 7.4 Hz, 1H), 5.68 (s, 3H), 5.17 (d, *J* = 7.3 Hz, 1H), 3.27–3.08 (m, 2H), 2.86–2.69 (m, 1H), 2.27–2.14 (m, 1H). ^13^C{^1^H} NMR (125 MHz, CD_2_Cl_2_): *δ* 163.1 (q, ^1^J_CB_ = 50 Hz), 145.6, 136.2, 135.8, 133.4, 130.2 (q, ^2^J_CF_ = 31 Hz), 129.8, 127.8, 126.0 (q, ^1^J_CF_ = 270 Hz), 125.4, 118.9, 62.2, 32.2, 31.0. M.p.: 68.1–69.4 °C. [α]_D_^25^ = − 3° (*c* = 0.25, CH_2_Cl_2_). HRMS (ESI) *m/z*: [M-BArF]^+^ calcd for C_9_H_12_N^+^ 134.0964; found 134.0963.

## Figures and Tables

**Table 1:** Association Constants (*K_a_*/M^−1^) for 1:1 Complexes Between Chiral **H** and Chiral **G1**–**G2** at 298 K.

	*P*-H		*M*-H	
Guests	*K_a_* M^−1^	*K_R_/K_S_*	*K_a_* M^−1^	*K_R_/K_S_*
*R*-G1 ^1^	(7.63 ± 0.75) × 10^3^	3.89/1	(2.10 ± 0.20) ×10^3^	1/3.28
*S*-G1 ^1^	(1.96 ± 0.18) × 10^3^		(6.98 ± 0.67) ×10^3^	
*R*-G2 ^2^	(3.44 ± 0.32) ×10^2^	1.61/1	(2.06 ± 0.19) ×10^2^	1/1.40
*S*-G2 ^2^	(2.14 ± 0.19) × 10^2^		(2.88 ± 0.27) ×10^2^	

^1^ In CDCl_3_. ^2^ In CD_2_Cl_2_.

## Data Availability

The data presented in this study are openly available in the article and Supplementary Materials.

## Supplementary Materials

The following are available online: HPLC charts, NMR spectra of new compounds, high resolution mass spectra for the complexes, determination of association constants for the complexes, CD spectra of the complexes, crystal structures and DFT calculations.
