# Supplementary material for: Synthesis of Chiral Helic[1]triptycene[3]arenes and Their Enantioselective Recognition towards Chiral Guests Containing Aminoindan Groups
Source: Molecules. 2021 Jan 20;26(3):536. doi: 10.3390/molecules26030536 (PMC7864338; doi:10.3390/molecules26030536)
Supplement: Supplementary file 1 [file molecules-26-00536-s001.pdf]

Electronic Supplementary Information

Synthesis of Chiral Helic[1]triptycene[3]arenes and Their Enantioselective Recognition Towards Chiral Guests Containing Aminoindan Groups

Jing Li,[1,2] Ying Han, [1] and Chuan-Feng Chen[1,2,*]

[a]*Beijing National Laboratory for Molecular Sciences, CAS Key Laboratory of Molecular Recognition and Function, Institute of Chemistry, Chinese Academy of Sciences, Beijing 100190, China.*

[b]*University of Chinese Academy of Sciences, Beijing 100049, China.*

E-mail: cchen@iccas.ac.cn

Table of contents

1. HPLC Charts……….........................…...……...…………………….………S3

2. NMR Spectra of New Compounds…….……………………………….….………….S5

3. ^{1}H NMR Studies on the Complexation Between **H** and **G1-G2**……...................S13

4. High Resolution Mass Spectra for the Complexes……..………………………..….S18

5. Determination of Association Constants for the Complexes................................S22

6. Crystal Structures……………..……...……………..….………..……….…..S34

7. DFT Calculations for Complexation Between **H** and **G1-G2** ……………….….S39

8. CD Spectra of Chiral Hosts and the Host-Guest Complexes………...………….….S68

9. ^{1}H NMR and 2D NMR Spectra for the Complexes………………………….….S69

1. HPLC Charts

Optical Resolution Conditions

Column: Chiralpak® IE 50 mm × 250nm

Mobile Phase: MeOH

Flow Rate: 1 mL/min

Wave Length UV 214nm

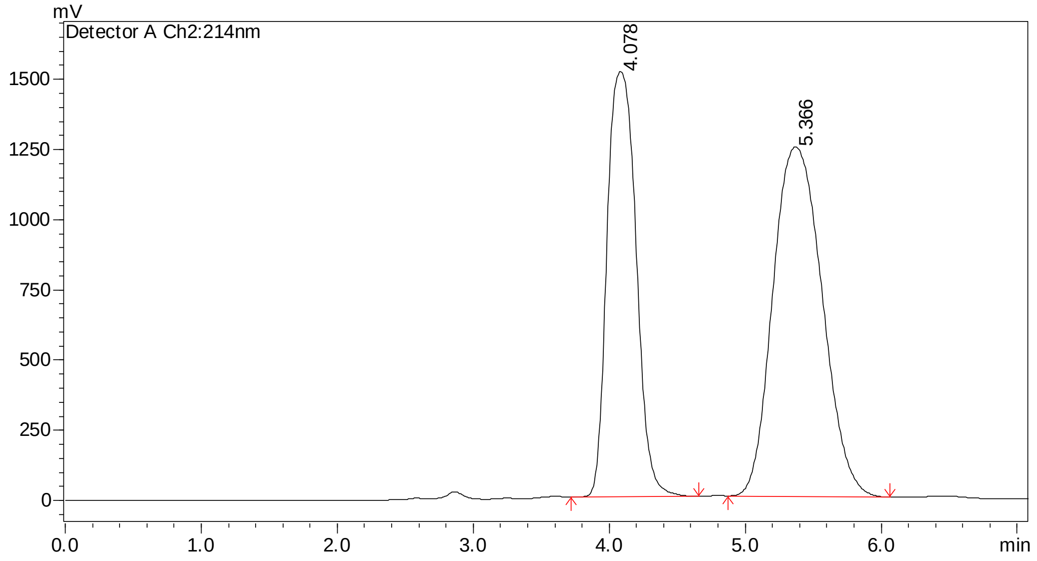

Figure S1. HPLC profile of *rac*-**1.**

Table S1. The summary of HPLC profiles of *rac*-**1.**

Compound	Peak	Ret. Time	Area	Area%
RR-**1**	1	4.078	22502285	41.7324
SS-**1**	2	5.366	31418174	58.2676

Analysis of the Enantiomer Excess of *RR*-1 and *SS*-1

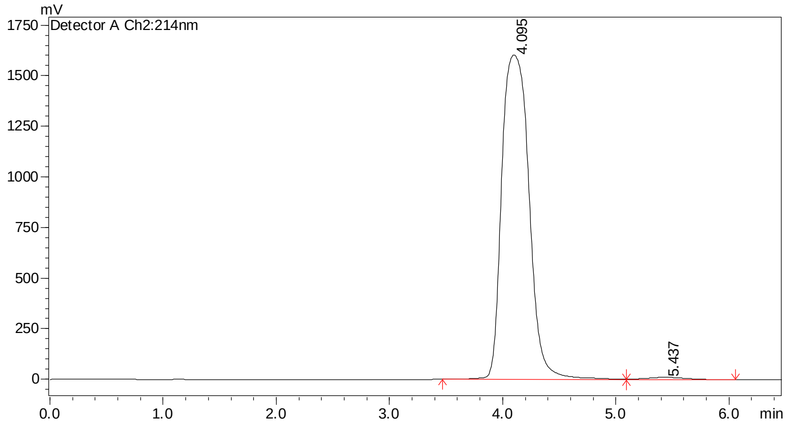

Figure S2. HPLC profile of *RR*-**1.**

Table S2. The summary of HPLC profiles of *RR*-**1.**

Compound	Peak	Ret. Time	Area	Area%	ee value
RR-**1**	1	4.093	3371935	99.5847	>99%
SS-**1**	2	5.439	14063	0.4153	

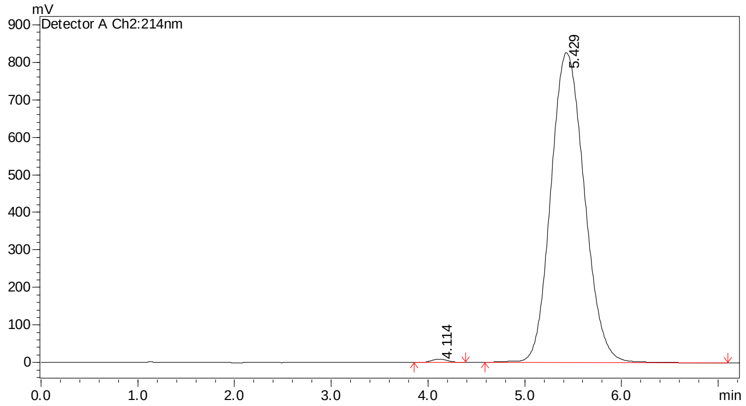

Figure S3. HPLC profile of *SS*-**1.**

Table S3. The summary of HPLC profiles of *SS*-**1.**

Compound	Peak	Ret. Time	Area	Area%	ee value
RR-**1**	1	4.114	100132	0.4982	
SS-**1**	2	5.429	19998978	99.5018	>99%

2. NMR Spectra of New Compounds

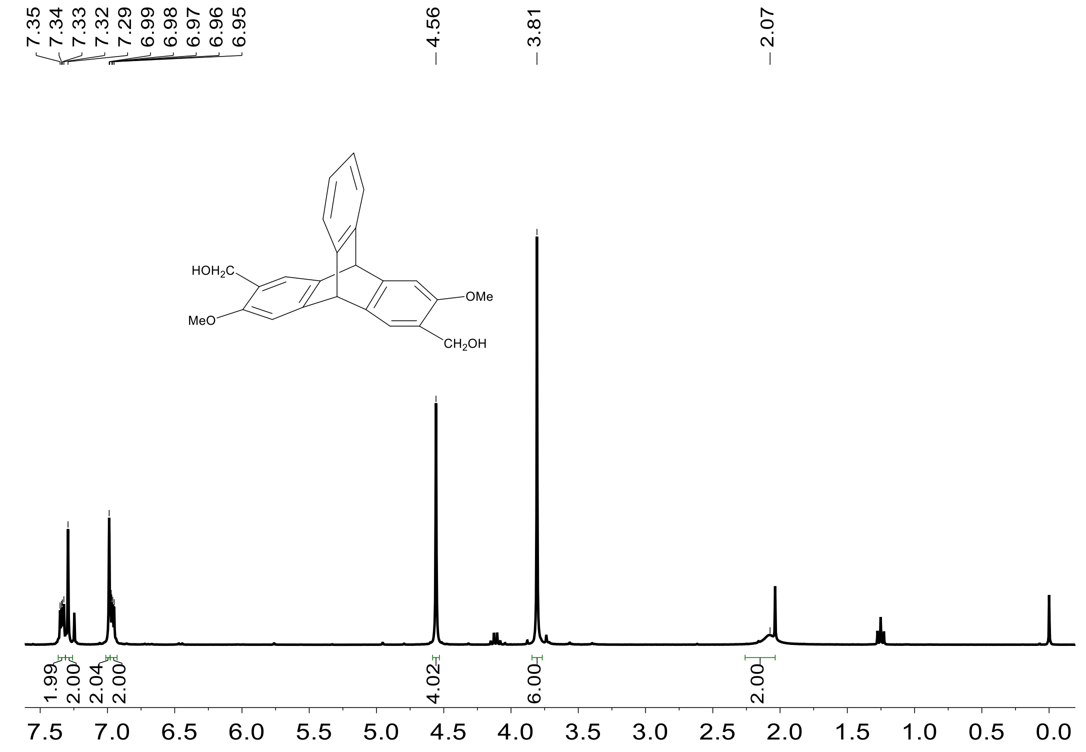

Figure S4. ^{1}H NMR spectrum (300 MHz, $CDCl_3$, 298 K) of *RR*-**1**.

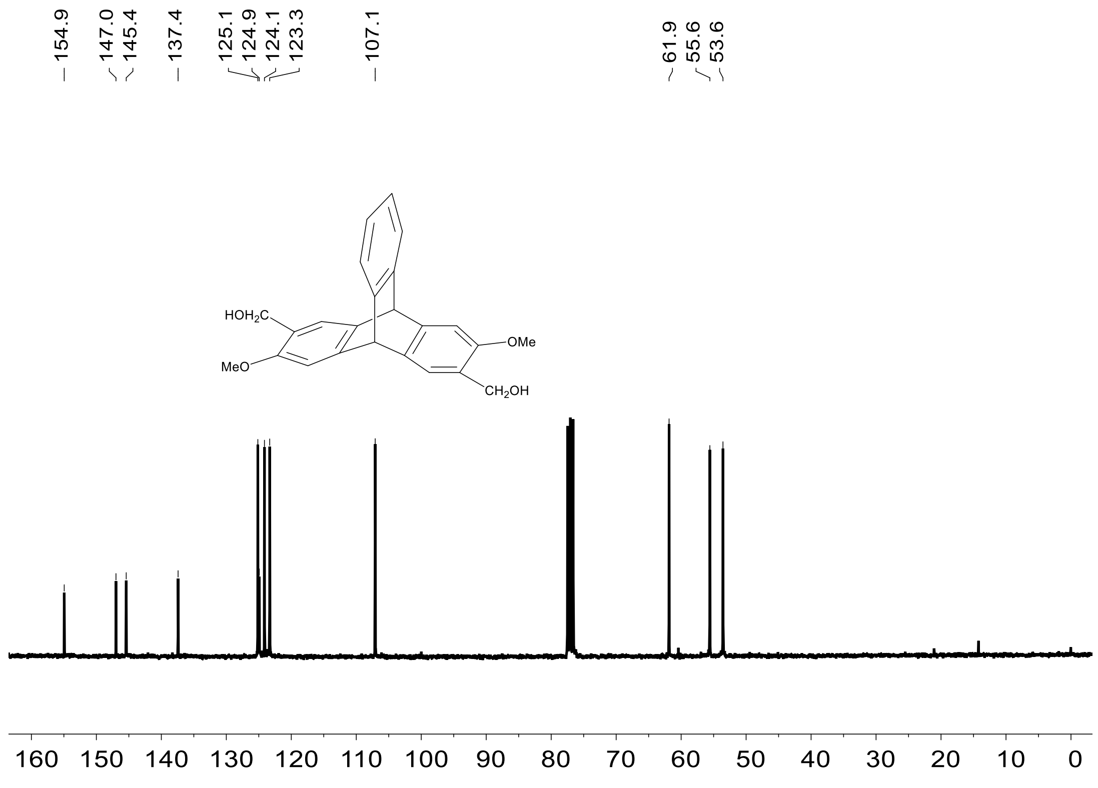

Figure S5. ^{13}C NMR spectrum (75 MHz, $CDCl_3$, 298 K) of *RR*-**1**.

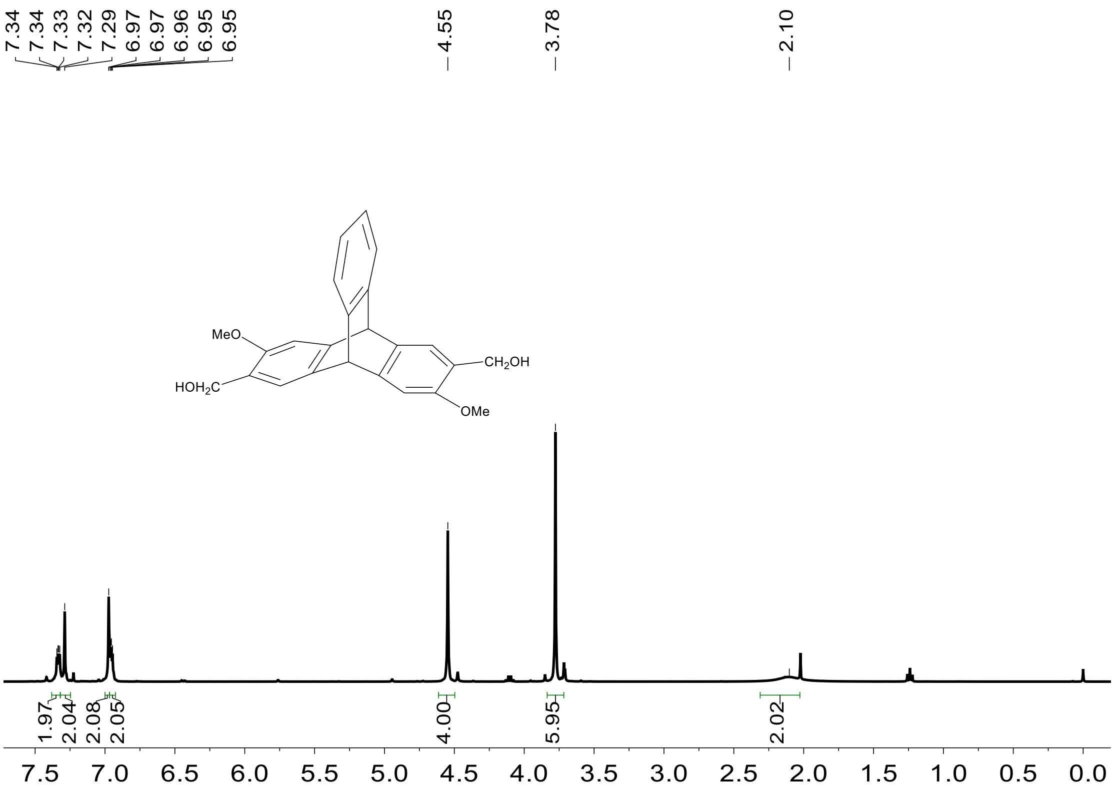

Figure S6. ^{1}H NMR spectrum (400 MHz, $CDCl_3$, 298 K) of *SS*-**1**.

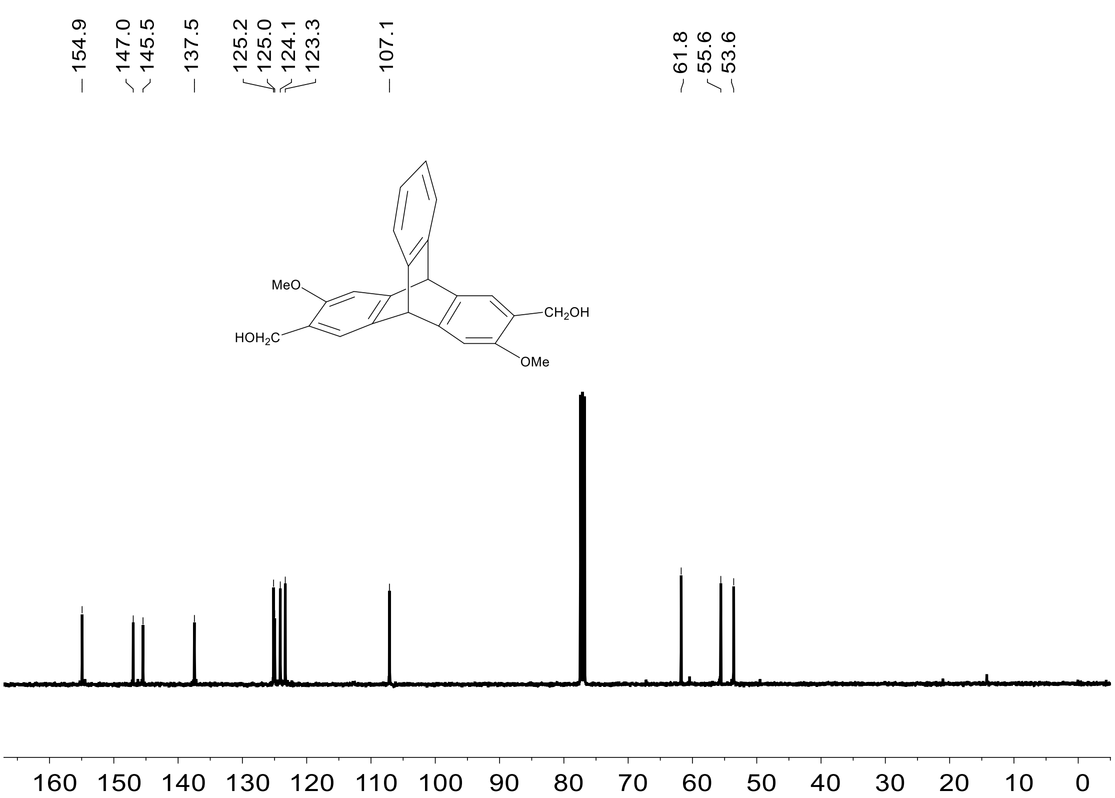

Figure S7. ^{13}C NMR spectrum (100 MHz, $CDCl_3$, 298 K) of *SS*-**1**.

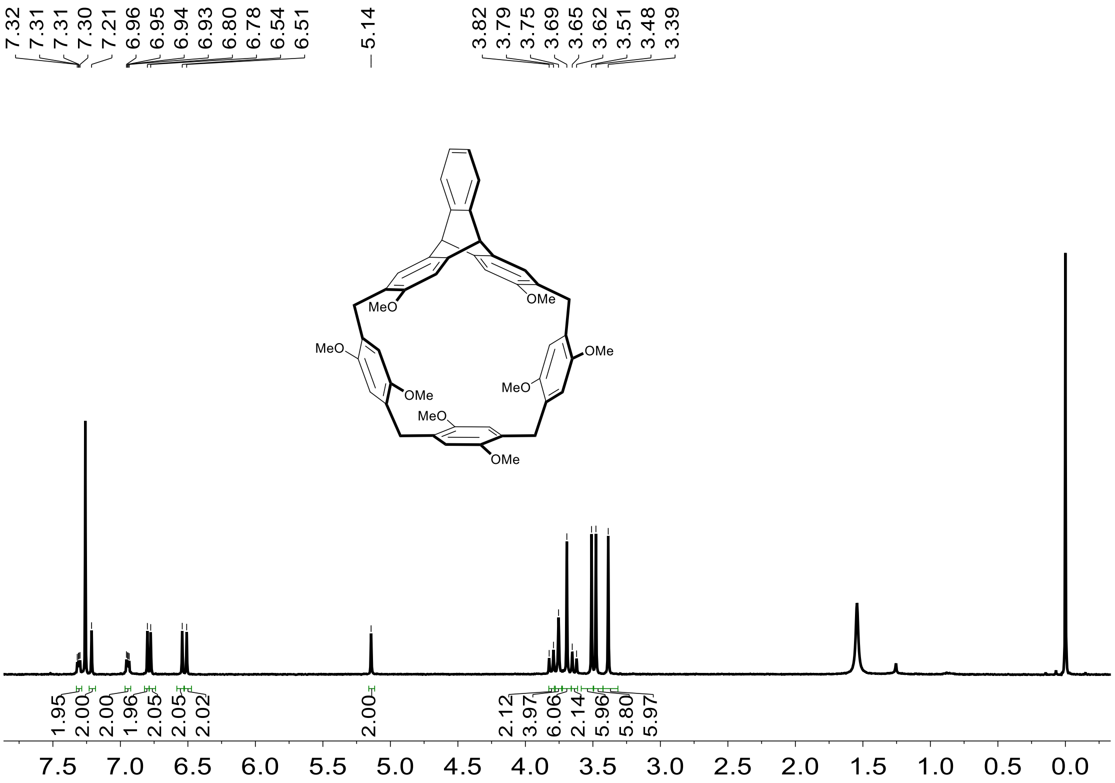

Figure S8. ^{1}H NMR spectrum (400 MHz, $CDCl_3$, 298 K) of *P*-**H**.

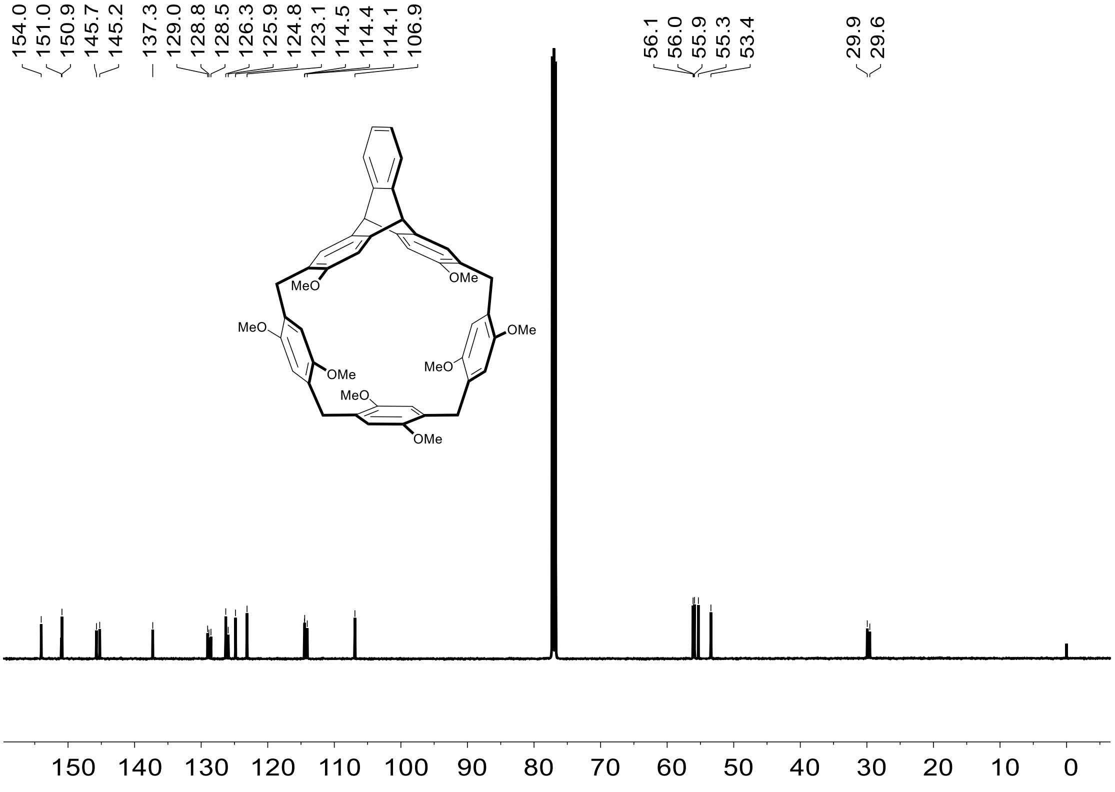

Figure S9. ^{13}C NMR spectrum (125 MHz, $CDCl_3$, 298 K) of *P*-**H**.

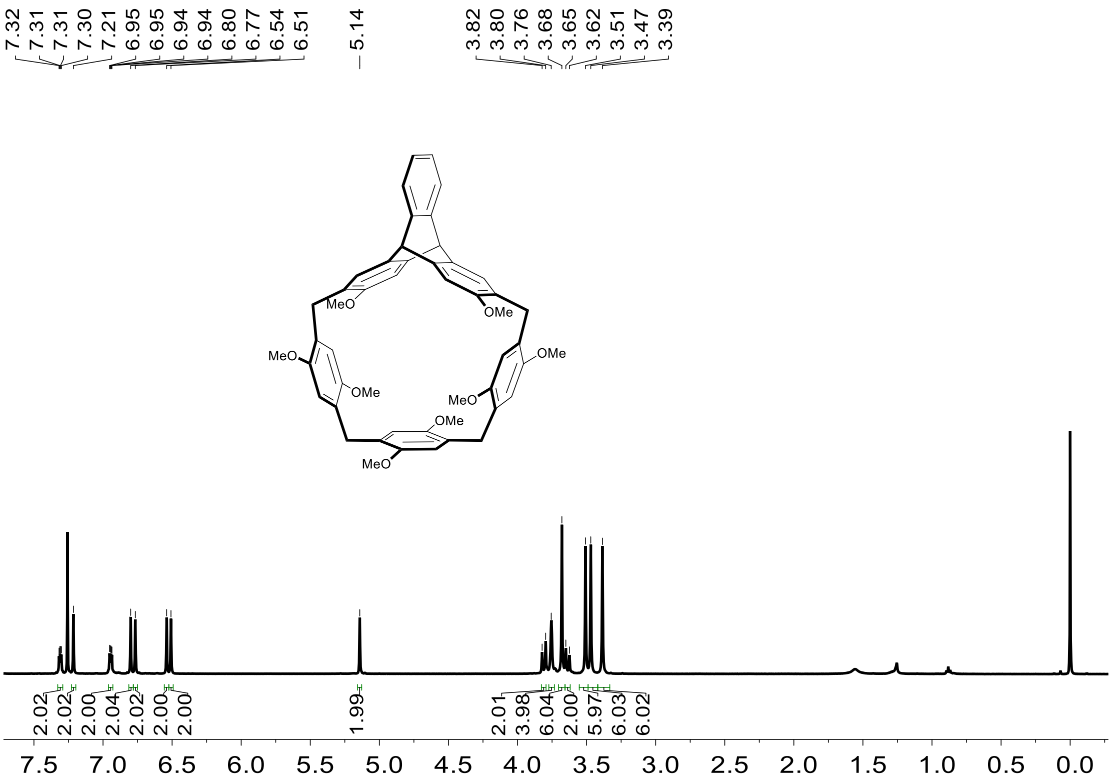

Figure S10. ^{1}H NMR spectrum (500 MHz, $CDCl_3$, 298 K) of *M*-**H**.

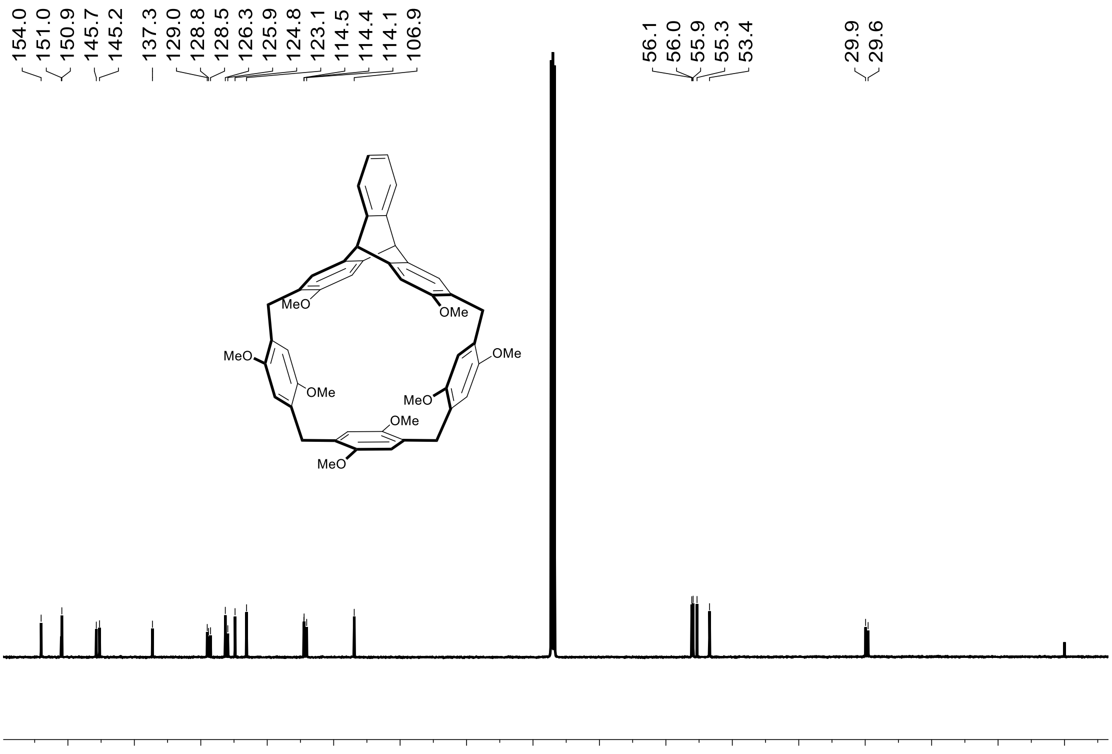

Figure S11. ^{13}C NMR spectrum (125 MHz, $CDCl_3$, 298 K) of *M*-**H**.

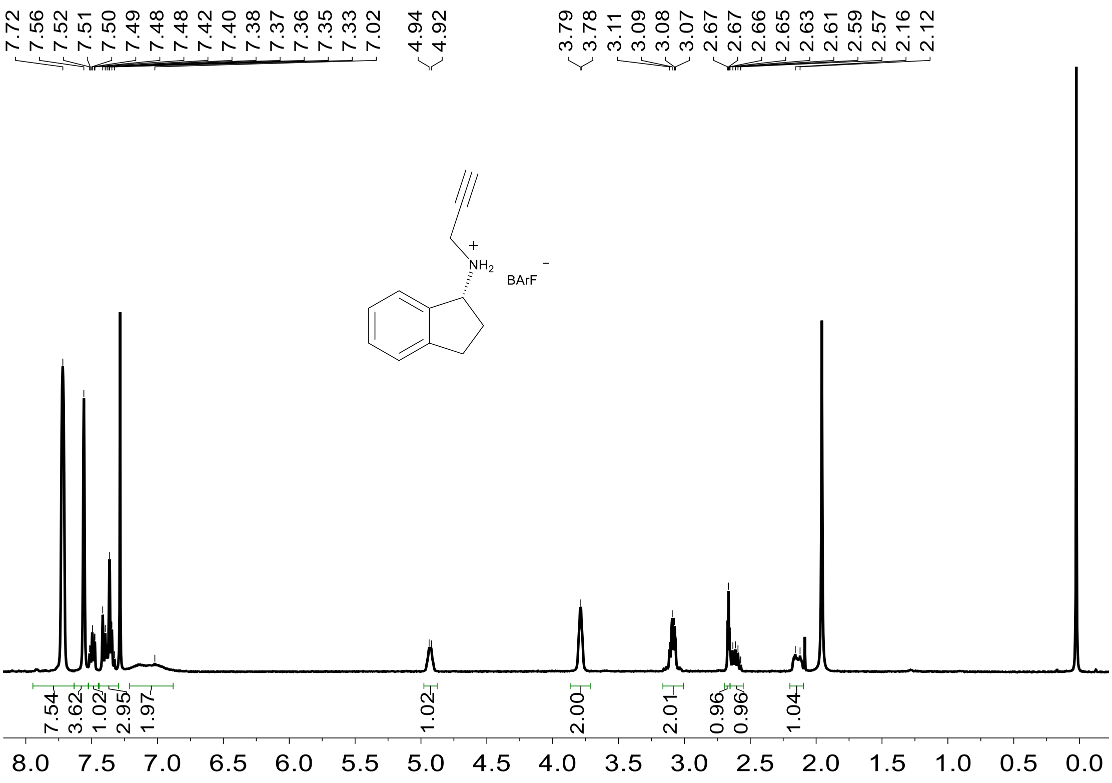

Figure S12. ^{1}H NMR spectrum (400 MHz, $CDCl_3$, 298 K) of *R*-**G1**.

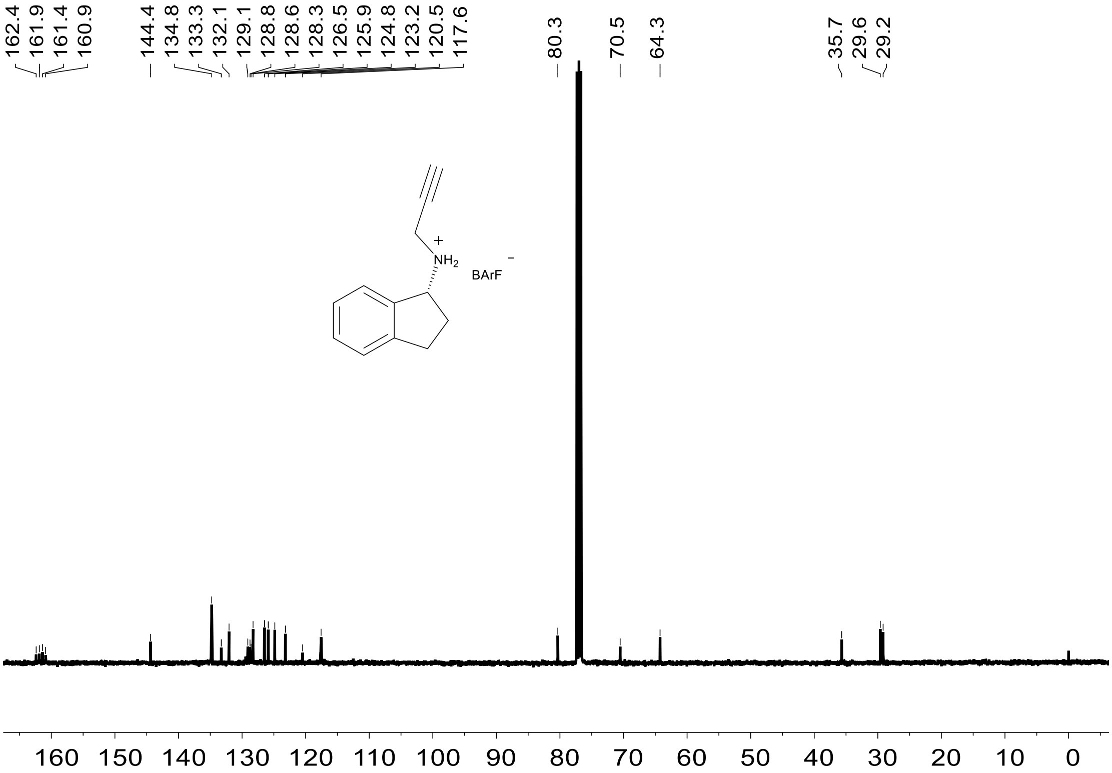

Figure S13. ^{13}C NMR spectrum (100 MHz, $CDCl_3$, 298 K) of *R*-**G1**.

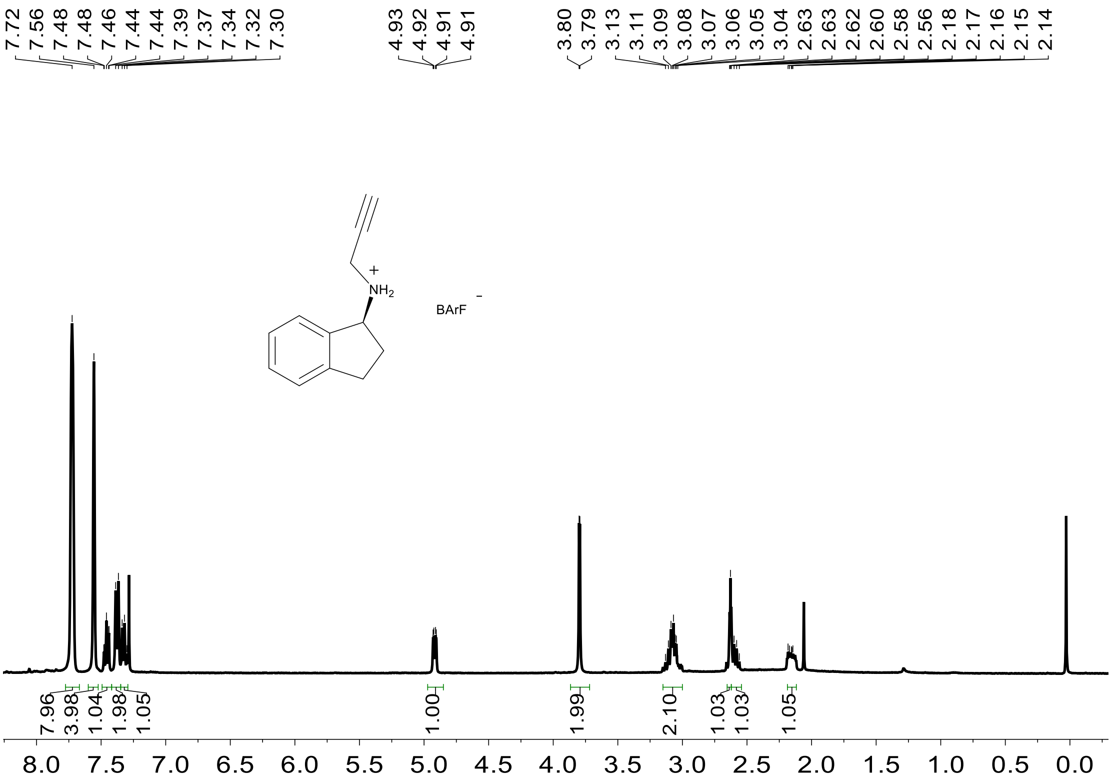

Figure S14. ^{1}H NMR spectrum (400 MHz, $CDCl_3$, 298 K) of *S*-**G1**.

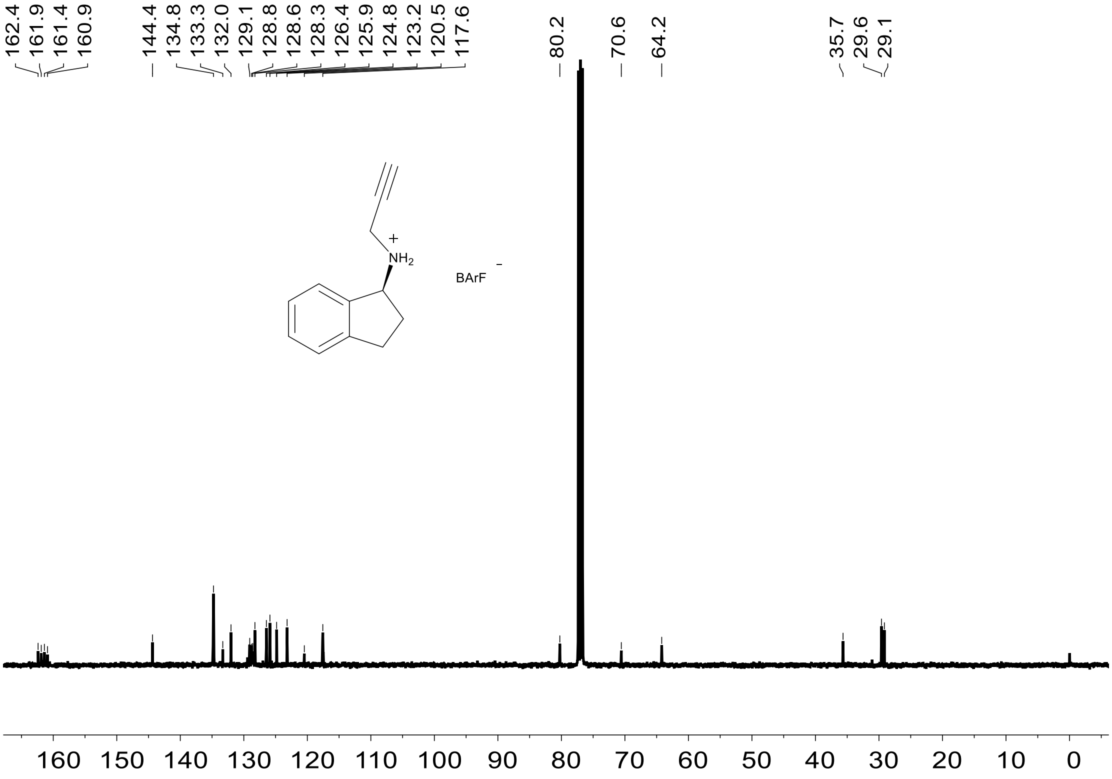

Figure S15. ^{13}C NMR spectrum (100 MHz, $CDCl_3$, 298 K) of *S*-**G1**.

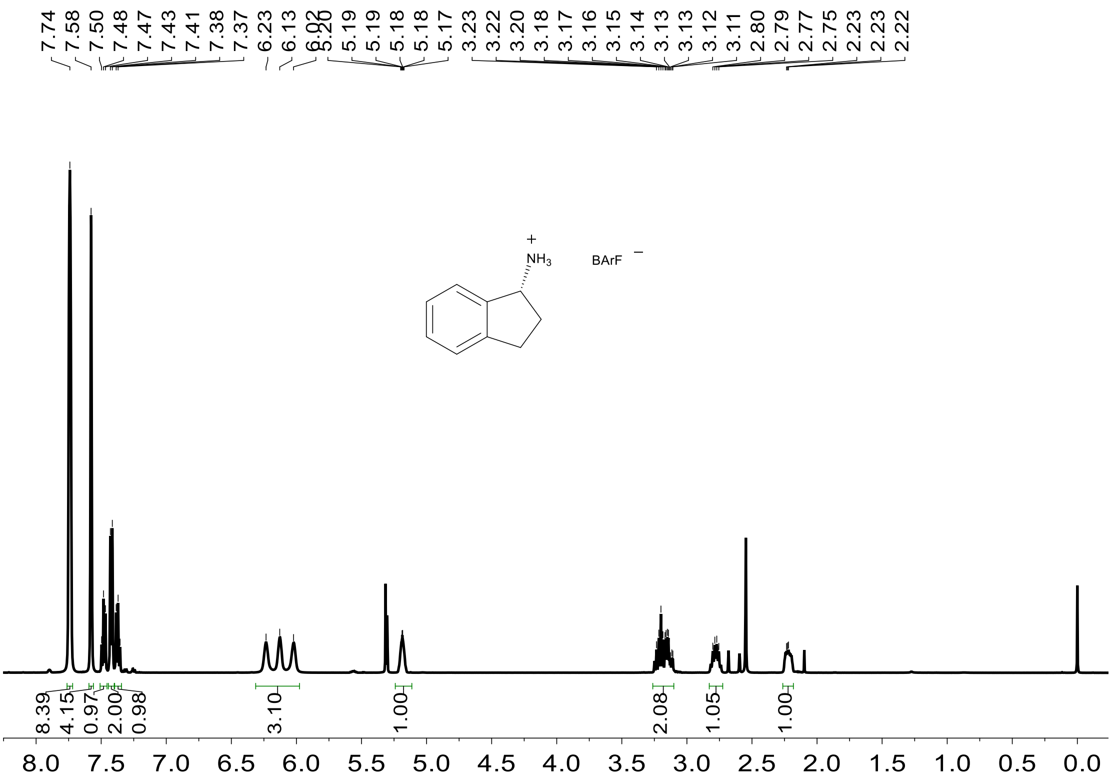

Figure S16. ^{1}H NMR spectrum (500 MHz, CD_2Cl_2, 298 K) of *R*-**G2**.

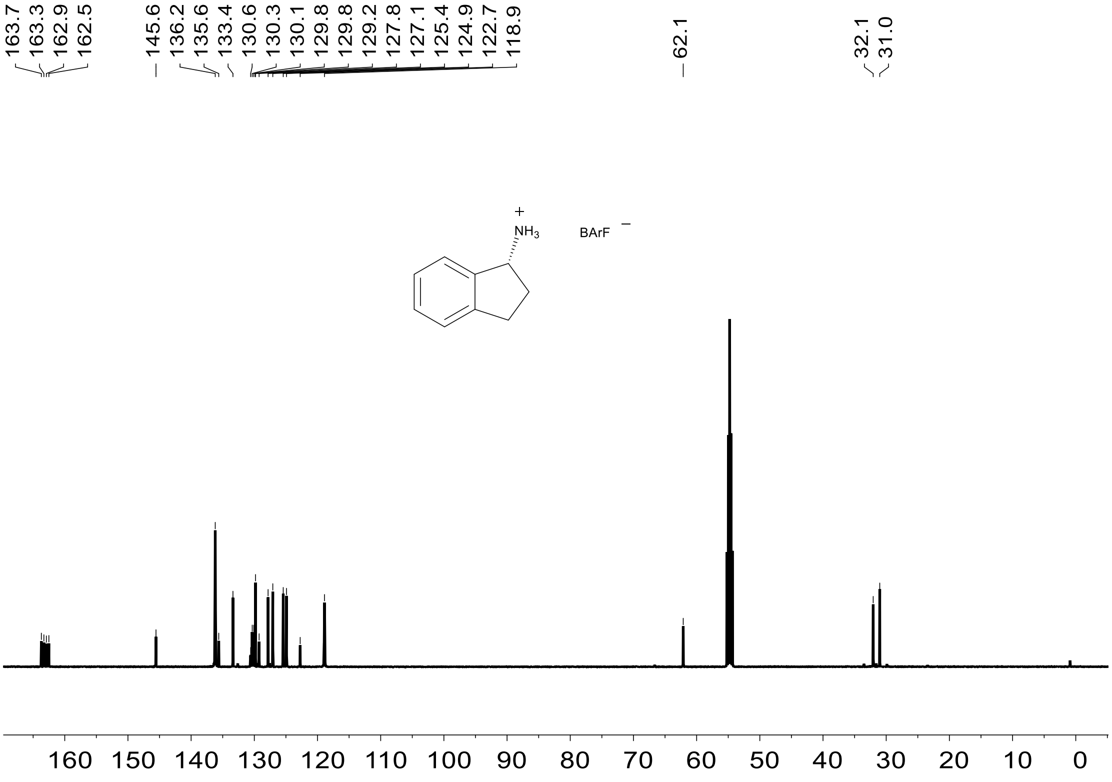

Figure S17. ^{13}C NMR spectrum (125 MHz, CD_2Cl_2, 298 K) of *R*-**G2**.

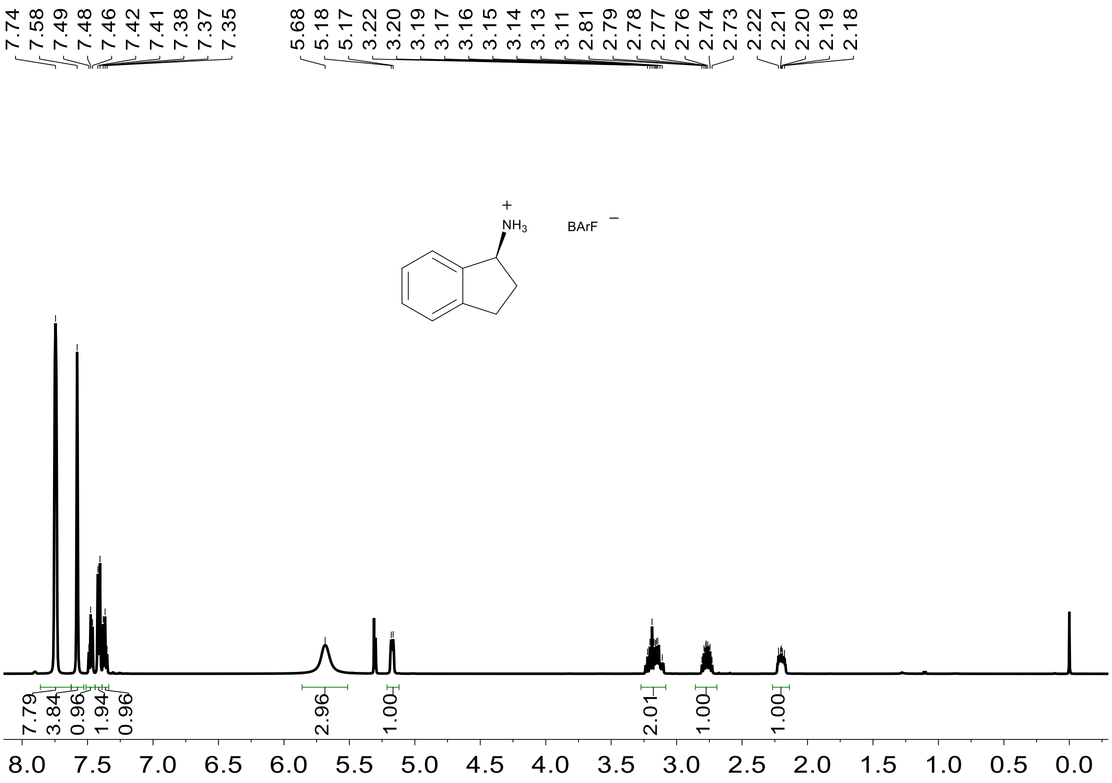

Figure S18. ^{1}H NMR spectrum (500 MHz, CD_2Cl_2, 298 K) of *S*-**G2**.

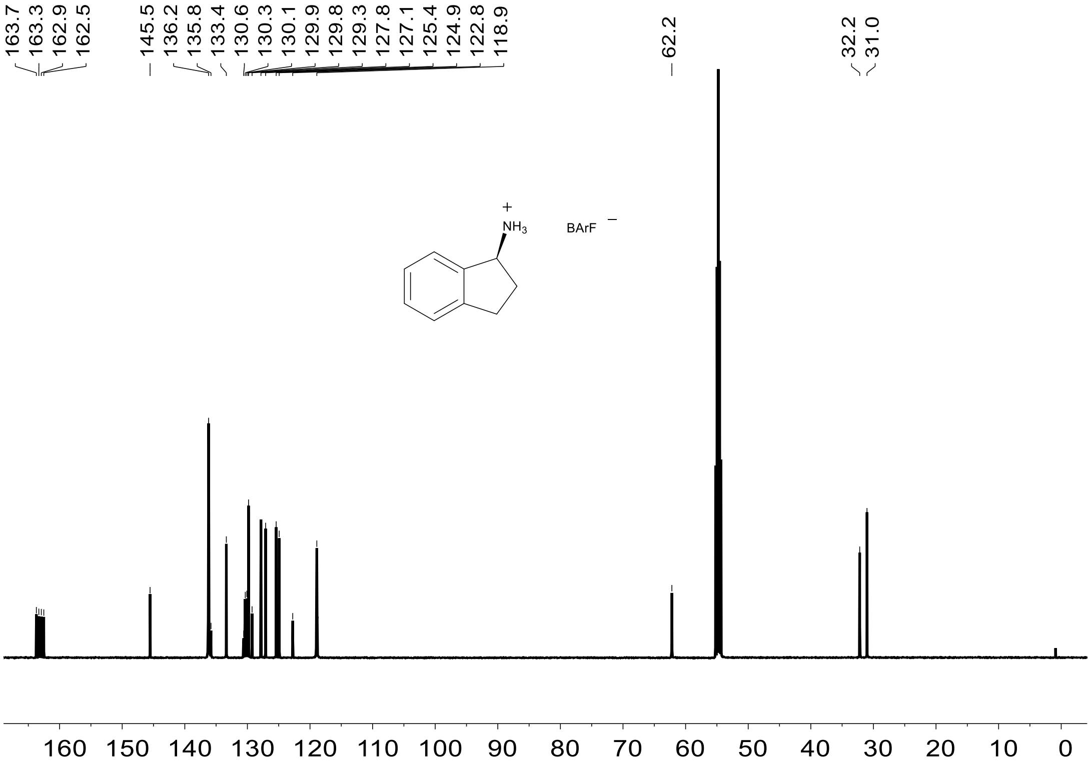

Figure S19. ^{13}C NMR spectrum (125 MHz, CD_2Cl_2, 298 K) of *S*-**G2**.

3. ^{1}H NMR Studies on the Complexation Between H and G1-G2

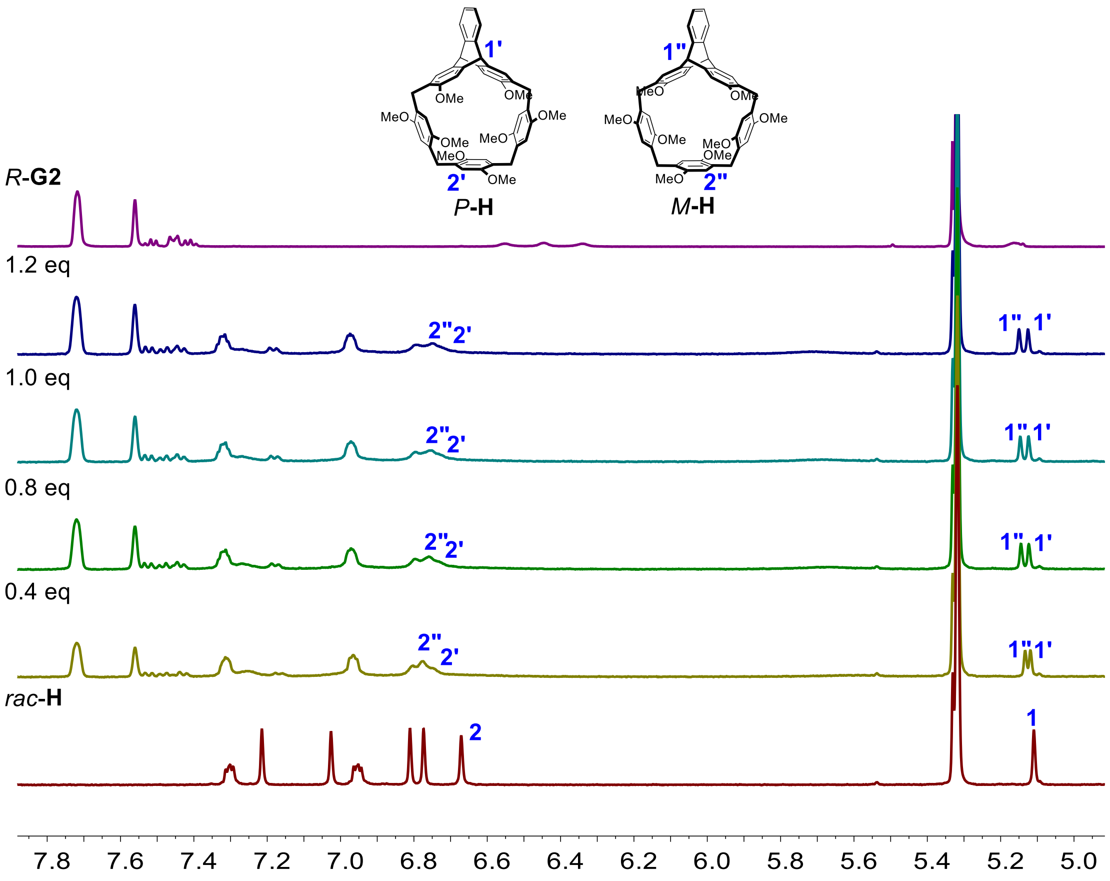

Figure S20. ^{1}H NMR spectra (500 MHz, 298K, CD_2Cl_2) of 2.0mM *rac*-**H** with different amounts of *R*-**G2**.

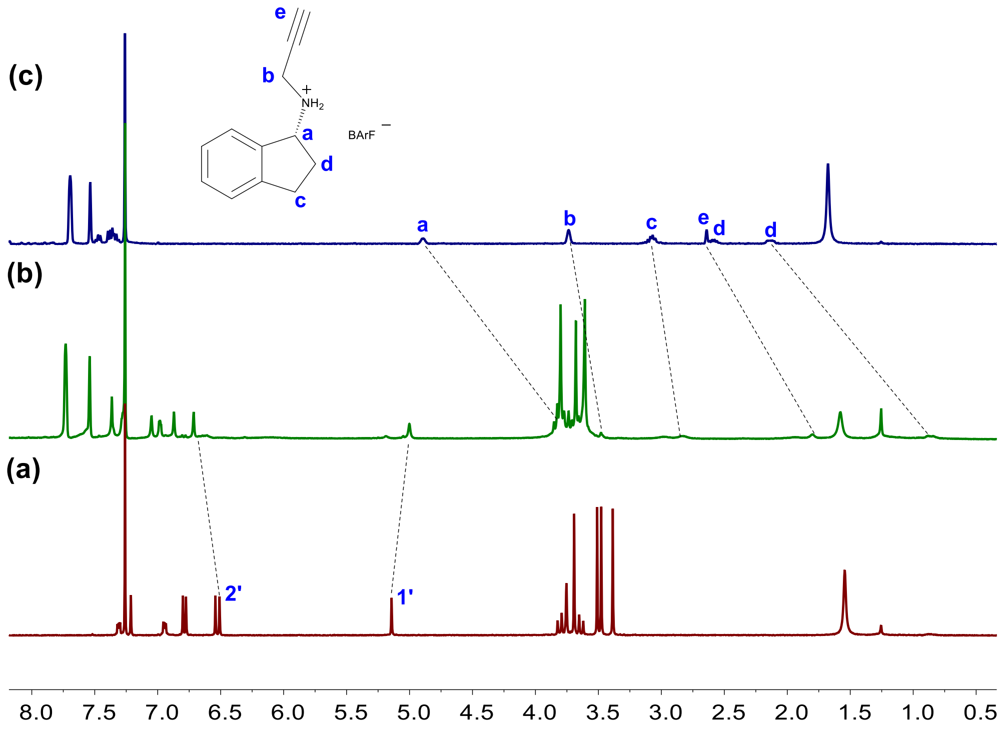

Figure S21. ^{1}H NMR spectra (500 MHz, 298K, $CDCl_3$) of (a) *P*-**H**, (b) *P*-**H** + 1.0 equiv. *R*-**G1**, (c) *R*-**G1**. [*P*-**H**]$_0$ = 3.00 mM.

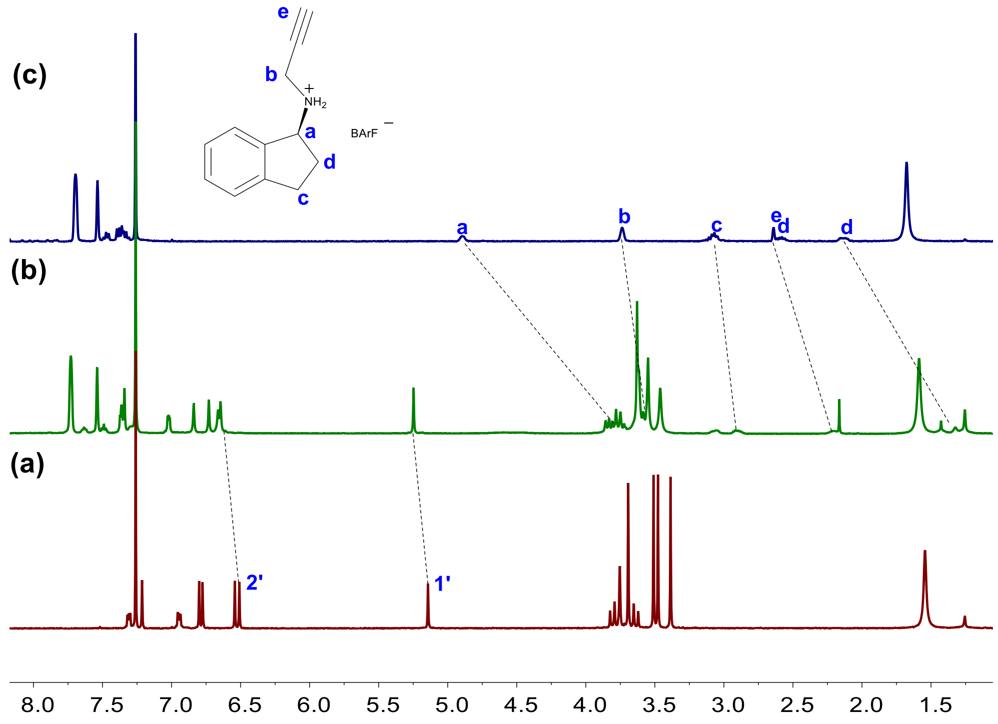

Figure S22. ^{1}H NMR spectra (500 MHz, 298K, $CDCl_3$) of (a) *P*-**H**, (b) *P*-**H** + 1.0 equiv. *S*-**G1**, (c) *S*-**G1**. [*P*-**H**]$_0$ = 3.00 mM.

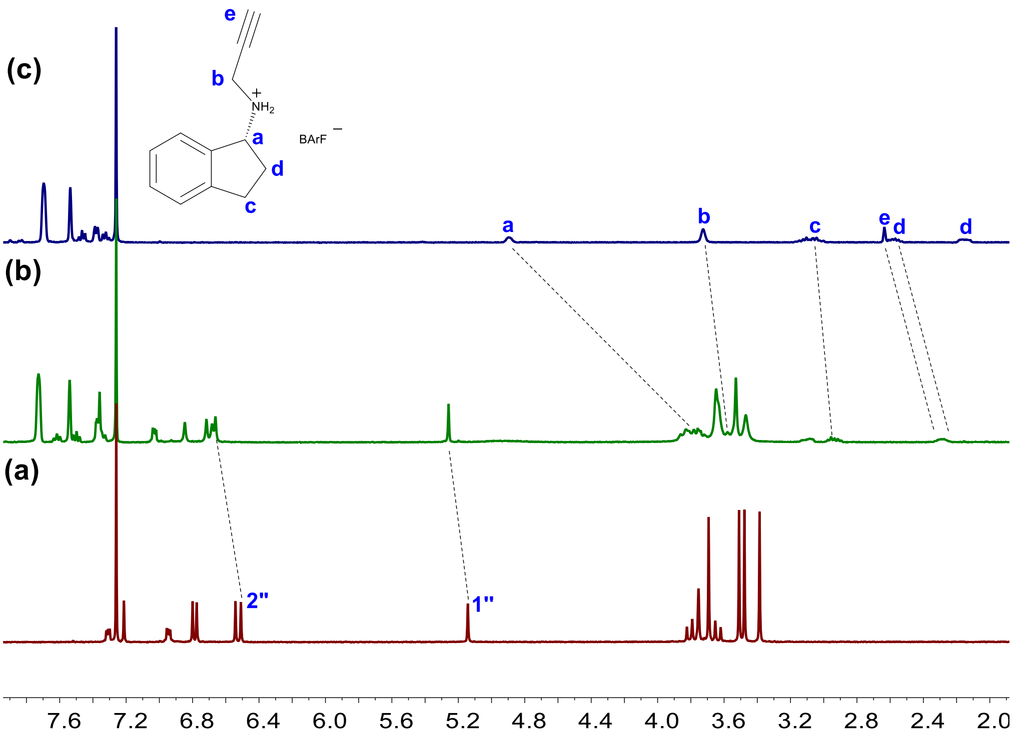

Figure S23. ^{1}H NMR spectra (500 MHz, 298K, $CDCl_3$) of (a) *M*-**H**, (b) *M*-**H** + 1.0 equiv. *R*-**G1**, (c) *R*-**G1**. $[M\text{-}\mathbf{H}]_0$ = 3.00 mM.

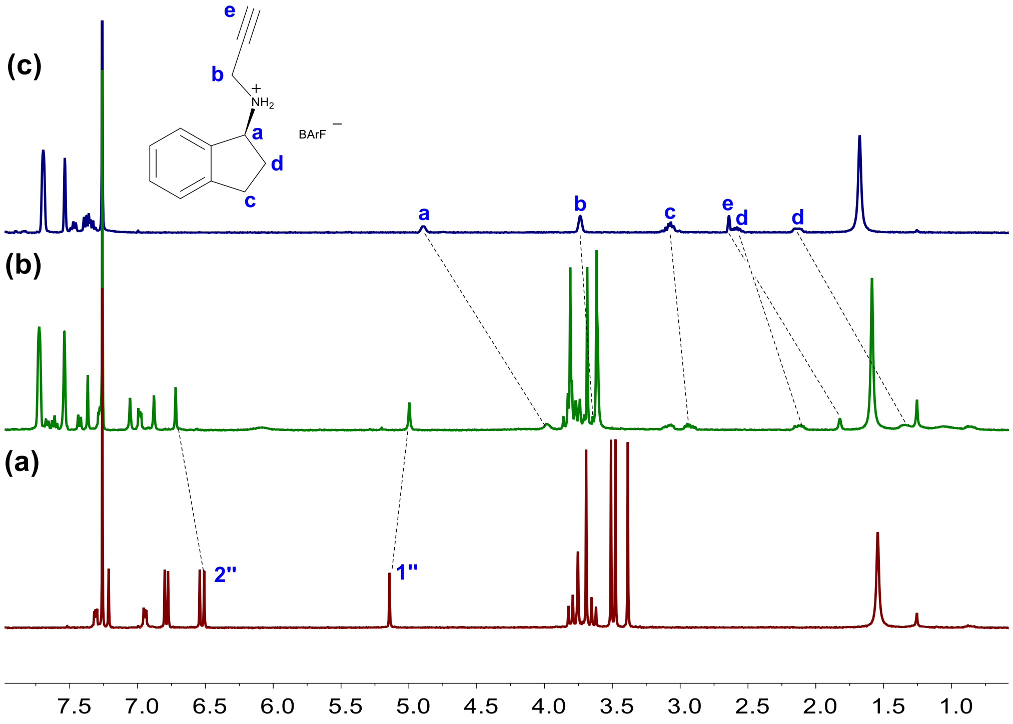

Figure S24. ^{1}H NMR spectra (500 MHz, 298K, $CDCl_3$) of (a) *M*-**H**, (b) *M*-**H** + 1.0 equiv. *S*-**G1**, (c) *S*-**G1**. $[M\text{-}\mathbf{H}]_0$ = 3.00 mM.

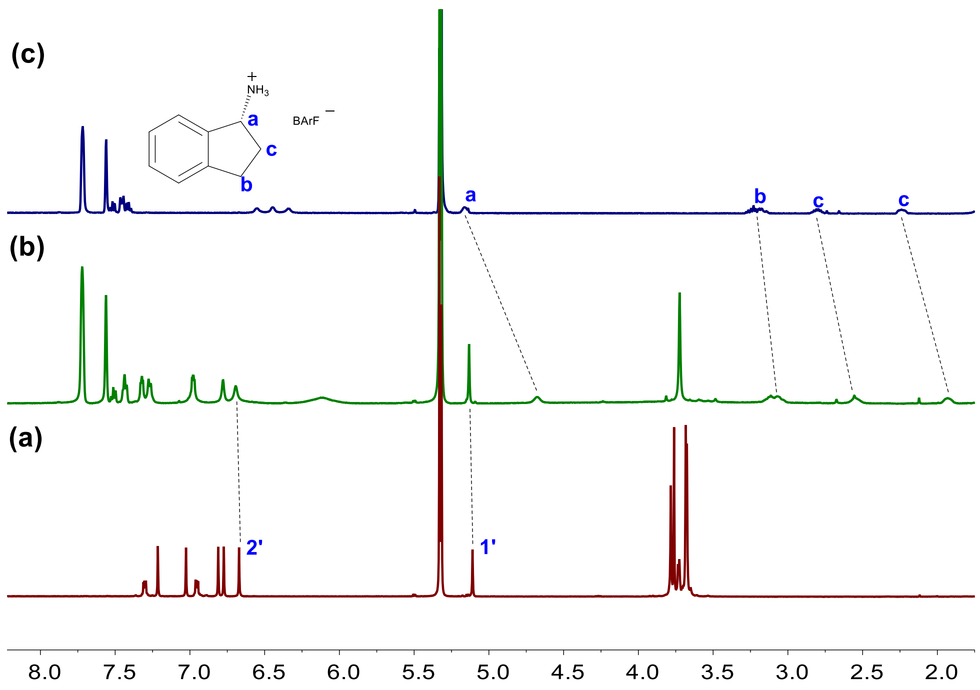

Figure S25. ^{1}H NMR spectra (500 MHz, 298K, CD_2Cl_2) of (a) *P*-**H**, (b) *P*-**H** + 1.0 equiv. *R*-**G2**, (c) *R*-**G2**. [*P*-**H**]$_0$ = 3.00 mM.

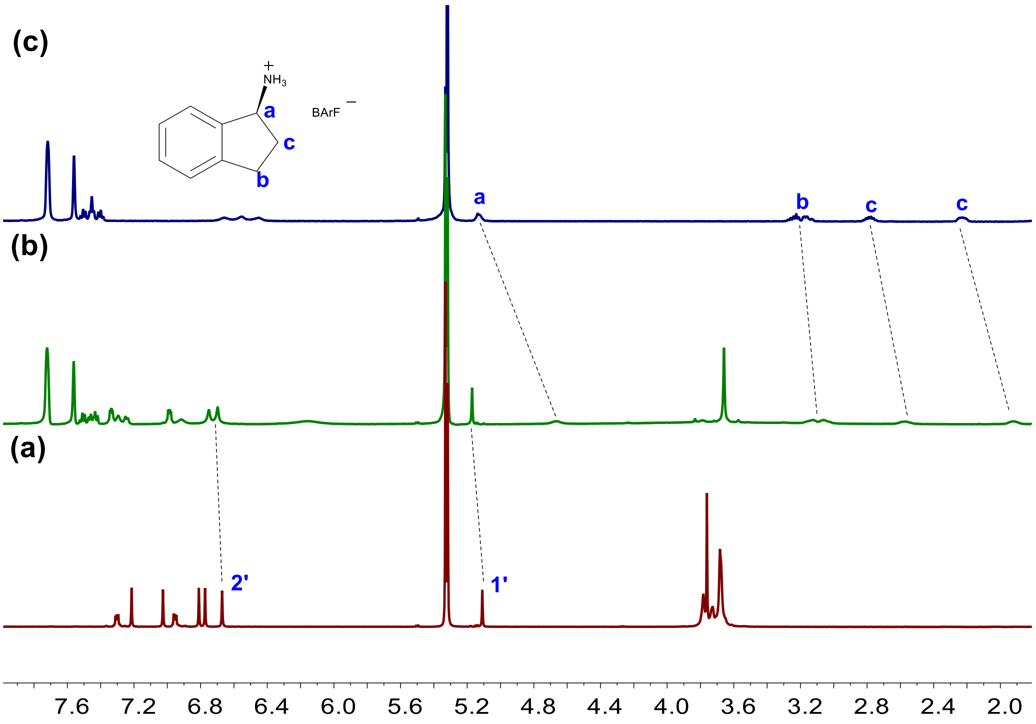

Figure S26. ^{1}H NMR spectra (500 MHz, 298K, CD_2Cl_2) of (a) *P*-**H**, (b) *P*-**H** + 1.0 equiv. *S*-**G2**, (c) *S*-**G2**. [*P*-**H**]$_0$ = 3.00 mM.

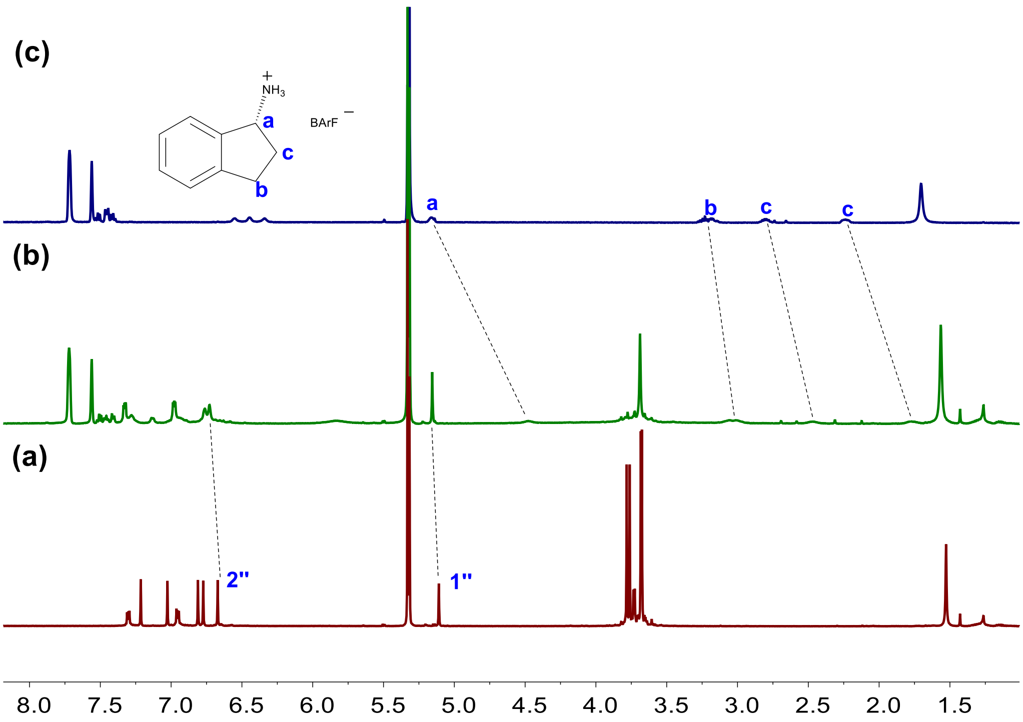

Figure S27. ^{1}H NMR spectra (500 MHz, 298K, CD_2Cl_2) of (a) *M*-**H**, (b) *M*-**H** + 1.0 equiv. *R*-**G2**, (c) *R*-**G2**. [*M*-**H**]$_0$ = 3.00 mM.

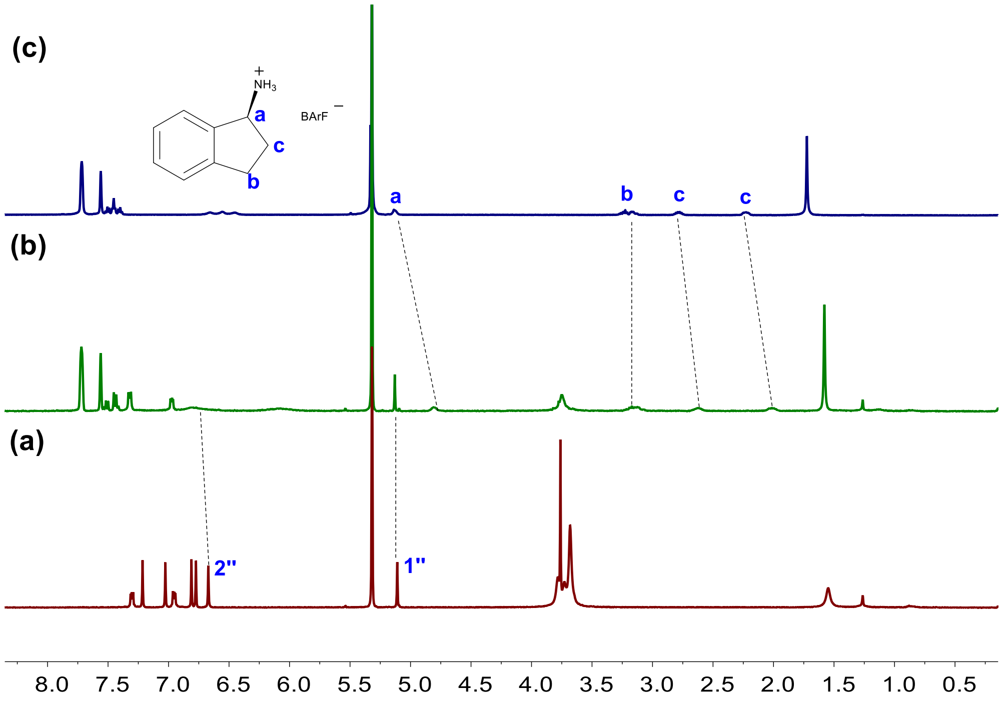

Figure S28. ^{1}H NMR spectra (500 MHz, 298K, CD_2Cl_2) of (a) *M*-**H**, (b) *M*-**H** + 1.0 equiv. *S*-**G2**, (c) *S*-**G2**. [*M*-**H**]$_0$ = 3.00 mM.

4. High Resolution Mass Spectra for the Complexes

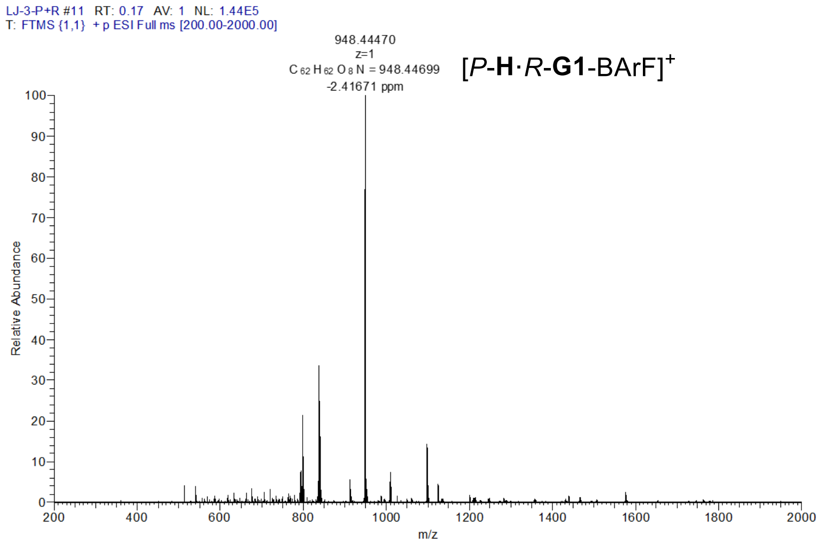

Figure S29. ESI-MS spectrum of the complex *P*-**H**·*R*-**G1**.

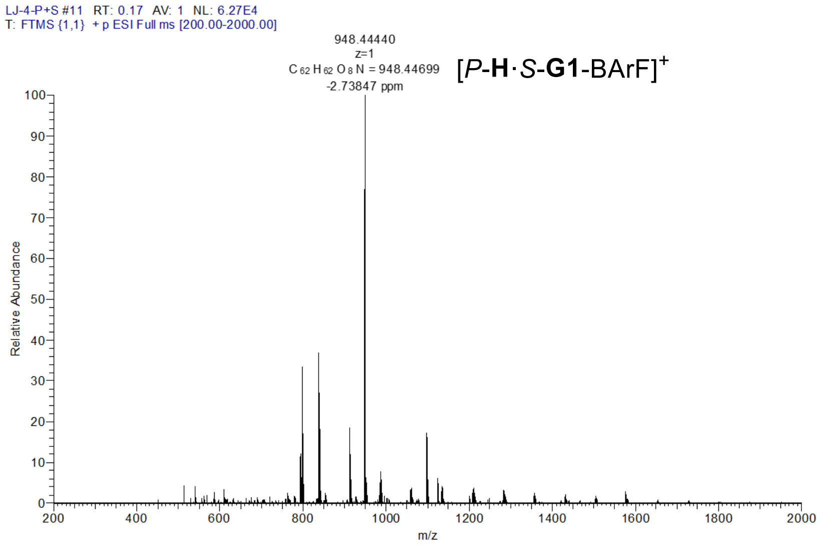

Figure S30. ESI-MS spectrum of the complex *P*-**H**·*S*-**G1**.

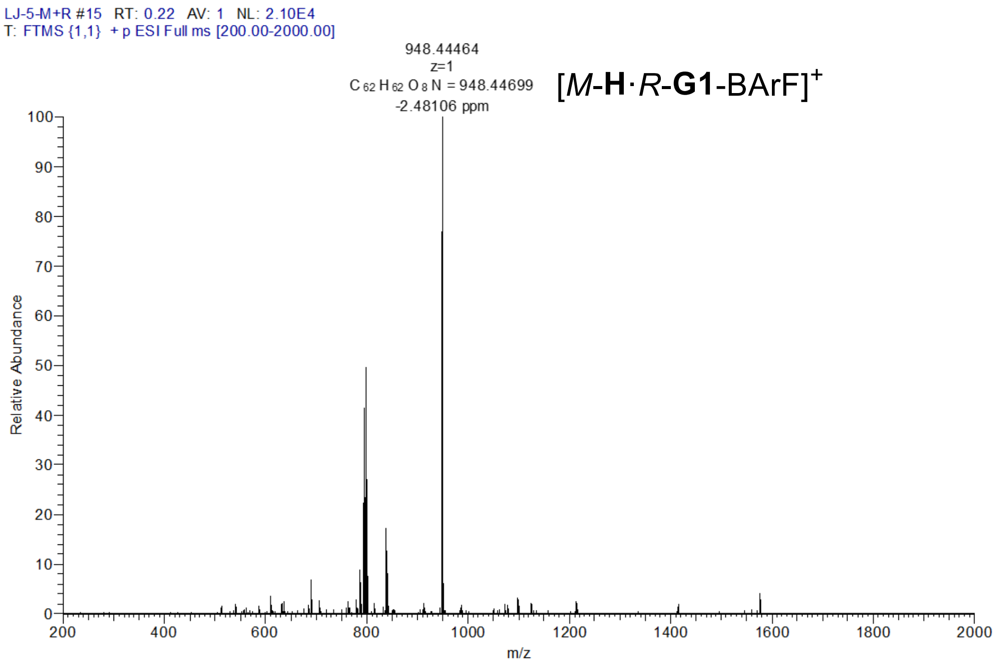

Figure S31. ESI-MS spectrum of the complex *M*-**H**·*R*-**G1**.

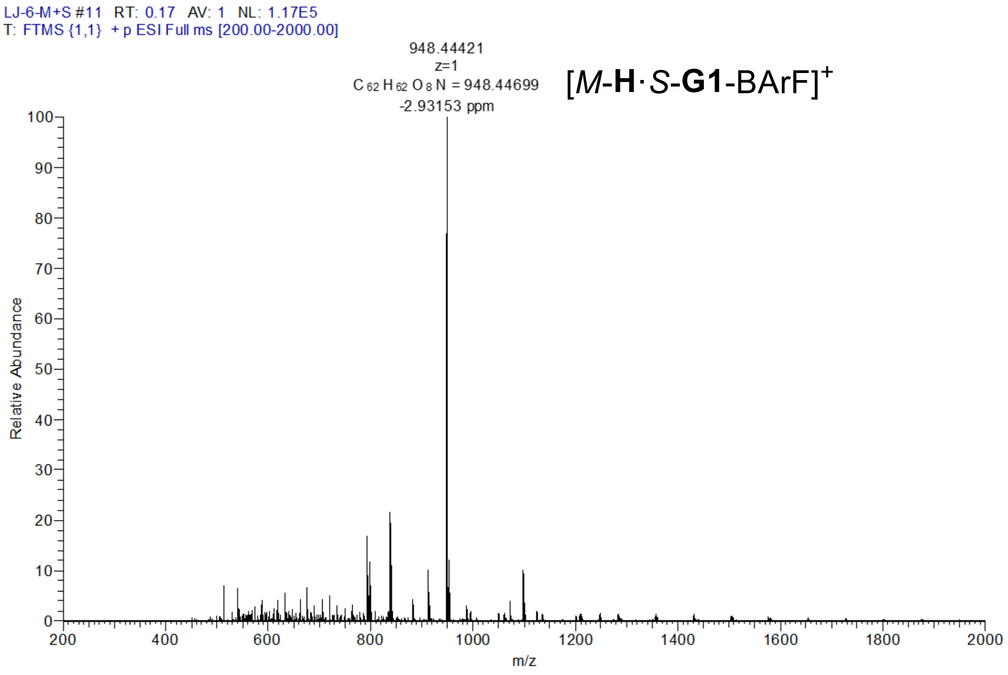

Figure S32. ESI-MS spectrum of the complex *M*-**H**·*S*-**G1**.

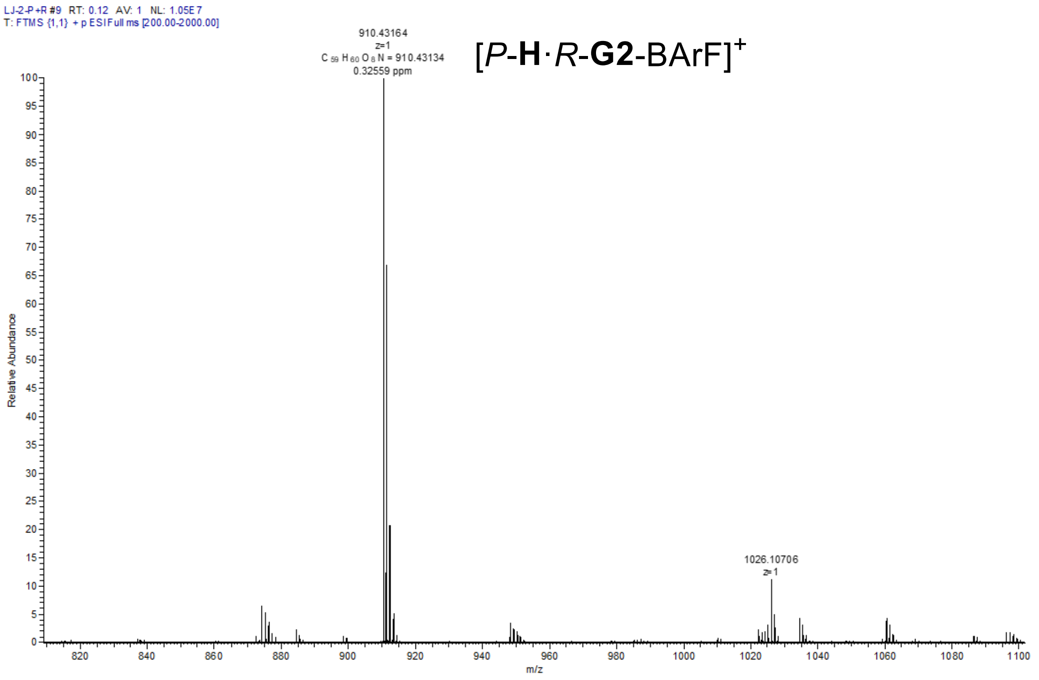

Figure S33. ESI-MS spectrum of the complex *P*-**H**·*R*-**G2**.

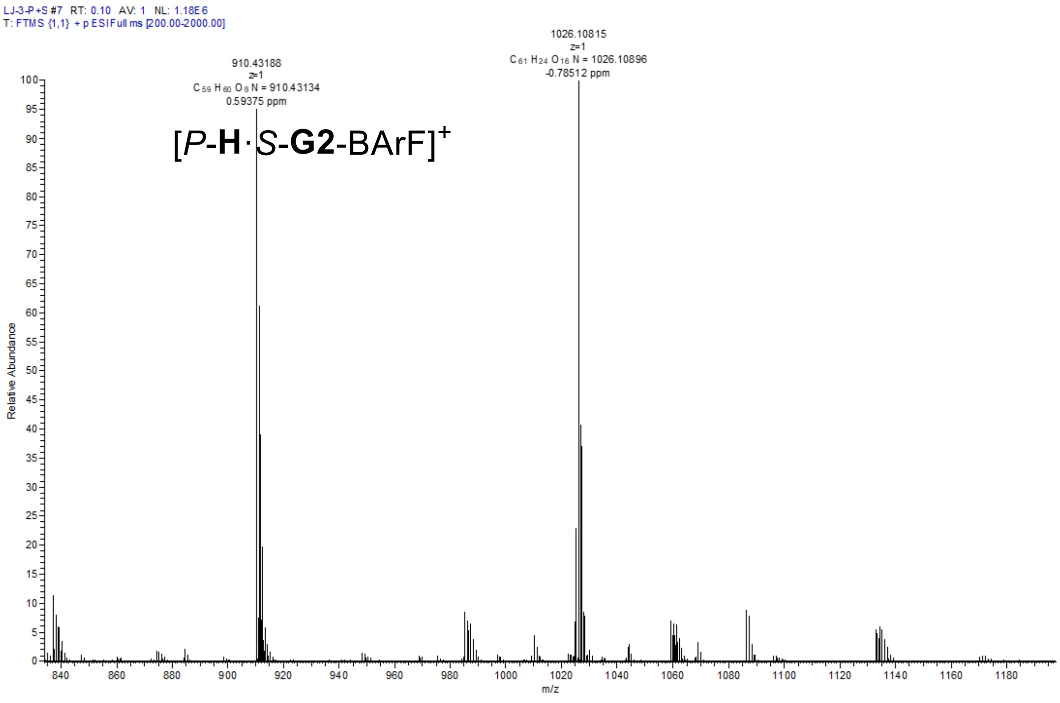

Figure S34. ESI-MS spectrum of the complex *P*-**H**·*S*-**G2**.

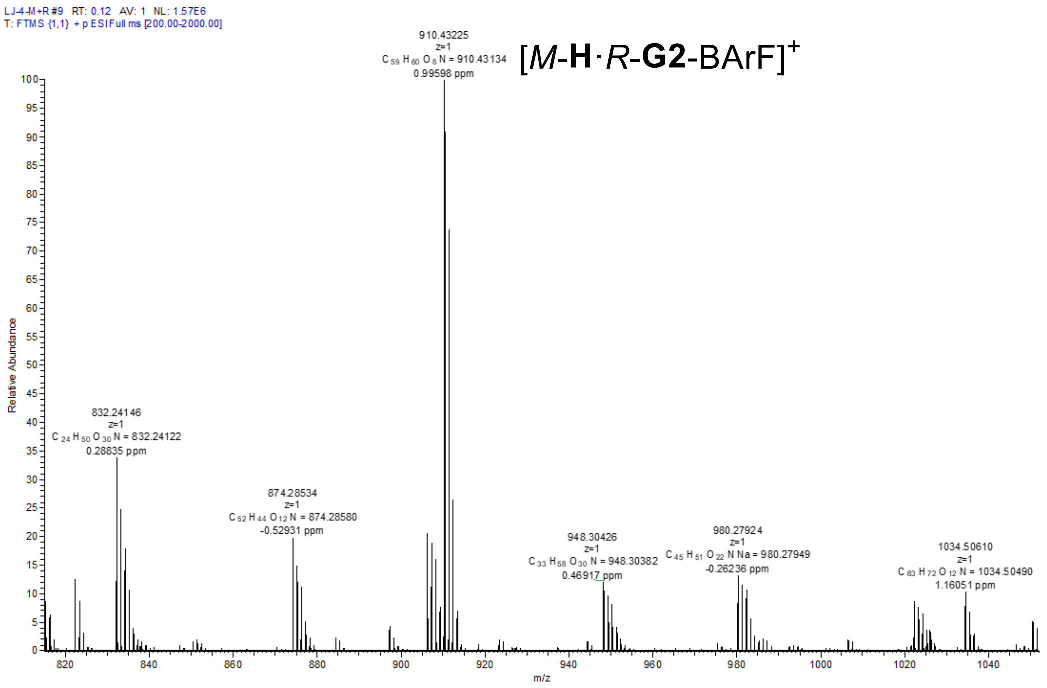

Figure S35. ESI-MS spectrum of the complex *M*-**H**·*R*-**G2**.

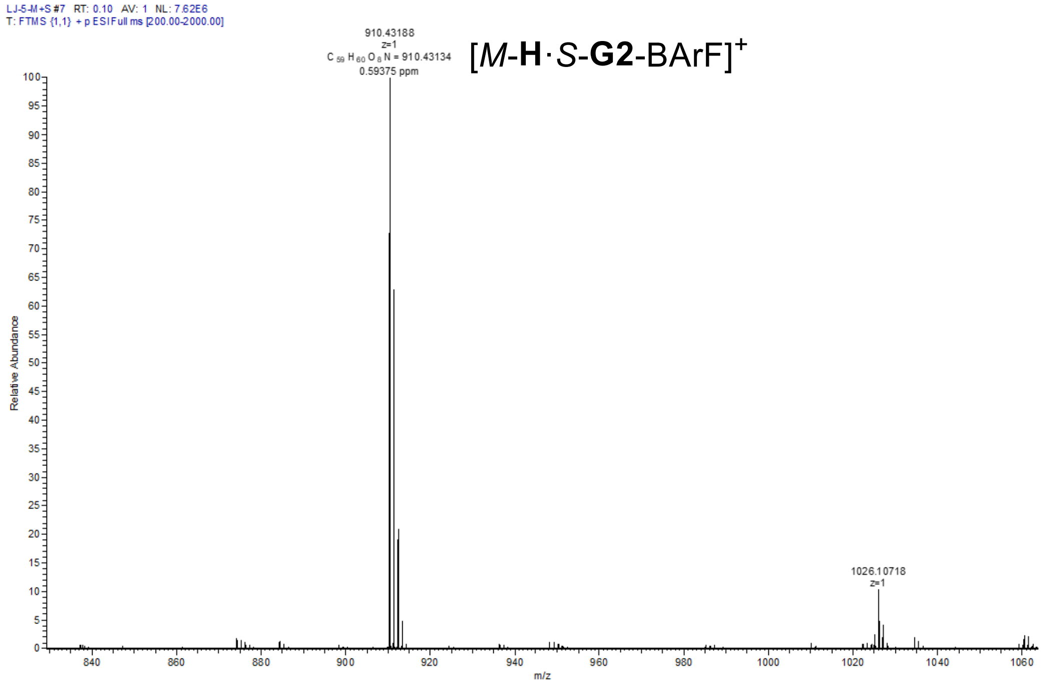

Figure S36. ESI-MS spectrum of the complex *M*-**H**·*S*-**G2**.

5. Determination of the Association Constants for the Complexes

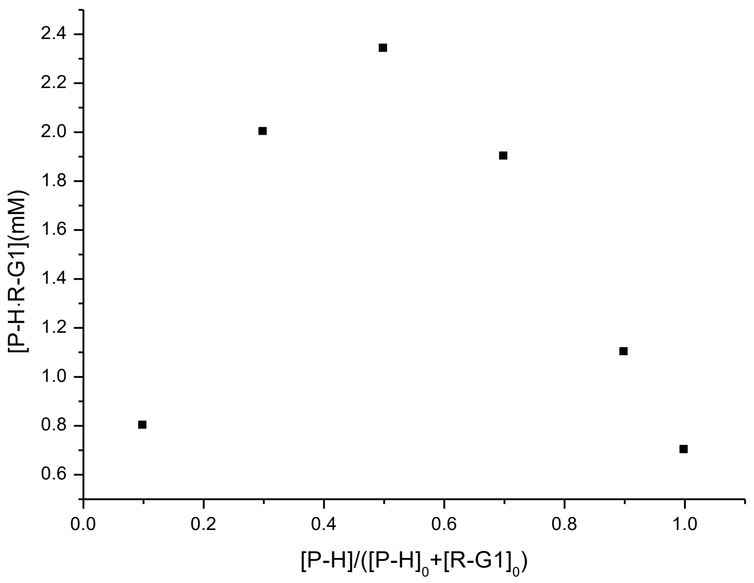

Figure S37. Job plot for the complexation of *P*-**H** and *R*-**G1** in $CDCl_3$ at 298 K.

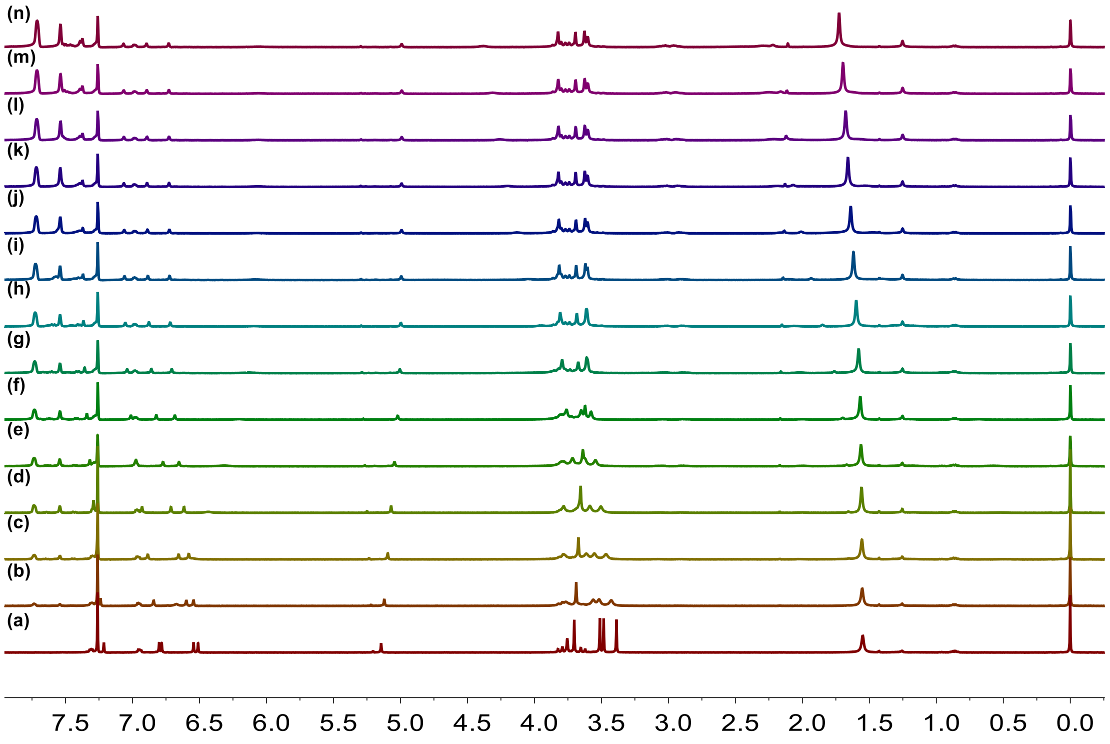

Figure S38. ^{1}H NMR spectra (400 MHz, 298K, $CDCl_3$) of *P*-**H** with different equivalents of *R*-**G1**: (a) 0.00, (b) 0.20, (c) 0.40, (d) 0.60, (e) 0.80, (f) 1.00, (g) 1.20, (h) 1.40, (i)1.60, (j) 1.80, (k) 2.00, (l) 2.20, (m) 2.40, (n) 2.50. [*P*-**H**]$_0$ = 3.00 mM.

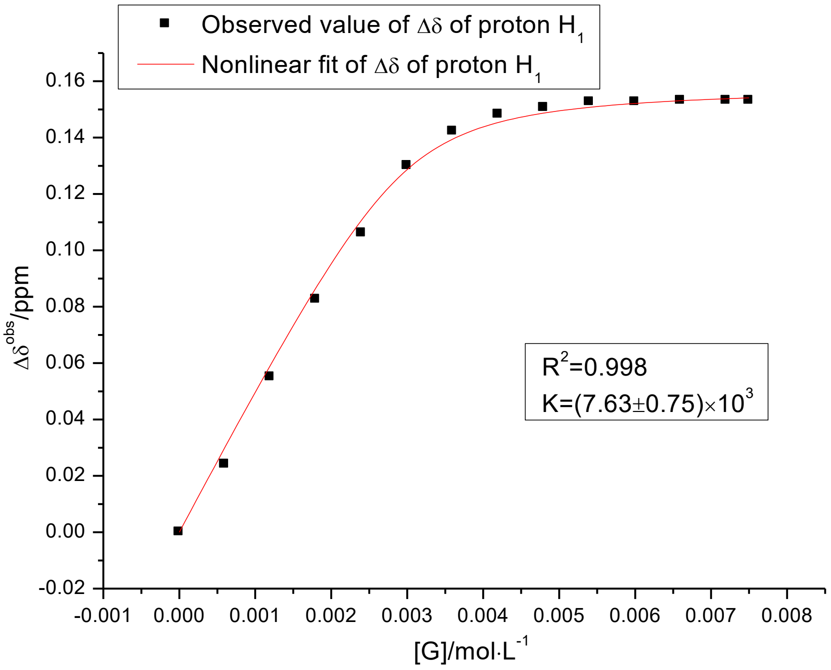

Figure S39. Plots of $\Delta\delta_{obs}$ (ppm) for the H_1 of ***P*-H** vs ***R*-G1** concentration in $CDCl_3$ at 298 K.

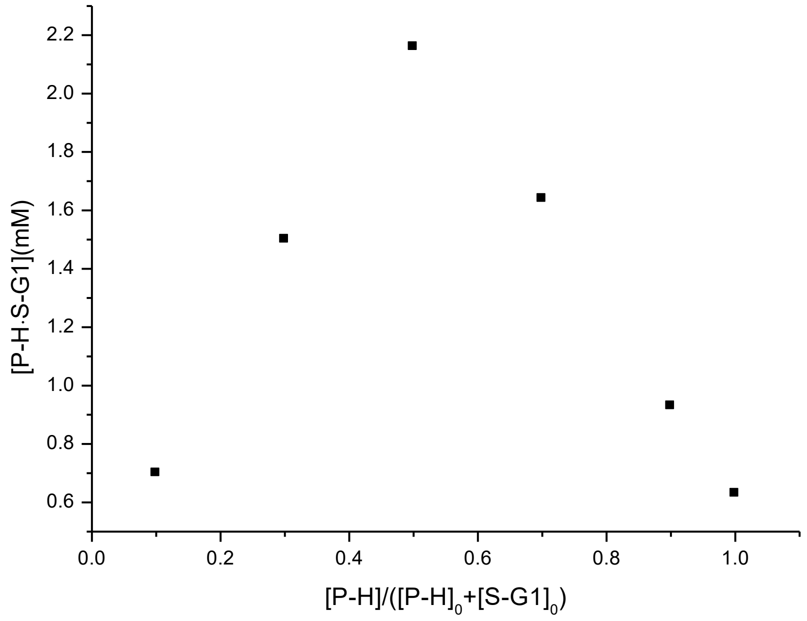

Figure S40. Job plot for the complexation of ***P*-H** and ***S*-G1** in $CDCl_3$ at 298 K.

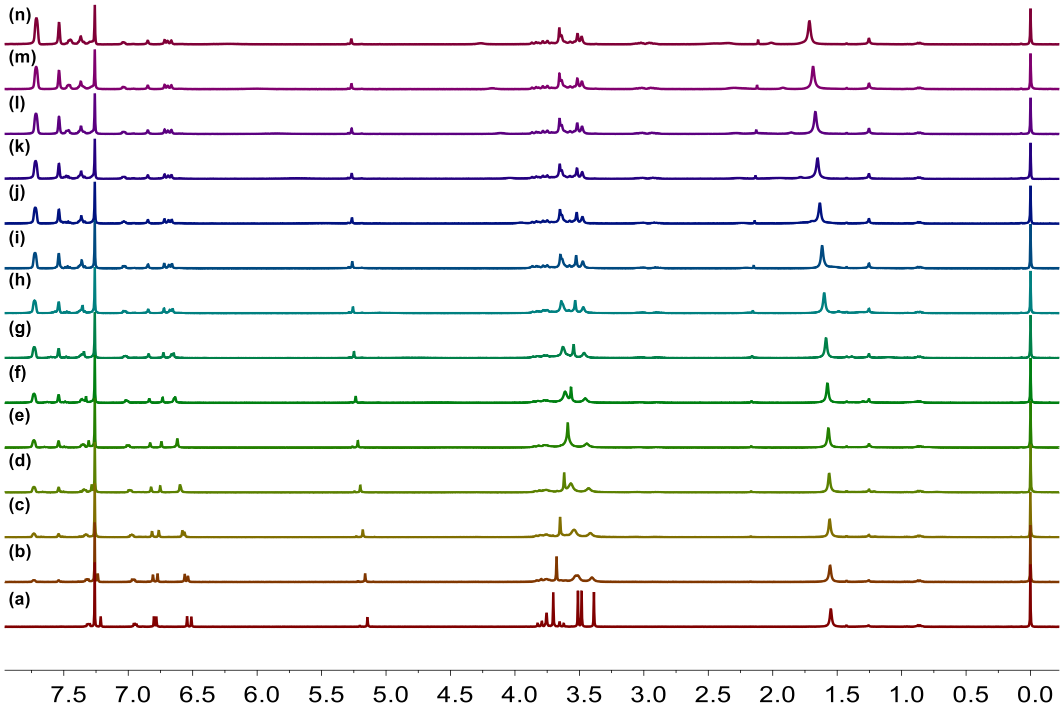

Figure S41. ^{1}H NMR spectra (400 MHz, 298K, $CDCl_3$) of *P*-**H** with different equivalents of *S*-**G1**: (a) 0.00, (b) 0.20, (c) 0.40, (d) 0.60, (e) 0.80, (f) 1.00, (g) 1.20, (h) 1.40, (i)1.60, (j) 1.80, (k) 2.00, (l) 2.20, (m) 2.40, (n) 2.50. $[P\text{-}\mathbf{H}]_0$ = 3.00 mM.

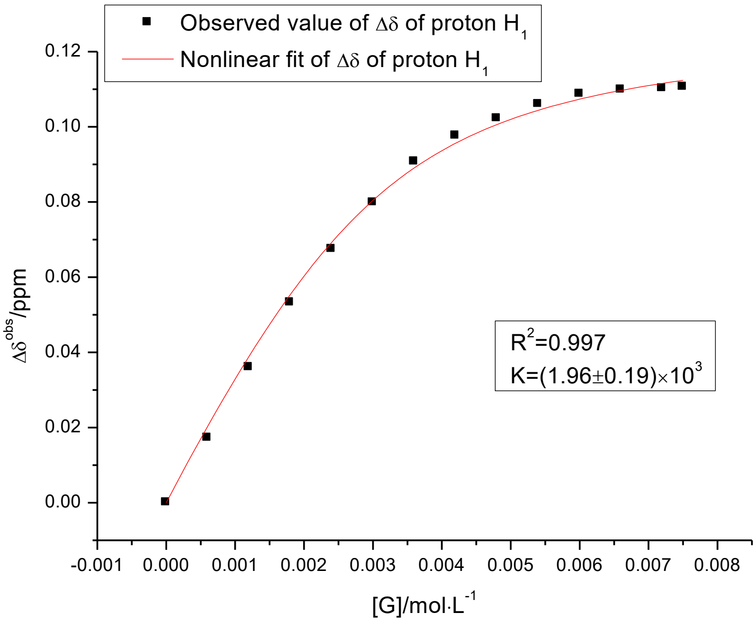

Figure S42. Plots of $\Delta\delta_{obs}$ (ppm) for the H_{13} of *P*-**H** vs *S*-**G1** concentration in $CDCl_3$ at 298 K.

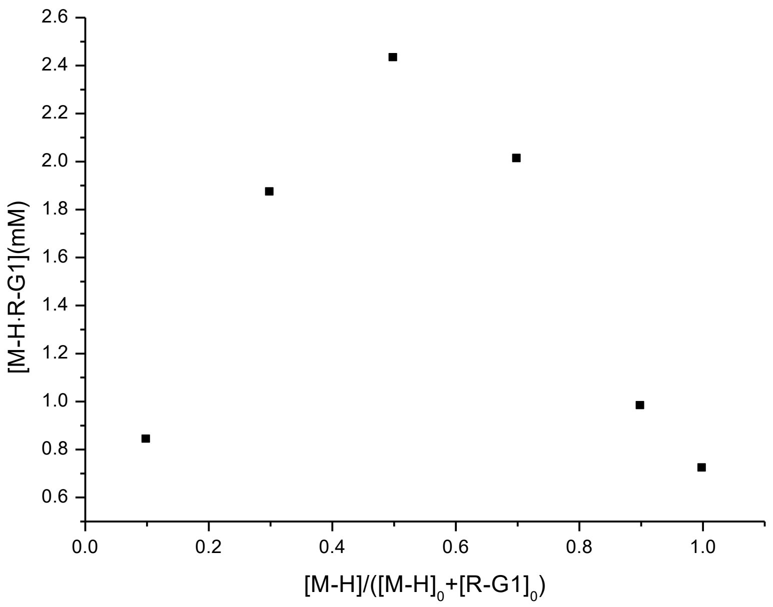

Figure S43. Job plot for the complexation of *M*-**H** and *R*-**G1** in $CDCl_3$ at 298 K.

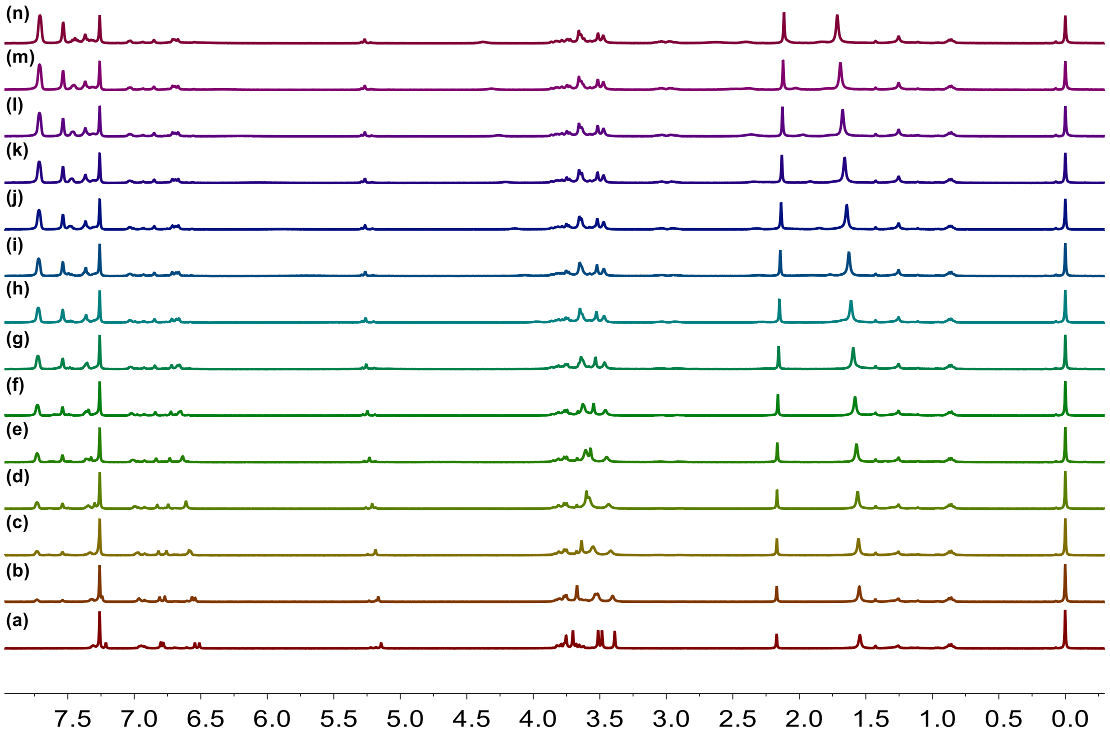

Figure S44. ^{1}H NMR spectra (400 MHz, 298K, $CDCl_3$) of *M*-**H** with different equivalents of *R*-**G1**: (a) 0.00, (b) 0.20, (c) 0.40, (d) 0.60, (e) 0.80, (f) 1.00, (g) 1.20, (h) 1.40, (i)1.60, (j) 1.80, (k) 2.00, (l) 2.20, (m) 2.40, (n) 2.50. [*M*-**H**]$_0$ = 3.00 mM.

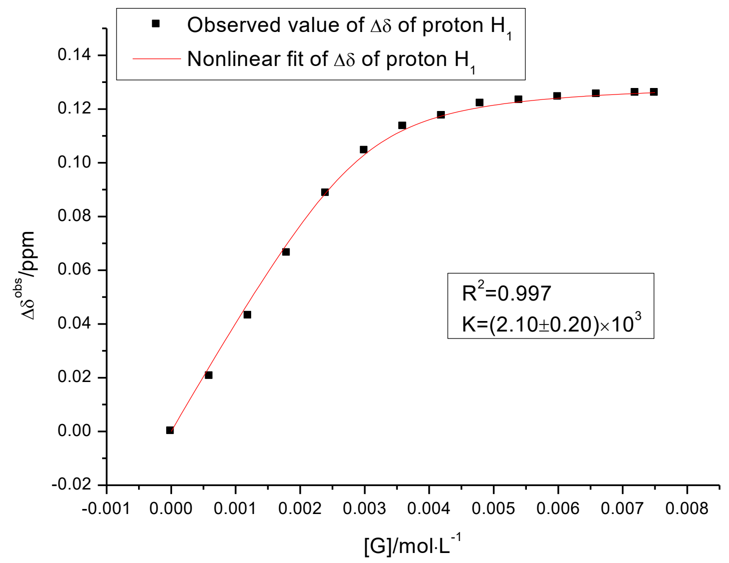

Figure S45. Plots of $\Delta\delta_{obs}$ (ppm) for the H_1 of *M*-**H** vs *R*-**G1** concentration in $CDCl_3$ at 298 K.

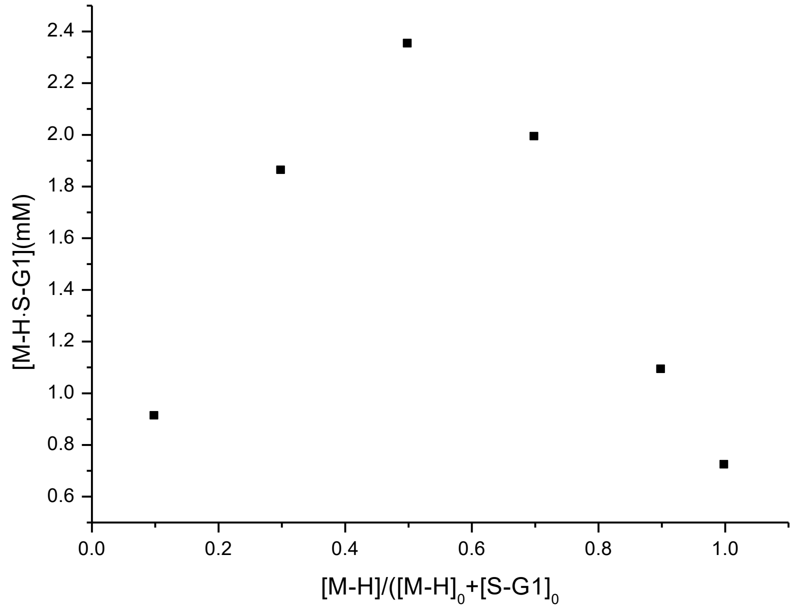

Figure S46. Job plot for the complexation of *M*-**H** and *S*-**G1** in $CDCl_3$ at 298 K.

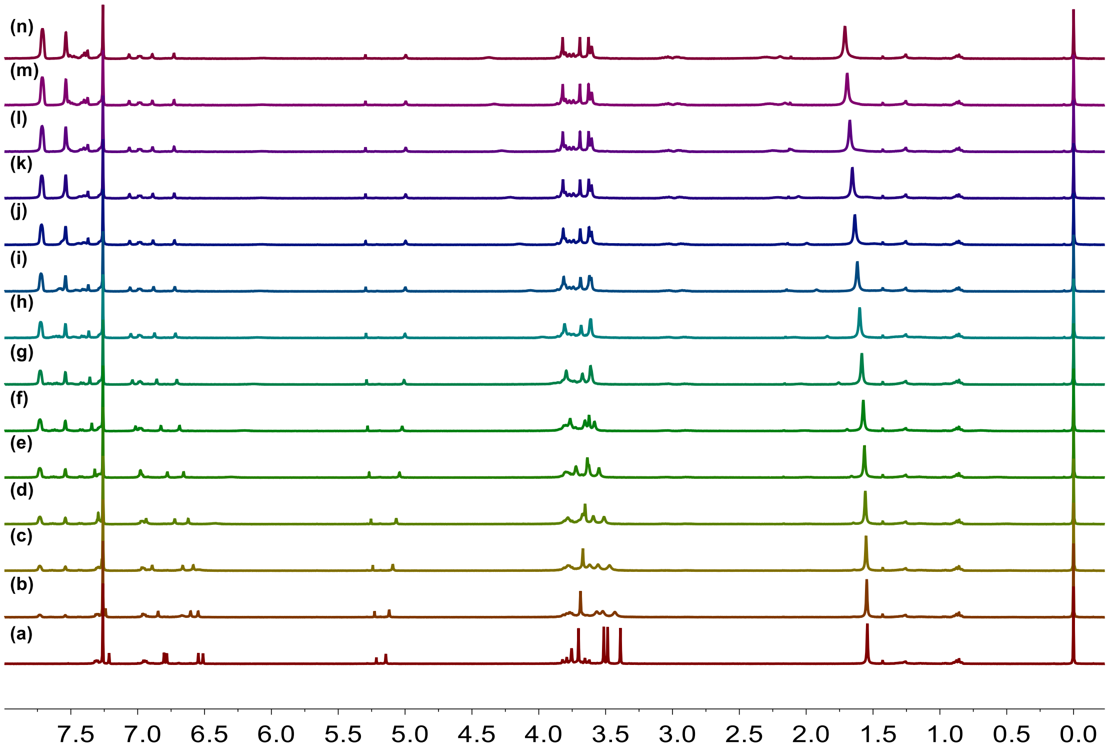

Figure S47. ^{1}H NMR spectra (400 MHz, 298K, $CDCl_3$) of *M*-**H** with different equivalents of *S*-**G1**: (a) 0.00, (b) 0.20, (c) 0.40, (d) 0.60, (e) 0.80, (f) 1.00, (g) 1.20, (h) 1.40, (i)1.60, (j) 1.80, (k) 2.00, (l) 2.20, (m) 2.40, (n) 2.50. $[M\text{-}\mathbf{H}]_0$ = 3.00 mM.

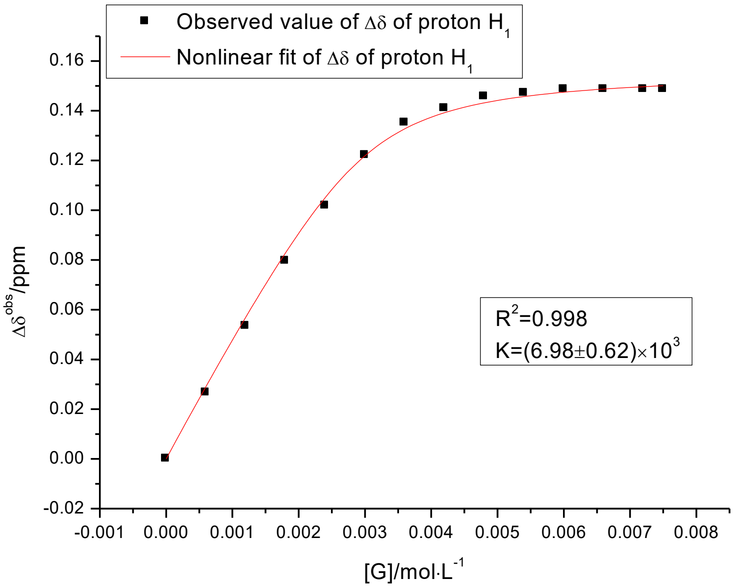

Figure S48. Plots of $\Delta\delta_{obs}$ (ppm) for the H_1 of *M*-**H** vs *S*-**G1** concentration in $CDCl_3$

at 298 K.

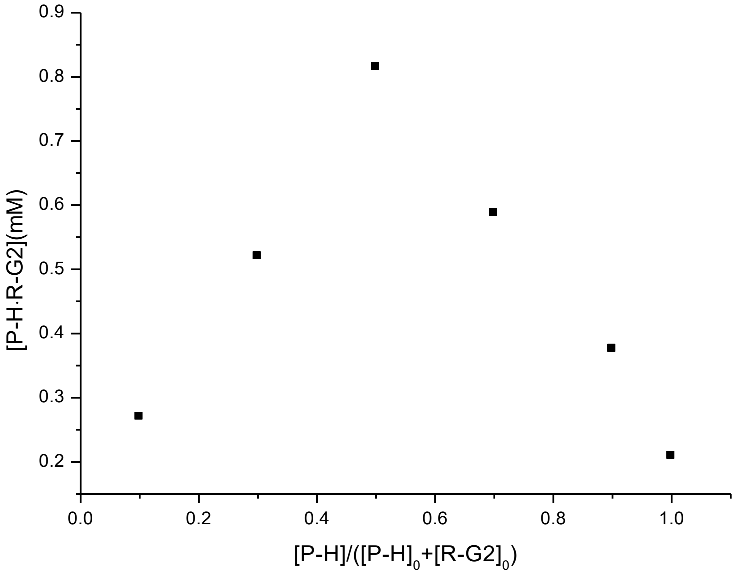

Figure S49. Job plot for the complexation of *P*-**H** and *R*-**G2** in CD_2Cl_2 at 298 K.

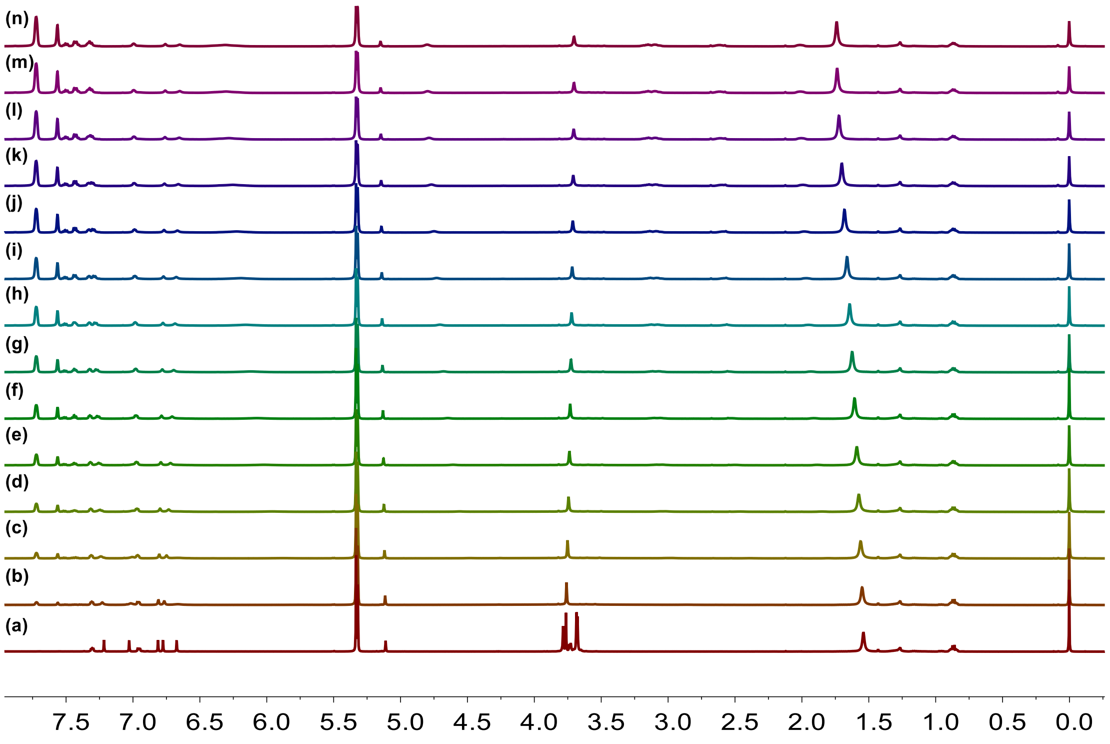

Figure S50. ^{1}H NMR spectra (400 MHz, 298K, CD_2Cl_2) of *P*-**H** with different equivalents of *R*-**G2**: (a) 0.00, (b) 0.20, (c) 0.40, (d) 0.60, (e) 0.80, (f) 1.00, (g) 1.20, (h)

1.40, (i)1.60, (j) 1.80, (k) 2.00, (l) 2.20, (m) 2.40, (n) 2.50. [*P*-**H**]$_0$ = 3.00 mM.

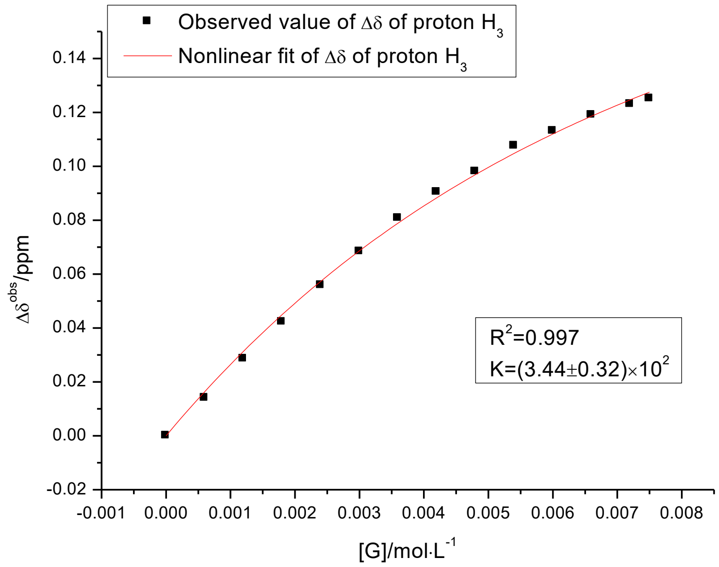

Figure S51. Plots of $\Delta\delta_{obs}$ (ppm) for the H_3 of *P*-**H** vs *R*-**G2** concentration in CD_2Cl_2 at 298 K.

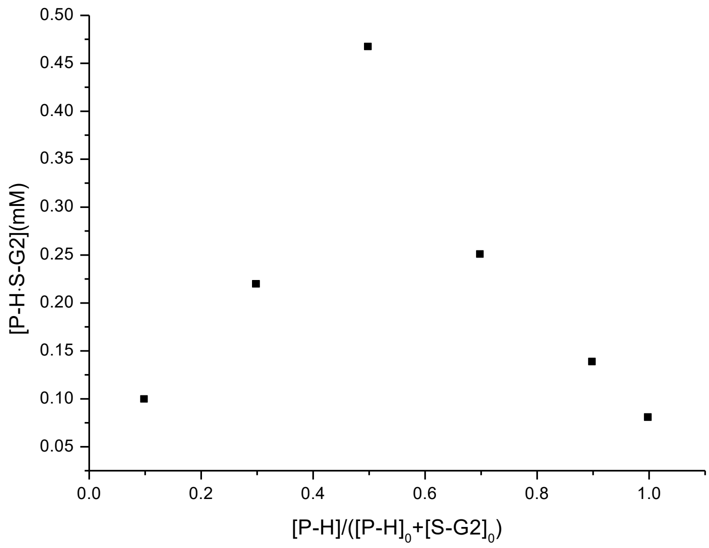

Figure S52. Job plot for the complexation of *P*-**H** and *S*-**G2** in CD_2Cl_2 at 298 K.

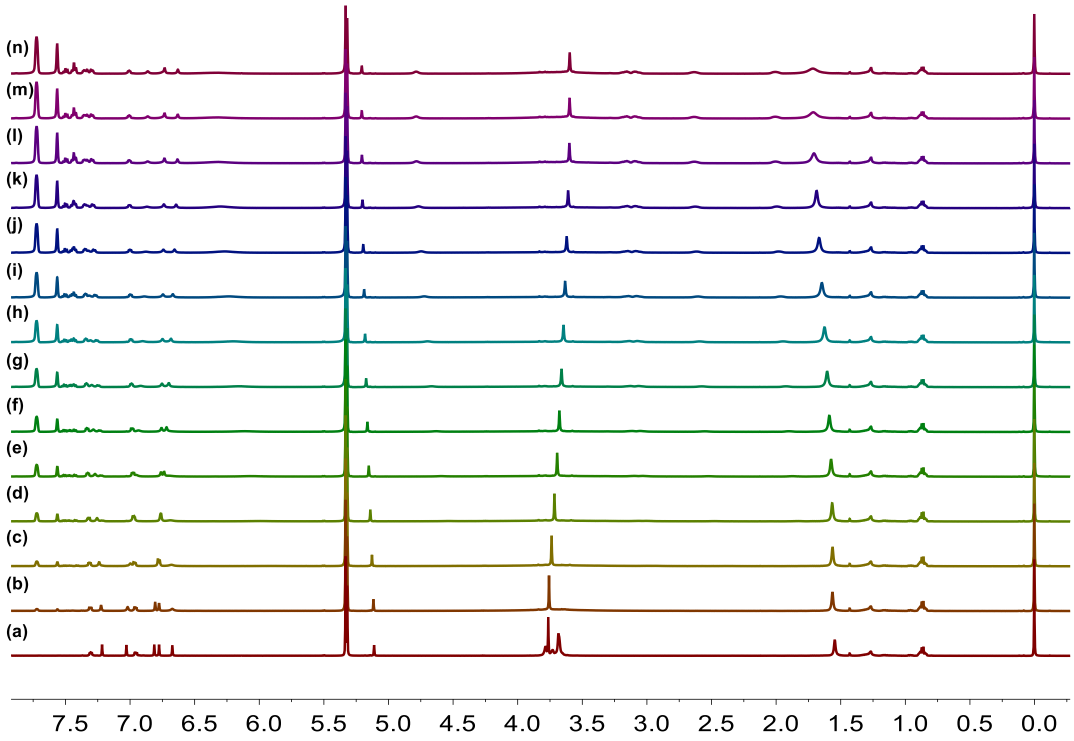

Figure S53. ^{1}H NMR spectra (400 MHz, 298K, CD_2Cl_2) of *P*-**H** with different equivalents of *S*-**G2**: (a) 0.00, (b) 0.20, (c) 0.40, (d) 0.60, (e) 0.80, (f) 1.00, (g) 1.20, (h) 1.40, (i)1.60, (j) 1.80, (k) 2.00, (l) 2.20, (m) 2.40, (n) 2.50. $[P\text{-}\mathbf{H}]_0$ = 3.00 mM.

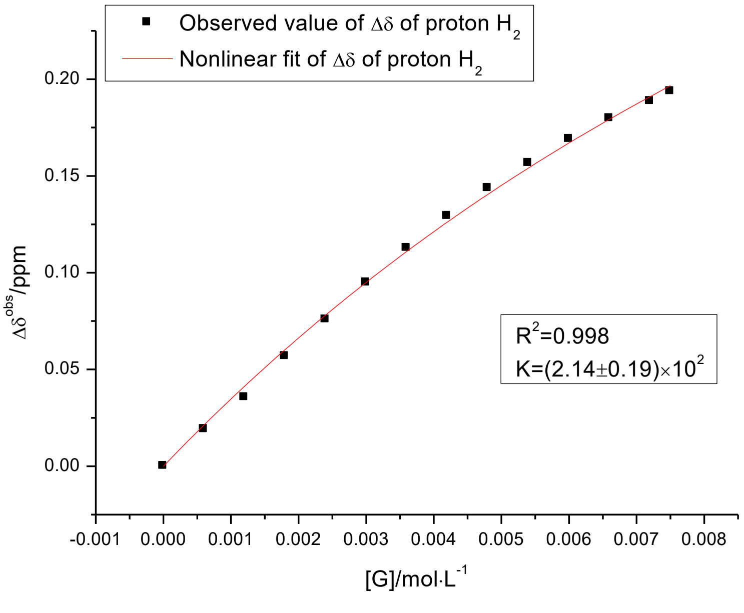

Figure S54. Plots of $\Delta\delta_{obs}$ (ppm) for the H_2 of *P*-**H** vs *S*-**G2** concentration in CD_2Cl_2 at 298 K.

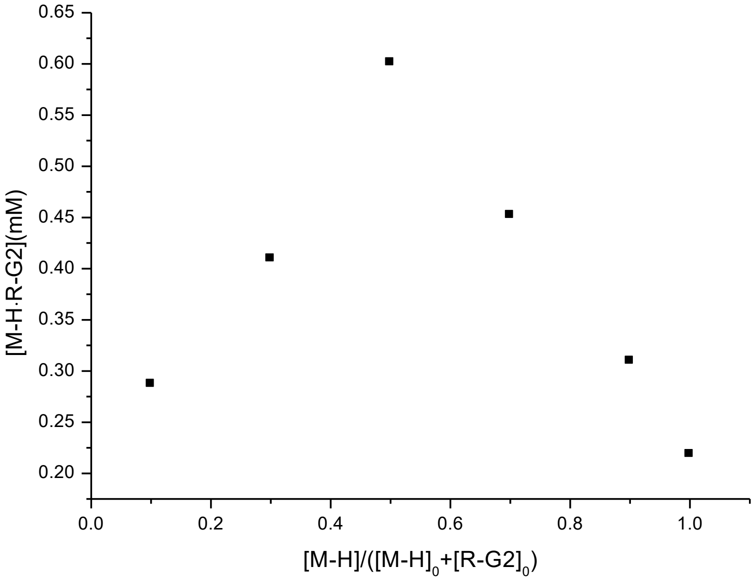

Figure S55. Job plot for the complexation of *M*-**H** and *R*-**G2** in CD_2Cl_2 at 298 K.

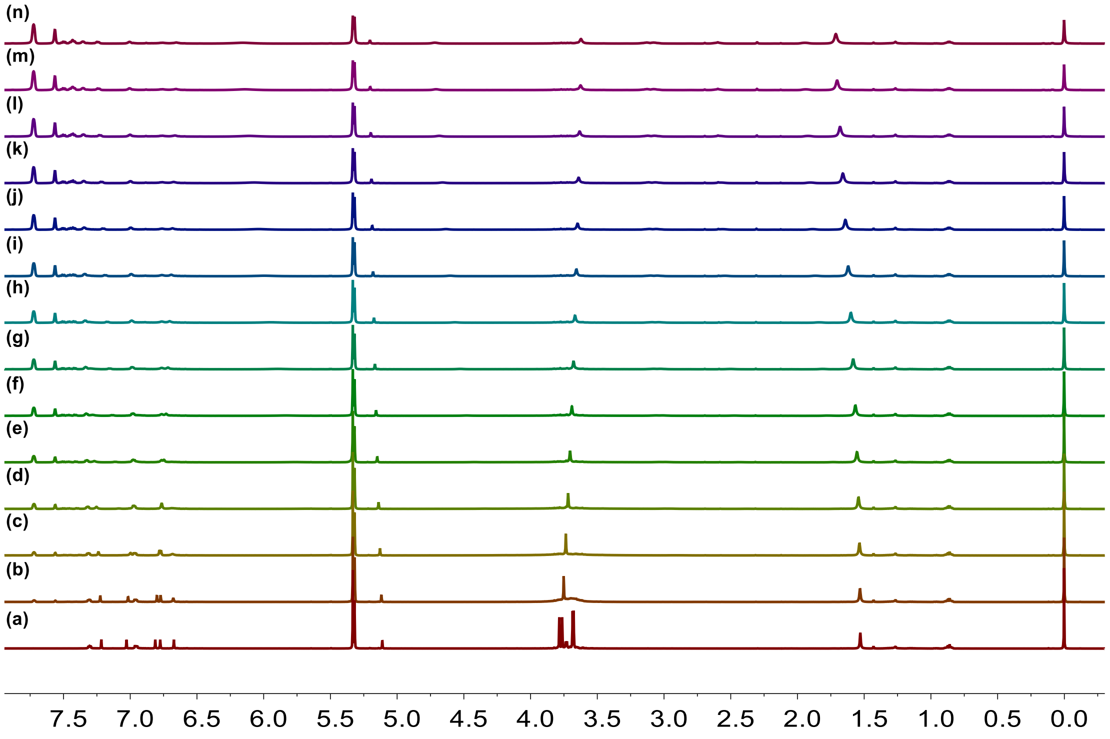

Figure S56. ^{1}H NMR spectra (400 MHz, 298K, CD_2Cl_2) of *M*-**H** with different equivalents of *R*-**G2**: (a) 0.00, (b) 0.20, (c) 0.40, (d) 0.60, (e) 0.80, (f) 1.00, (g) 1.20, (h) 1.40, (i)1.60, (j) 1.80, (k) 2.00, (l) 2.20, (m) 2.40, (n) 2.50. [*M*-**H**]$_0$ = 3.00 mM.

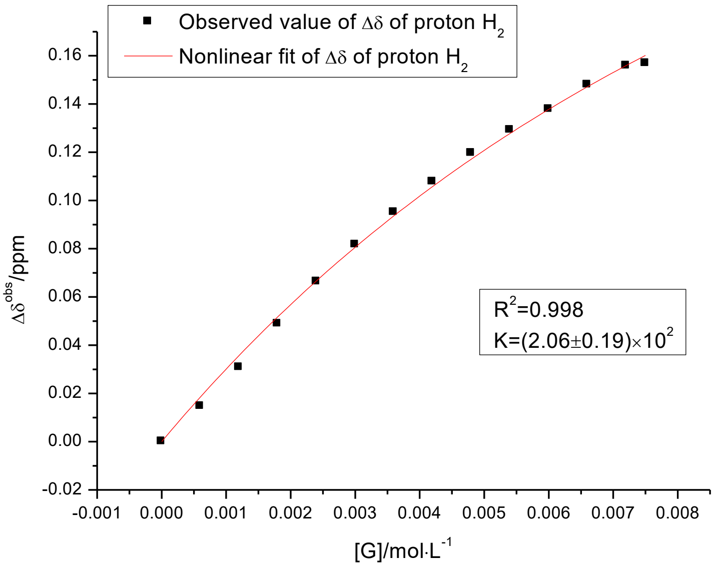

Figure S57. Plots of $\Delta\delta_{obs}$ (ppm) for the H_2 of *M*-**H** vs *R*-**G2** concentration in CD_2Cl_2 at 298 K.

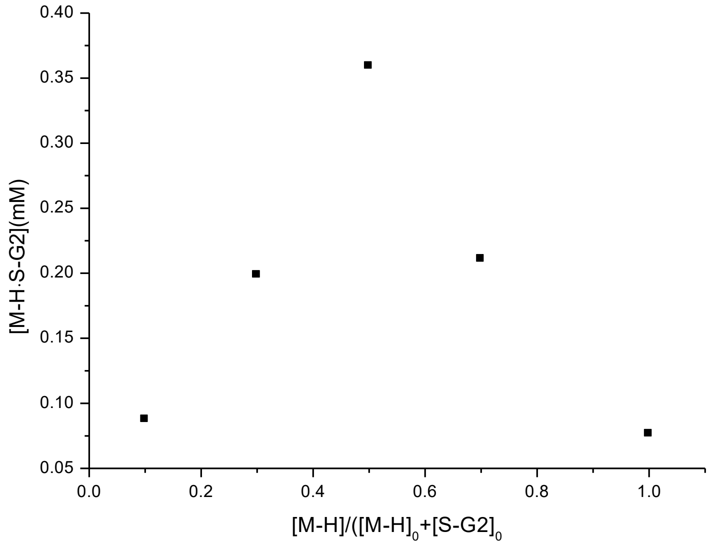

Figure S58. Job plot for the complexation of *M*-**H** and *S*-**G2** in CD_2Cl_2 at 298 K.

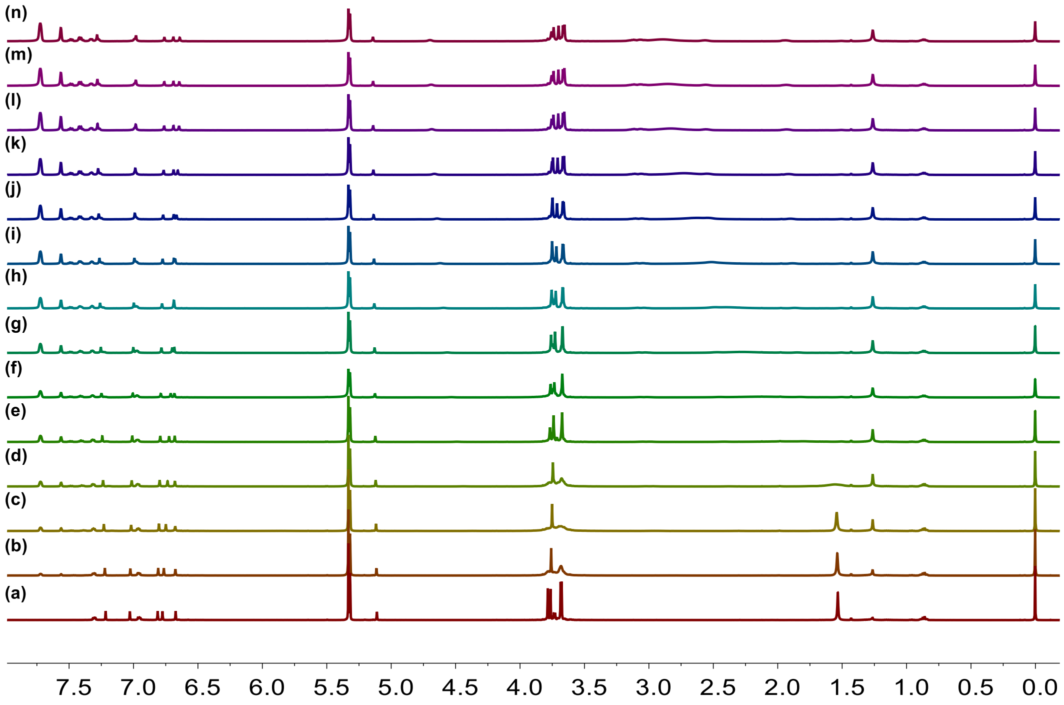

Figure S59. ^{1}H NMR spectra (400 MHz, 298K, CD_2Cl_2) of *M*-**H** with different equivalents of *S*-**G2**: (a) 0.00, (b) 0.20, (c) 0.40, (d) 0.60, (e) 0.80, (f) 1.00, (g) 1.20, (h) 1.40, (i)1.60, (j) 1.80, (k) 2.00, (l) 2.20, (m) 2.40, (n) 2.50. $[M\text{-}\mathbf{H}]_0$ = 3.00 mM.

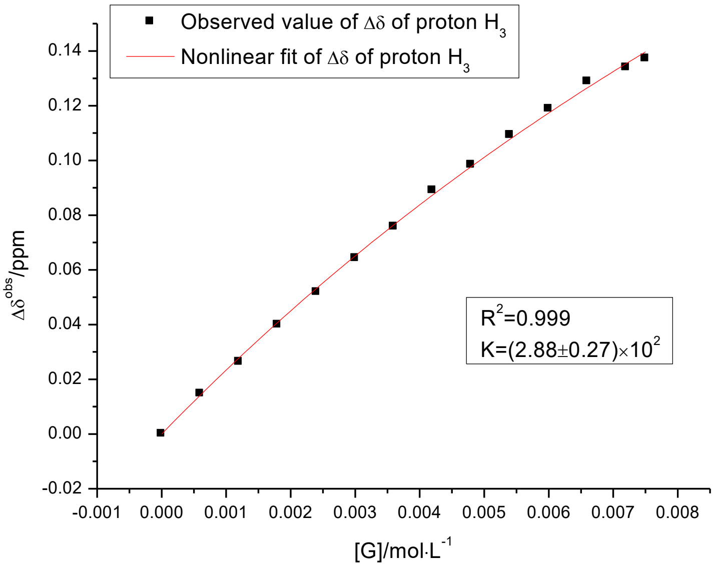

Figure S60. Plots of $\Delta\delta_{obs}$ (ppm) for the H_3 of *M*-**H** vs *S*-**G2** concentration in CD_2Cl_2 at 298 K.

6. Crystal Structures

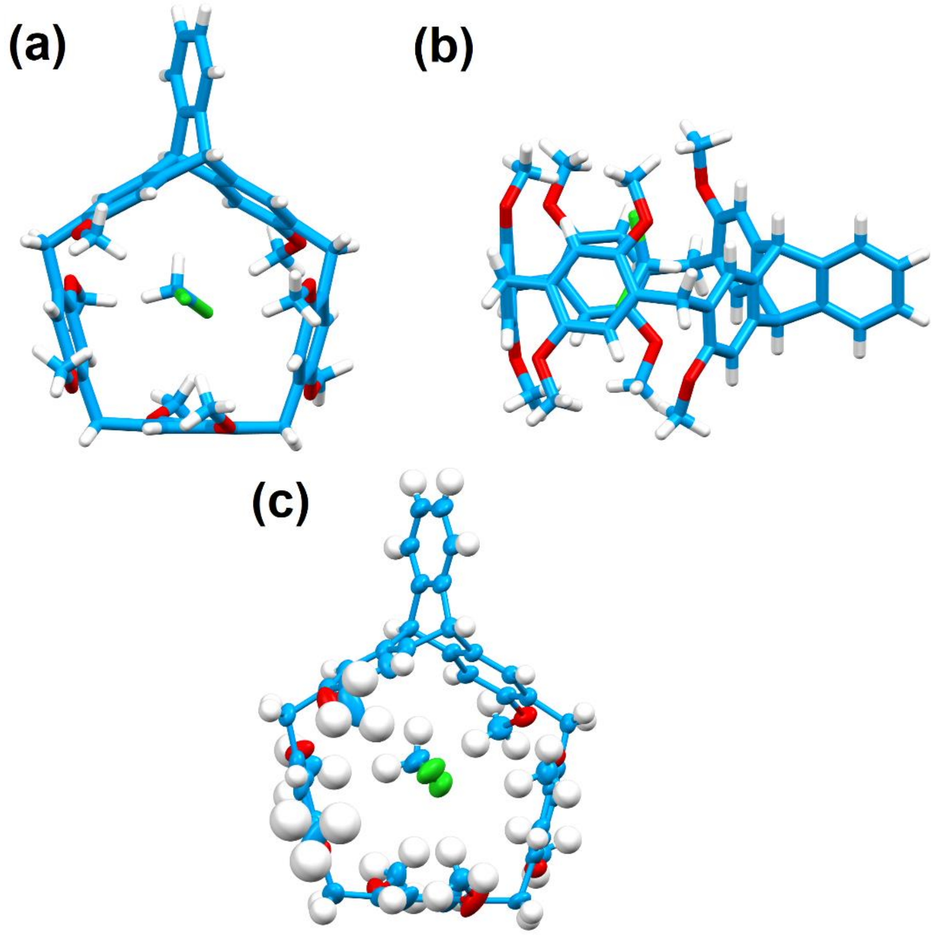

Figure S61. (a) top view, (b) side view, (c) ORTEP view (the thermal ellipsoids are displayed at 30% probability) of *P*-**H**.

Table S4. Crystal Data and Structure Refinement for *P*-**H** (CCDC 2041644).

Empirical formula	$C_{51}H_{50}O_8Cl_2$
Formula weight	861
Temperature	169.99(14) K
Crystal system	Monoclinic
Space group	$P2_1$
Unit cell dimensions	a = 11.5661(2) Å α= 90°<br>b = 20.5779(2) Å β= 117.985(2)°<br>c = 12.3105(10) Å γ = 90°

Volume	2587.37(7) Å^3
Z	2
Density (calculated)	2.734 g/cm^3
Absorption coefficient	2.723 mm^{-1}
F(000)	2105.0
Crystal size	0.15 x 0.1 x 0.08 mm^3
Radiation	CuKα (λ = 1.54184)
Theta range for data collection	8.134 to 150.88°
Index ranges	-14<=h<=13, -25<=k<=22, -14<=l<=15
Reflections collected	23915
Independent reflections	8717 [R_{int} = 0.0210, R_{sigma} =0.0185]
Data / restraints / parameters	8717/91/586
Goodness-of-fit on F^2	1.088
Final R indices [I>2sigma(I)]	R_1 = 0.0524, wR_2 = 0.1471
R indices (all data)	R_1 = 0.0563, wR_2 = 0.1527
Largest diff. peak and hole	0.19/-0.20 e Å^{-3}

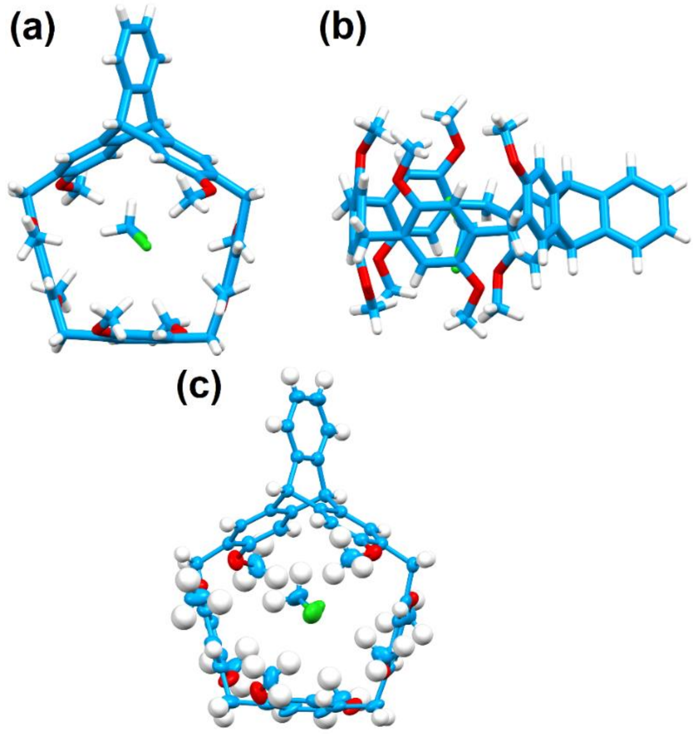

Figure S62. (a) top view, (b) side view, (c) ORTEP view (the thermal ellipsoids are displayed at 30% probability) of *M*-**H**.

Table S5. Crystal Data and Structure Refinement for *M*-**H** (CCDC 2041645).

Empirical formula	$C_{51}H_{50}O_8Cl_2$
Formula weight	861
Temperature	169.99(12) K
Crystal system	Monoclinic
Space group	$P2_1$
Unit cell dimensions	a = 11.5764(2) Å α= 90°<br>b = 20.3868(2) Å β= 117.321(2)°<br>c = 12.2437(10) Å γ = 90°
Volume	2567.25(8) $Å^3$
Z	2
Density (calculated)	4.012 g/cm^3
Absorption coefficient	24.816 mm^{-1}
F(000)	3079.0
Crystal size	0.15 x 0.1 x 0.08 mm^3
Radiation	CuKα (λ = 1.54184)
Theta range for data collection	8.128 to 151.18°
Index ranges	-14<=h<=14, -25<=k<=25, -15<=l<=13
Reflections collected	50060
Independent reflections	10288 [R_{int} = 0.0604, R_{sigma} =0.0410]
Data / restraints / parameters	10288/32/596
Goodness-of-fit on F^2	1.050
Final R indices [I>2sigma(I)]	R_1 = 0.0590, wR_2 = 0.1599
R indices (all data)	R_1 = 0.0641, wR_2 = 0.1661
Largest diff. peak and hole	0.36/-0.20 e $Å^{-3}$

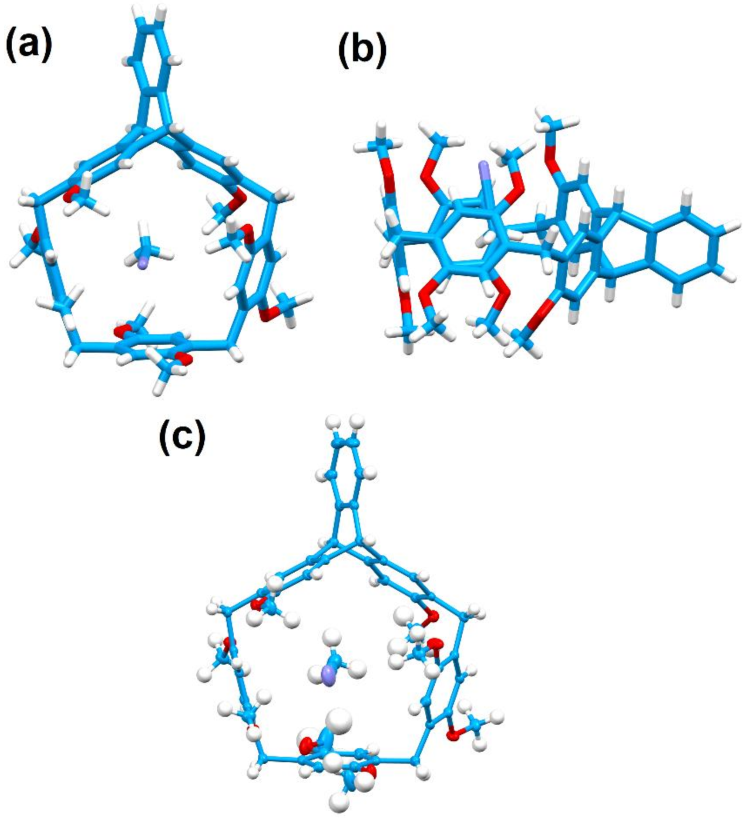

Figure S63. (a) top view, (b) side view, (c) ORTEP view (the thermal ellipsoids are displayed at 30% probability) of *P*-**H**.

Table S6. Crystal Data and Structure Refinement for *P*-**H** (CCDC 2046031).

Empirical formula	$C_{52}H_{51}O_8N$
Formula weight	817.93
Temperature	169.98(13) K
Crystal system	Orthorhombic
Space group	$P2_12_12_1$
Unit cell dimensions	a = 9.3923(10) Å α= 90°<br>b = 21.0240(10) Å β= 90°<br>c = 22.2936(10) Å γ = 90°
Volume	4402.18(6) $Å^3$
Z	4
Density (calculated)	1.234 g/cm^3
Absorption coefficient	0.665 mm^{-1}
F(000)	1736.0

Crystal size	0.26 x 0.2 x 0.12 mm^3
Radiation	CuKα (λ = 1.54184)
Theta range for data collection	5.778 to 150.98°
Index ranges	-11<=h<=11, -26<=k<=26, -21<=l<=27
Reflections collected	41387
Independent reflections	8764 [R_{int} = 0.0310, R_{sigma} =0.0201]
Data / restraints / parameters	8764/0/559
Goodness-of-fit on F^2	1.041
Final R indices [I>2sigma(I)]	R_1 = 0.0331, wR_2 = 0.0885
R indices (all data)	R_1 = 0.0353, wR_2 = 0.0912
Largest diff. peak and hole	0.30/-0.23 e $Å^{-3}$

7. DFT Calculations for the Complexation Between H and G1-G2

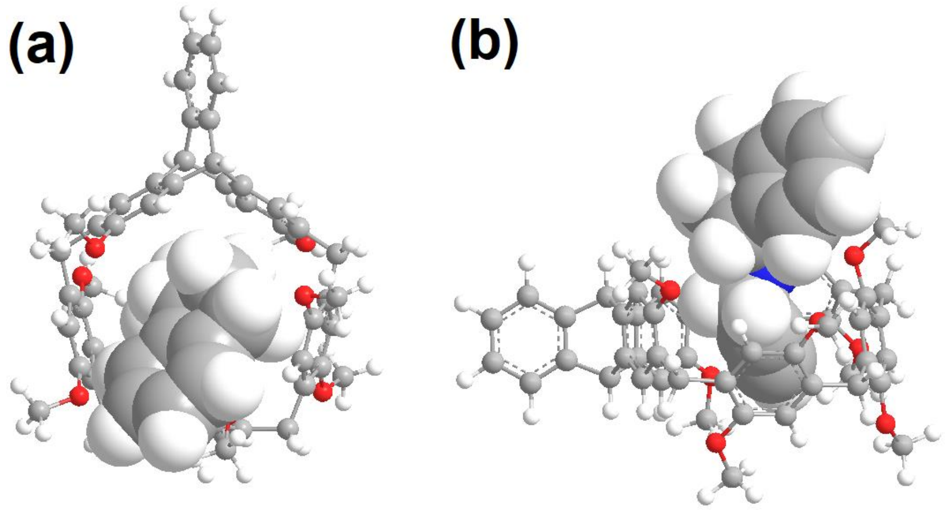

Figure S64. Calculated structure of the complex *P*-**H**·*R*-**G1** (a) top view, (b) side view at the B3LYP/6-31G level.

The atomic coordinates of *P*-**H**·*R*-**G1**.

C	4.1961	-3.6494	2.8132
C	4.27806	-3.53302	4.21378
C	3.24571	-2.91804	4.94562
C	2.13037	-2.41857	4.25795
C	2.06029	-2.52577	2.85217
C	3.08243	-3.14609	2.11614
C	0.87911	-1.75674	4.81827
C	-0.12195	-1.81253	3.62018
C	0.75173	-1.95192	2.33789
N	0.95219	-0.5627	1.64014
C	0.30398	-0.57104	0.24363
C	0.43071	0.68835	-0.47613
C	0.49729	1.70279	-1.14065
H	4.99492	-4.14694	2.2657
H	5.14184	-3.93824	4.73856
H	3.30776	-2.85061	6.03102
H	3.02256	-3.25413	1.03211
H	0.48459	-2.27676	5.70224
H	1.08418	-0.71763	5.12671
H	-0.80261	-0.95187	3.57727

H	-0.74172	-2.71632	3.7036
H	0.28077	-2.58802	1.5786
H	0.51078	0.18708	2.21836
H	1.97179	-0.32871	1.57901
H	-0.74912	-0.83174	0.40375
H	0.77226	-1.39302	-0.31167
H	0.5846	2.61483	-1.68876
O	-1.11252	-4.3039	0.10562
O	-0.80706	-1.52547	-4.45267
O	3.53175	-3.38372	-1.42022
O	3.75288	0.43398	1.58732
C	-2.04701	-3.31684	-0.30807
C	-2.78141	-2.61823	0.67355
C	-3.61616	-1.57091	0.28316
C	-3.74991	-1.24592	-1.08857
C	-3.01922	-1.94549	-2.04993
C	-2.13828	-2.99096	-1.67538
C	-4.68509	-0.04163	-1.32666
C	-6.01689	-0.37109	-0.62932
C	-7.28591	-0.38153	-1.21527
C	-8.40818	-0.69851	-0.42351
C	-1.27587	-3.69719	-2.72232
C	0.1069	-3.06399	-2.87752
C	0.3153	-1.9552	-3.73584
C	1.57333	-1.34138	-3.83199
C	2.67348	-1.80849	-3.08211
C	2.46709	-2.92042	-2.2451
C	1.20662	-3.53355	-2.1445
C	4.00761	-1.06441	-3.12676
C	4.04859	0.07376	-2.10609
C	4.01207	-0.21207	-0.73542
C	3.94595	0.81003	0.21738
O	-2.55678	4.51867	-0.57586
O	-0.20095	1.77492	3.28724
O	2.24827	5.36163	-0.25575
O	4.05471	1.6783	-3.86708
C	-2.99485	3.25297	-0.15743
C	-3.64383	2.34698	-1.01975
C	-4.05322	1.10872	-0.51894
C	-3.86562	0.78802	0.84194
C	-3.19916	1.6864	1.68127
C	-2.73121	2.92367	1.19246
C	-4.39403	-0.61582	1.20623

C	-5.86071	-0.67446	0.74303
C	-6.97306	-0.98993	1.52841
C	-8.25316	-0.99976	0.9382
C	-1.93616	3.87315	2.08721
C	-0.42356	3.78747	1.86357
C	0.37643	2.80424	2.47027
C	1.74909	2.71258	2.21727
C	2.3807	3.54688	1.28748
C	1.59008	4.54799	0.67565
C	0.22333	4.67763	0.97917
C	3.83278	3.29562	0.88502
C	3.95696	2.1686	-0.14668
C	4.01716	2.46027	-1.52593
C	4.04645	1.43605	-2.49002
C	-1.68305	-5.5385	0.67474
C	-0.19603	2.05567	4.73878
C	4.13053	-4.67149	-1.81937
C	4.97256	-0.00948	2.30274
C	-2.9554	5.00053	-1.89293
C	-0.66776	-0.40833	-5.37875
C	1.56494	6.54725	-0.76536
C	4.1475	3.05137	-4.34849
H	-2.67283	-2.88679	1.72415
H	-3.08744	-1.67763	-3.10227
H	-4.8069	0.19726	-2.38869
H	-7.4089	-0.14782	-2.27257
H	-9.39963	-0.70936	-0.87252
H	-1.14861	-4.75009	-2.44037
H	-1.79519	-3.66737	-3.68774
H	1.72394	-0.47799	-4.47491
H	1.0673	-4.36644	-1.45847
H	4.82336	-1.7673	-2.91125
H	4.17134	-0.65585	-4.12935
H	4.00842	-1.25318	-0.41487
H	-3.81314	2.59183	-2.06532
H	-3.01792	1.43426	2.72535
H	-4.27309	-0.85461	2.2688
H	-6.85511	-1.22405	2.58601
H	-9.12499	-1.24246	1.54329
H	-2.25847	4.9059	1.9017
H	-2.17034	3.64018	3.13429
H	2.3329	1.93501	2.70238
H	-0.38326	5.43229	0.48916

H	4.26002	4.2136	0.4665
H	4.41236	3.03804	1.78203
H	4.0046	3.50471	-1.82785
H	-0.82704	-6.14964	0.97304
H	-2.30988	-5.31912	1.55018
H	-2.28219	-6.06796	-0.07909
H	-0.65964	1.18935	5.21939
H	0.83283	2.18978	5.10095
H	-0.78013	2.95972	4.95304
H	4.9029	-4.88446	-1.07473
H	3.37681	-5.47068	-1.8167
H	4.58031	-4.59296	-2.81835
H	4.68979	-0.0956	3.35772
H	5.30143	-0.98578	1.92327
H	5.77188	0.73487	2.17567
H	-2.59338	6.03067	-1.94527
H	-2.49342	4.40477	-2.69461
H	-4.04839	4.98627	-2.00729
H	0.06774	-0.63185	-6.16422
H	-1.65726	-0.28178	-5.82542
H	-0.37627	0.51048	-4.84921
H	2.30759	7.06469	-1.37794
H	0.69737	6.27451	-1.38382
H	1.23994	7.19921	0.05771
H	4.19074	2.96894	-5.43748
H	3.26452	3.63782	-4.05394
H	5.05762	3.54243	-3.97626

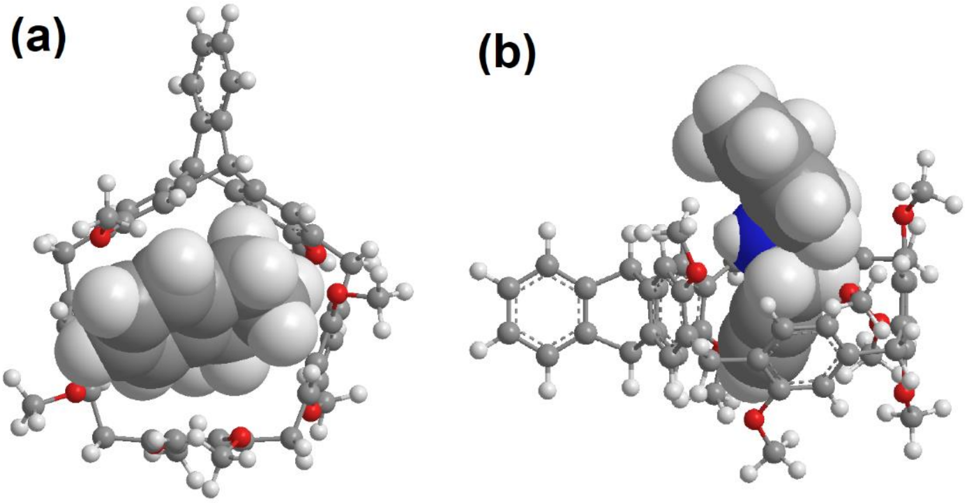

Figure S65. Calculated structure of the complex *P*-**H**·*S*-**G1** (a) top view, (b) side view at the B3LYP/6-31G level.

The atomic coordinates of *P*-**H**·*S*-**G1**.

O	1.4946	-0.86282	3.31067
O	-3.45657	-2.49719	3.43173
O	1.01592	-5.7317	2.90584
O	1.04231	-5.85797	-2.46158
C	0.46699	-0.1725	2.72381
C	0.66255	0.71561	1.64809
C	-0.42734	1.42208	1.14259
C	-1.69551	1.27903	1.71988
C	-1.8859	0.35854	2.74575
C	-0.82035	-0.40167	3.24978
C	-2.74697	2.19976	1.08616
C	-2.17743	3.61908	1.15284
C	-2.77966	4.73042	1.7293
C	-2.11728	5.96486	1.69333
C	-1.03162	-1.4523	4.3318
C	-1.152	-2.85915	3.7602
C	-2.38913	-3.34013	3.27824
C	-2.47828	-4.59569	2.67313
C	-1.35244	-5.42201	2.5302
C	-0.13662	-4.96334	3.04872
C	-0.04599	-3.70234	3.65144
C	-1.45083	-6.73008	1.7547
C	-1.42416	-6.48098	0.25349

C	-0.21012	-6.37994	-0.4314
C	-0.15517	-6.03214	-1.78832
O	-4.74186	0.6271	-3.27078
O	0.42709	-0.73564	-4.30339
O	-3.87185	-4.23944	-4.30598
O	-3.78375	-6.40534	0.22621
C	-3.70366	1.03899	-2.47405
C	-3.87407	1.4386	-1.1378
C	-2.76441	1.83173	-0.39779
C	-1.49683	1.89735	-0.99077
C	-1.33382	1.47672	-2.3061
C	-2.42311	1.01649	-3.06054
C	-0.41376	2.40392	-0.03056
C	-0.91875	3.73226	0.53893
C	-0.2612	4.95546	0.50279
C	-0.86814	6.07691	1.08407
C	-2.21782	0.45042	-4.45757
C	-1.97038	-1.05226	-4.4169
C	-0.6798	-1.58493	-4.31192
C	-0.4811	-2.96535	-4.18102
C	-1.5484	-3.86424	-4.15241
C	-2.84697	-3.33136	-4.29842
C	-3.0444	-1.95269	-4.42068
C	-1.31887	-5.35748	-3.96526
C	-1.33822	-5.78604	-2.50339
C	-2.55618	-5.92872	-1.82638
C	-2.61134	-6.27033	-0.47339
C	1.0654	-0.59983	-5.58106
C	-5.18199	-3.78538	-4.62237
C	2.80741	-0.31924	3.21576
C	-6.07221	0.85022	-2.82188
C	-4.74685	-2.96896	3.07408
C	1.37198	-6.47365	4.07745
C	2.15021	-6.66176	-2.05485
C	-5.00758	-6.3689	-0.49406
H	1.66043	0.9018	1.25959
H	-2.87395	0.21054	3.17322
H	-3.72795	2.11337	1.56036
H	-3.75359	4.6447	2.20527
H	-2.58136	6.83797	2.14323
H	-0.19131	-1.42475	5.03128
H	-1.93954	-1.20922	4.89031
H	-3.41934	-4.9472	2.26835

H	0.91958	-3.36194	4.01224
H	-0.61344	-7.37781	2.02423
H	-2.37607	-7.24736	2.02174
H	0.70566	-6.55861	0.12373
H	-4.85625	1.4349	-0.67628
H	-0.35038	1.48743	-2.77137
H	0.56817	2.49408	-0.50413
H	0.71286	5.04435	0.02736
H	-0.36118	7.03736	1.0588
H	-3.09849	0.65445	-5.07216
H	-1.36562	0.95326	-4.92196
H	0.53482	-3.34972	-4.12173
H	-4.04529	-1.54648	-4.49787
H	-2.0949	-5.90615	-4.50671
H	-0.3549	-5.62816	-4.40209
H	-3.46523	-5.74483	-2.38519
H	1.91418	0.07273	-5.4376
H	1.42133	-1.56932	-5.95031
H	0.37684	-0.16854	-6.31753
H	-5.80562	-4.67994	-4.6633
H	-5.57015	-3.10564	-3.85288
H	-5.20491	-3.28058	-5.59571
H	3.41901	-0.89116	3.91526
H	3.22605	-0.43605	2.20993
H	2.81546	0.74048	3.49803
H	-6.71993	0.57484	-3.65598
H	-6.31961	0.2249	-1.95338
H	-6.23587	1.90389	-2.56569
H	-5.44237	-2.17211	3.3441
H	-4.81929	-3.16725	1.9967
H	-5.00772	-3.88074	3.62586
H	2.28874	-7.01742	3.83715
H	1.55332	-5.80676	4.92957
H	0.58337	-7.18751	4.34703
H	2.8919	-6.57985	-2.85222
H	2.59423	-6.3034	-1.11752
H	1.85165	-7.70958	-1.93669
H	-5.79065	-6.57989	0.23636
H	-5.18395	-5.38194	-0.94192
H	-5.03043	-7.1315	-1.28244
C	3.89865	-3.81216	1.89604
C	5.00327	-2.96233	1.77968
C	5.07886	-2.02974	0.74063

C	4.03891	-1.96166	-0.1854
C	2.93745	-2.82593	-0.07363
C	2.85879	-3.75627	0.96514
C	3.90762	-1.06238	-1.3942
C	2.81657	-1.76622	-2.23548
C	2.00528	-2.64186	-1.24948
N	0.67884	-1.945	-0.88369
C	-0.29415	-2.7808	-0.06025
C	-1.62825	-2.22027	-0.16102
C	-2.72964	-1.74827	-0.2891
H	3.84656	-4.53514	2.70383
H	5.81585	-3.03317	2.49746
H	5.9448	-1.3793	0.64903
H	2.02425	-4.44234	1.0653
H	3.60356	-0.0474	-1.10004
H	4.84479	-0.95401	-1.94939
H	3.29167	-2.44669	-2.94987
H	2.18462	-1.0904	-2.81782
H	1.67693	-3.58309	-1.69903
H	0.84353	-1.06324	-0.37521
H	0.20544	-1.69476	-1.76424
H	0.07517	-2.79513	0.96819
H	-0.25851	-3.79671	-0.46231
H	-3.68507	-1.28911	-0.40124

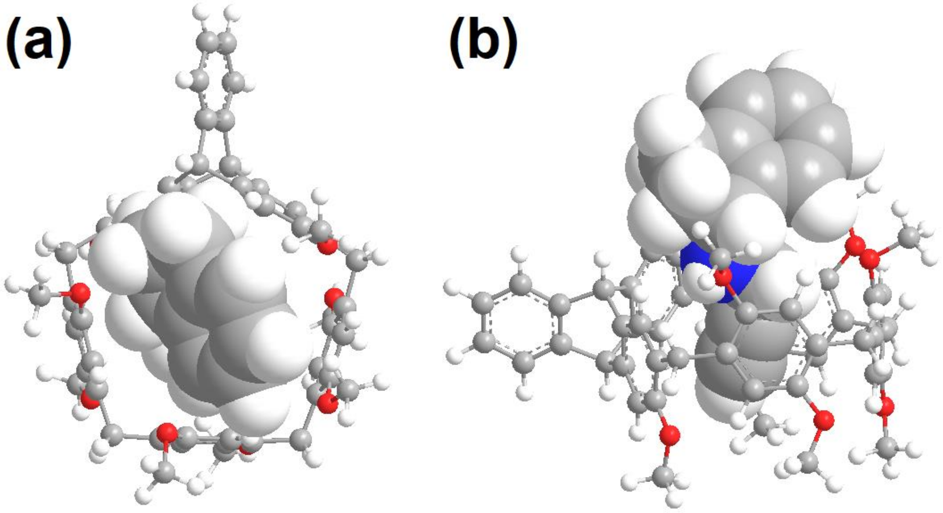

Figure S66. Calculated structure of the complex *M*-**H**·*R*-**G1** (a) top view, (b) side view at the B3LYP/6-31G level.

The atomic coordinates of *M*-**H**·*R*-**G1**.

C	3.78202	-3.21894	-1.73644
C	4.69731	-2.21983	-2.08638
C	4.66173	-0.96899	-1.46393
C	3.68788	-0.72192	-0.49642
C	2.75619	-1.71782	-0.16702
C	2.80561	-2.98001	-0.76597
C	3.48539	0.52036	0.34384
C	2.52868	0.03994	1.46427
C	1.83837	-1.2376	0.93093
N	0.42034	-0.9068	0.43342
C	-0.36348	-2.07891	-0.13582
C	-1.73886	-1.68531	-0.37531
C	-2.87354	-1.33497	-0.57854
H	3.84383	-4.19554	-2.20743
H	5.45969	-2.4264	-2.83224
H	5.39822	-0.21062	-1.71657
H	2.12484	-3.77291	-0.46436
H	4.42431	0.90881	0.75143
H	3.046	1.33629	-0.24585
H	1.81662	0.80429	1.79141
H	3.10644	-0.24682	2.34779
H	1.65948	-1.96443	1.72264
H	-0.11662	-0.52946	1.22903
H	0.42973	-0.15197	-0.27432
H	-0.29193	-2.89028	0.59269
H	0.138	-2.38478	-1.05635
H	-3.86593	-1.01591	-0.80151
O	0.7456	0.85185	-3.78553
O	-4.70573	-0.00269	3.58611
O	-3.93579	-4.83049	3.2972
O	0.48321	-1.51097	3.75205
O	-4.29989	-5.70974	-1.71315
O	0.75735	-5.54824	0.56156
O	-4.28035	-0.8917	-3.90604
O	0.23454	-4.0928	-4.30126
C	-0.20623	1.28294	-2.90144
C	0.10491	1.83203	-1.64126
C	-0.92882	2.29103	-0.82113
C	-0.79737	2.86188	0.59541
C	-1.74665	2.02142	1.46464
C	-1.43803	1.25567	2.58848

C	-2.43133	0.53975	3.27978
C	-3.76503	0.65484	2.84037
C	-4.08229	1.40475	1.69709
C	-3.07128	2.06113	1.00795
C	-3.24018	2.87145	-0.27593
C	-2.25289	2.25473	-1.2708
C	-2.55143	1.66743	-2.49693
C	-1.54609	1.14217	-3.31794
C	-1.39951	4.26835	0.53006
C	-0.77228	5.45766	0.87898
C	-1.47164	6.66482	0.74642
C	-2.78121	6.67088	0.26796
C	-3.41264	5.47012	-0.08346
C	-2.7188	4.27379	0.04791
C	-2.08309	-0.35833	4.45913
C	-1.90324	-1.81131	4.03513
C	-3.0088	-2.66801	3.92555
C	-2.8813	-3.96845	3.43134
C	-1.62403	-4.46304	3.02394
C	-0.52065	-3.62639	3.18636
C	-0.65157	-2.32324	3.67848
C	-1.47959	-5.83656	2.38646
C	-1.63213	-5.78508	0.87108
C	-2.90527	-5.81999	0.28217
C	-3.07424	-5.70257	-1.09983
C	-1.95791	-5.55476	-1.95058
C	-0.69516	-5.54099	-1.36061
C	-0.52739	-5.64715	0.02358
C	-2.1245	-5.355	-3.45041
C	-2.07518	-3.88326	-3.83354
C	-3.2213	-3.08781	-3.75482
C	-3.18051	-1.71498	-4.0172
C	-1.97189	-1.10005	-4.38447
C	-0.83146	-1.90265	-4.49621
C	-0.87041	-3.2736	-4.22371
C	-1.88669	0.40486	-4.60455
C	1.25038	-1.69601	4.94992
C	1.39535	-3.5937	-4.94699
C	-5.44794	-6.00052	-0.92982
C	-5.56542	-1.49577	-3.87786
C	-6.08097	0.21671	3.29799
C	-5.20057	-4.44382	3.81778
C	2.09285	1.26401	-3.59999

C	1.52008	-6.75832	0.47899
H	1.13949	1.96932	-1.33859
H	0.23291	2.8663	0.96477
H	-0.41956	1.2231	2.97423
H	-5.1061	1.47764	1.34648
H	-4.26875	2.87481	-0.64533
H	-3.58243	1.59346	-2.83319
H	0.24927	5.45475	1.25213
H	-0.98897	7.59926	1.01815
H	-3.3169	7.61037	0.1663
H	-4.43389	5.47686	-0.45656
H	-1.16339	0.00839	4.92193
H	-2.87671	-0.29762	5.20924
H	-3.97968	-2.27866	4.20193
H	0.46382	-3.99338	2.90617
H	-2.23258	-6.51099	2.8026
H	-0.49612	-6.23998	2.64073
H	-3.76367	-5.91429	0.93598
H	0.17946	-5.42051	-1.99416
H	-1.32896	-5.89057	-3.97466
H	-3.07919	-5.7817	-3.76661
H	-4.14685	-3.56369	-3.45084
H	0.09761	-1.42661	-4.78491
H	-1.12522	0.61624	-5.3604
H	-2.84461	0.77115	-4.98225
H	2.10234	-1.01457	4.8874
H	0.6541	-1.45491	5.83809
H	1.61023	-2.72869	5.0317
H	2.08021	-4.44036	-5.0295
H	1.16458	-3.21494	-5.95067
H	1.87753	-2.79783	-4.36317
H	-6.2867	-6.02947	-1.62765
H	-5.35624	-6.97335	-0.43073
H	-5.62971	-5.22439	-0.17455
H	-6.28531	-0.67787	-3.95012
H	-5.70469	-2.18058	-4.72357
H	-5.73995	-2.04602	-2.94285
H	-6.63655	-0.31519	4.07213
H	-6.33291	1.28328	3.33883
H	-6.35679	-0.18475	2.31415
H	-5.85794	-5.30285	3.67342
H	-5.13757	-4.20988	4.88766
H	-5.60978	-3.57779	3.28145

H	2.6218	0.97376	-4.50953
H	2.16295	2.35088	-3.46916
H	2.5587	0.75877	-2.74435
H	2.50248	-6.53931	0.90412
H	1.04314	-7.56287	1.05259
H	1.63483	-7.08502	-0.56175

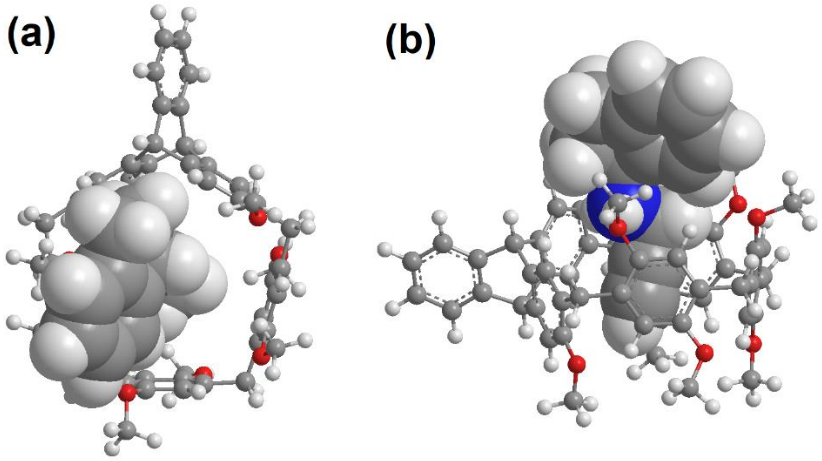

Figure S67. Calculated structure of the complex *M*-**H**·*S*-**G1** (a) top view, (b) side view at the B3LYP/6-31G level.

The atomic coordinates of *M*-**H**·*S*-**G1**.

C	3.77028	-4.2172	1.1757
C	4.90195	-3.59669	1.71276
C	5.05155	-2.21102	1.63069
C	4.04911	-1.45583	1.02483
C	2.90319	-2.07659	0.50289
C	2.76855	-3.46699	0.55352
C	4.02471	0.03596	0.78921
C	2.5457	0.31129	0.44313
C	1.99592	-1.02555	-0.11687
N	0.52022	-1.15257	0.23719
C	-0.28748	-2.1565	-0.57295
C	-1.69304	-2.04444	-0.2301
C	-2.85565	-1.92879	0.0634
H	3.66378	-5.29575	1.23524
H	5.6723	-4.19578	2.18967
H	5.9372	-1.72805	2.03486
H	1.91987	-3.99701	0.13395

H	4.36548	0.61996	1.65028
H	4.67796	0.30327	-0.05211
H	2.41368	1.1414	-0.25426
H	1.99741	0.56047	1.36091
H	2.0245	-1.04825	-1.21314
H	0.42915	-1.35914	1.26223
H	0.07173	-0.23773	0.07768
H	0.09829	-3.15384	-0.35778
H	-0.1158	-1.9291	-1.6302
H	-3.87958	-1.81333	0.33624
O	0.35188	0.8174	-3.65811
O	-4.76527	-0.65405	3.70727
O	-3.72519	-5.40184	3.71949
O	0.31328	-1.65942	3.04424
O	-4.47676	-5.83066	-1.62824
O	0.7014	-6.12742	0.34061
O	-4.62061	-1.13118	-3.85578
O	-0.04802	-4.24081	-4.30072
C	-0.64807	1.20845	-2.79613
C	-0.37774	1.80452	-1.54949
C	-1.43655	2.16384	-0.71862
C	-1.33834	2.74282	0.69507
C	-2.15975	1.78446	1.56836
C	-1.71557	1.01757	2.64117
C	-2.5839	0.15825	3.32996
C	-3.94167	0.1376	2.95158
C	-4.39308	0.88181	1.84961
C	-3.49225	1.67527	1.14972
C	-3.78792	2.47602	-0.11864
C	-2.75993	1.98326	-1.1433
C	-3.015	1.37794	-2.36759
C	-1.97033	0.95785	-3.20634
C	-2.10019	4.06927	0.65844
C	-1.60762	5.32038	1.00806
C	-2.44563	6.43901	0.90816
C	-3.75861	6.29701	0.46077
C	-4.25425	5.03457	0.10839
C	-3.423	3.92578	0.20802
C	-2.07582	-0.75715	4.43368
C	-1.87176	-2.19002	3.95379
C	-2.87801	-3.15212	4.12233
C	-2.74854	-4.44874	3.61323
C	-1.57771	-4.83807	2.9315

C	-0.56496	-3.89068	2.79804
C	-0.7119	-2.59316	3.28722
C	-1.43882	-6.21172	2.29247
C	-1.6752	-6.14658	0.79088
C	-2.98028	-6.0717	0.28091
C	-3.22035	-5.9149	-1.08607
C	-2.14671	-5.81873	-1.99791
C	-0.85449	-5.91644	-1.48678
C	-0.6151	-6.08068	-0.11862
C	-2.38572	-5.56408	-3.47867
C	-2.35837	-4.08005	-3.81314
C	-3.51835	-3.30707	-3.71075
C	-3.50601	-1.92987	-3.95169
C	-2.30849	-1.28599	-4.3066
C	-1.15201	-2.06323	-4.43577
C	-1.16584	-3.44186	-4.19523
C	-2.25975	0.22199	-4.50609
C	1.4449	-1.79952	3.92233
C	1.09269	-3.71499	-4.95929
C	-5.59412	-6.07982	-0.78876
C	-5.8831	-1.75665	-3.68903
C	-6.16814	-0.57529	3.49292
C	-4.8791	-5.11289	4.49702
C	1.59166	1.51192	-3.60933
C	1.34481	-7.38677	0.1157
H	0.64241	2.03276	-1.25642
H	-0.30685	2.85855	1.04156
H	-0.67781	1.07259	2.96402
H	-5.43104	0.84083	1.537
H	-4.81852	2.36408	-0.4644
H	-4.03696	1.20391	-2.69238
H	-0.58396	5.43234	1.35788
H	-2.06865	7.42054	1.18116
H	-4.40263	7.16848	0.38434
H	-5.27842	4.92577	-0.24027
H	-1.13258	-0.35523	4.81597
H	-2.78689	-0.76364	5.26528
H	-3.78504	-2.85179	4.63033
H	0.35211	-4.16946	2.28512
H	-2.15125	-6.90083	2.75148
H	-0.43262	-6.59363	2.48392
H	-3.80419	-6.1176	0.98424
H	-0.0109	-5.84263	-2.16746

H	-1.61566	-6.07868	-4.05914
H	-3.35448	-5.98065	-3.76539
H	-4.43312	-3.80717	-3.41543
H	-0.23507	-1.56361	-4.7268
H	-1.48684	0.46371	-5.24056
H	-3.21849	0.5653	-4.9031
H	2.14531	-1.00095	3.66867
H	1.12542	-1.69351	4.96432
H	1.93167	-2.7704	3.78406
H	1.79573	-4.54482	-5.05469
H	0.84087	-3.33298	-5.95662
H	1.56699	-2.90981	-4.37978
H	-6.47013	-6.05197	-1.43935
H	-5.52565	-7.06593	-0.31263
H	-5.69556	-5.31162	-0.01034
H	-6.62385	-0.956	-3.73588
H	-6.08125	-2.48202	-4.48824
H	-5.96061	-2.26441	-2.71793
H	-6.6249	-1.19476	4.2668
H	-6.53124	0.45489	3.59143
H	-6.45094	-0.96538	2.50603
H	-5.47859	-6.02464	4.4905
H	-4.61256	-4.85964	5.53058
H	-5.46247	-4.29076	4.06216
H	2.13022	1.23604	-4.51808
H	1.4407	2.59759	-3.5874
H	2.19697	1.21703	-2.74
H	2.35723	-7.30145	0.5179
H	0.8139	-8.19574	0.63273
H	1.39763	-7.62199	-0.95424

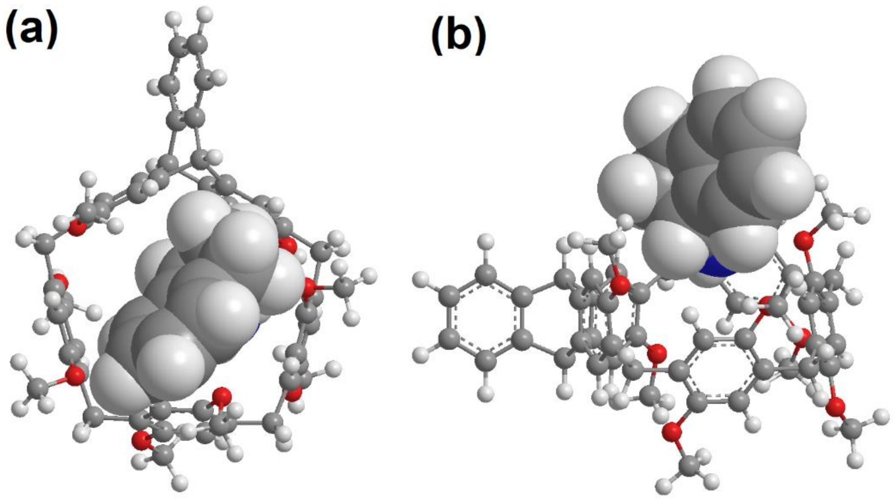

Figure S68. Calculated structure of the complex *P*-**H**·*R*-**G2** (a) top view, (b) side view at the B3LYP/6-31G level.

The atomic coordinates of *P*-**H**·*R*-**G2**.

C	-3.79719	-4.8758	-2.57939
C	-3.44021	-5.11034	-3.91007
C	-2.51246	-4.28397	-4.5654
C	-1.96243	-3.21998	-3.86227
C	-2.33846	-2.9749	-2.51959
C	-3.24294	-3.80194	-1.8655
C	-0.94049	-2.2243	-4.33251
C	-0.60513	-1.37064	-3.08848
C	-1.60686	-1.77533	-1.97717
N	-2.60925	-0.63502	-1.74917
H	-4.51039	-5.53563	-2.08476
H	-3.88257	-5.95041	-4.45001
H	-2.2336	-4.48127	-5.59924
H	-3.51817	-3.64368	-0.81396
H	-0.03824	-2.72489	-4.73386
H	-1.33331	-1.60561	-5.16169
H	-0.63543	-0.28514	-3.321
H	0.43093	-1.56383	-2.74537
H	-1.09104	-1.99399	-1.00386
H	-2.25414	0.05951	-1.07033
H	-2.78607	-0.11651	-2.64105

H	-3.54004	-0.98245	-1.40598
O	0.04005	-4.93563	0.07329
O	-0.54542	-2.39521	4.65784
O	-4.48103	-3.8417	1.04854
O	-5.21238	-0.04572	-1.45569
C	0.79079	-3.79544	0.28013
C	1.37621	-3.03906	-0.7495
C	2.07484	-1.89232	-0.39264
C	2.23336	-1.52721	0.9681
C	1.63102	-2.27667	1.97277
C	0.88141	-3.41403	1.63979
C	3.0727	-0.26965	1.16892
C	4.37667	-0.50793	0.41328
C	5.65453	-0.42326	0.94207
C	6.7545	-0.66751	0.09819
C	0.14961	-4.187	2.70107
C	-1.25373	-3.66087	2.82363
C	-1.59908	-2.71641	3.82942
C	-2.88286	-2.17205	3.92028
C	-3.87538	-2.56831	3.00942
C	-3.53239	-3.50927	2.02634
C	-2.24315	-4.05436	1.92829
C	-5.25307	-1.968	3.07195
C	-5.34915	-0.78331	2.1523
C	-5.33465	-0.95458	0.77342
C	-5.37778	0.1636	-0.07332
O	0.80513	4.162	0.35041
O	-1.79252	1.54814	-3.48137
O	-3.83932	4.49164	0.72365
O	-5.44332	0.60377	4.03108
C	1.24289	2.90034	-0.00555
C	1.94133	2.04142	0.86364
C	2.35076	0.81292	0.3708
C	2.11261	0.45229	-0.98464
C	1.383	1.29271	-1.81299
C	0.90563	2.52181	-1.32428
C	2.7119	-0.8995	-1.36328
C	4.18203	-0.83789	-0.9574
C	5.26736	-1.07608	-1.78514
C	6.56403	-0.98735	-1.24417
C	0.05371	3.40942	-2.18883
C	-1.39741	3.27928	-1.81304
C	-2.27154	2.41475	-2.48641

C	-3.61978	2.27271	-2.11862
C	-4.11459	2.94453	-1.00832
C	-3.24315	3.84656	-0.33379
C	-1.91406	4.01031	-0.7295
C	-5.50184	2.66831	-0.50341
C	-5.47224	1.47487	0.41366
C	-5.49502	1.65309	1.80723
C	-5.43754	0.5456	2.65772
C	0.07885	-5.54432	-1.24091
C	-2.00893	2.03275	-4.83546
C	-5.05862	-5.16314	1.22415
C	-6.3686	-0.64381	-2.10505
C	1.3542	4.74636	1.55834
C	-0.82757	-1.58858	5.82831
C	-3.08044	5.52997	1.39563
C	-5.6691	1.89254	4.65686
H	1.30948	-3.35132	-1.78552
H	1.72314	-1.98466	3.02093
H	3.22442	-0.01123	2.23262
H	5.81628	-0.17577	1.98884
H	7.76349	-0.60468	0.50542
H	0.13742	-5.27184	2.44762
H	0.67848	-4.11697	3.67853
H	-3.12911	-1.43538	4.68212
H	-2.00887	-4.77343	1.13799
H	-6.014	-2.72904	2.78349
H	-5.5087	-1.67458	4.11609
H	-5.28158	-1.9665	0.35579
H	2.15495	2.32602	1.88905
H	1.18303	1.01821	-2.848
H	2.57103	-1.16072	-2.42676
H	5.13291	-1.3276	-2.83423
H	7.42415	-1.17292	-1.88707
H	0.38564	4.46878	-2.07407
H	0.19989	3.16586	-3.26304
H	-4.27927	1.61518	-2.69075
H	-1.24007	4.68026	-0.18773
H	-5.90559	3.56451	0.02233
H	-6.19895	2.48308	-1.34998
H	-5.54164	2.66565	2.20607
H	-0.4708	-6.47972	-1.06979
H	-0.44161	-4.90919	-1.964
H	1.11013	-5.75372	-1.54041

H	-1.54717	1.25335	-5.44992
H	-3.08056	2.12358	-5.03333
H	-1.50432	2.99444	-4.97239
H	-5.79925	-5.21055	0.41942
H	-4.2864	-5.92848	1.10657
H	-5.53664	-5.23677	2.20553
H	-6.07036	-0.67526	-3.15766
H	-6.54872	-1.64589	-1.7054
H	-7.24393	-0.00216	-1.96111
H	0.96747	5.77228	1.50927
H	0.97476	4.2187	2.43732
H	2.44875	4.74609	1.52381
H	-1.56965	-2.08151	6.46368
H	0.15325	-1.56354	6.32222
H	-1.14478	-0.58588	5.53004
H	-3.80594	5.88993	2.13735
H	-2.19777	5.10168	1.87882
H	-2.81707	6.32543	0.69139
H	-5.67652	1.62197	5.72189
H	-4.84552	2.57449	4.42916
H	-6.63787	2.30063	4.35303

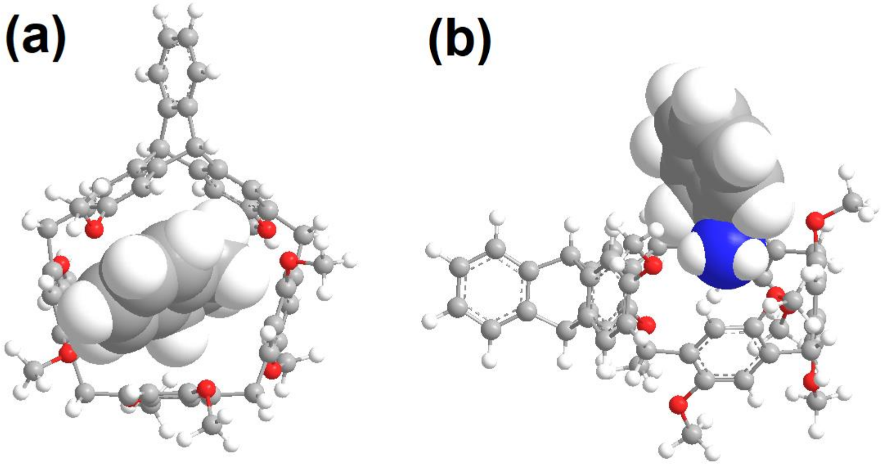

Figure S69. Calculated structure of the complex *P*-**H**·*S*-**G2** (a) top view, (b) side view at the B3LYP/6-31G level.

The atomic coordinates of *P*-**H**·*S*-**G2**.

C	2.104	-4.1314	-0.0991
C	3.6262	-4.0796	-0.0991
C	4.3424	-2.7355	-0.0991

C	3.5365	-1.4432	-0.0991
C	2.0143	-1.495	-0.0991
C	1.2981	-2.8391	-0.0991
C	3.9576	0.0204	-0.0991
C	2.6957	0.8732	-0.0991
C	1.4947	-0.0634	-0.0991
N	0.0311	0.3577	-0.0991
H	1.5867	-5.1022	-0.0991
H	4.2082	-5.013	-0.0991
H	5.4417	-2.6981	-0.0991
H	0.1988	-2.8765	-0.0991
H	4.561	0.2394	0.8102
H	4.5761	0.2449	-0.9967
H	2.6739	1.5147	-1.0083
H	2.6734	1.5308	0.7986
H	0.8906	0.1104	-1.0175
H	-0.0317	1.4008	-0.0991
H	-0.4392	-0.0191	-0.9528
H	-0.4397	-0.0194	0.7542
O	1.6376	0.4185	4.9233
O	-3.6525	-1.5124	3.7246
O	1.0218	-5.3487	4.3316
O	2.6875	-6.643	-0.6757
C	0.78	0.4822	3.6663
C	1.3106	1.3557	2.4451
C	0.2739	2.1039	1.5248
C	-1.2886	2.0125	1.8192
C	-1.8023	1.1597	3.0413
C	-0.7849	0.3769	3.9806
C	-2.2576	2.885	0.6656
C	-1.6576	4.518	0.6729
C	-2.503	5.8241	0.937
C	-1.7625	7.2326	0.9182
C	-1.3641	-0.6448	5.2542
C	-1.4922	-2.2723	4.681
C	-2.7126	-2.7103	3.7608
C	-2.7594	-4.1568	3.1286
C	-1.598	-5.2063	3.3945
C	-0.3913	-4.7806	4.3278
C	-0.3494	-3.3252	4.9718
C	-1.6455	-6.7623	2.6359
C	-1.001	-6.6912	1.0317
C	0.5625	-6.6171	0.7926

C	1.1757	-6.4596	-0.6624
O	-2.6575	0.5336	-4.9619
O	2.4758	-0.6334	-3.9629
O	-1.6837	-4.9762	-5.1621
O	-3.467	-6.4508	-0.2147
C	-2.1924	0.9639	-3.2772
C	-2.7871	1.562	-1.9263
C	-1.8119	2.2249	-0.882
C	-0.2496	2.3213	-1.1759
C	0.3265	1.7422	-2.5241
C	-0.6267	1.0503	-3.5932
C	0.6493	3.0596	0.1194
C	-0.0893	4.611	0.3923
C	0.6383	6.0111	0.3664
C	-0.2208	7.3255	0.6249
C	0.0288	0.3201	-5.0209
C	0.3012	-1.3652	-4.7393
C	1.5617	-1.8499	-3.9003
C	1.7378	-3.375	-3.5295
C	0.6693	-4.4583	-3.9831
C	-0.5767	-3.9821	-4.837
C	-0.7489	-2.4459	-5.2178
C	0.8565	-6.1128	-3.5072
C	0.2206	-6.3783	-1.9201
C	-1.3416	-6.4821	-1.6835
C	-1.9551	-6.6342	-0.228
C	2.5341	1.6493	4.9561
C	3.9834	-0.8083	-3.8361
C	1.2336	-6.8555	4.3967
C	3.5996	-5.4238	-0.6413
C	-2.6015	-0.9874	-4.9089
C	-3.084	-0.0995	3.7252
C	-3.1189	-4.4838	-5.2932
C	-4.379	-7.67	-0.2492
H	2.3896	1.4357	2.2462
H	-2.8814	1.1051	3.2478
H	-3.3493	2.8301	0.8753
H	-3.5841	5.7621	1.1305
H	-2.3357	8.1511	1.1126
H	-0.653	-0.6088	6.1097
H	-2.3656	-0.29	5.5859
H	-3.6109	-4.4419	2.4933
H	0.4878	-3.048	5.6294

H	-1.043	-7.4787	3.2381
H	-2.7057	-7.0958	2.5774
H	1.2406	-6.6763	1.6567
H	-3.8673	1.5126	-1.7247
H	1.4047	1.8178	-2.7287
H	1.7401	3.1364	-0.0884
H	1.7192	6.0773	0.1732
H	0.2694	8.3099	0.5981
H	-0.6893	0.4432	-5.8623
H	0.9933	0.8123	-5.2781
H	2.6155	-3.692	-2.9472
H	-1.6118	-2.1315	-5.8233
H	0.3135	-6.7624	-4.2296
H	1.942	-6.3586	-3.4996
H	-2.0185	-6.448	-2.5499
H	3.1701	1.6214	5.8691
H	3.1837	1.6587	4.0524
H	1.9041	2.5667	4.9709
H	4.4799	0.1854	-3.9052
H	4.221	-1.2736	-2.8534
H	4.3509	-1.4643	-4.6567
H	2.3242	-7.078	4.3899
H	0.7802	-7.2533	5.3321
H	0.7516	-7.3361	3.5161
H	4.6623	-5.7543	-0.6571
H	3.3972	-4.7845	-1.5296
H	3.4059	-4.842	0.2876
H	-2.872	-1.4041	-5.9049
H	-1.5712	-1.311	-4.6394
H	-3.3198	-1.3586	-4.1441
H	-3.9184	0.6367	3.6963
H	-2.4832	0.0577	4.6488
H	-2.4355	0.0398	2.8314
H	-3.786	-5.3423	-5.5314
H	-3.4405	-4.0198	-4.334
H	-3.1791	-3.7297	-6.1097
H	-5.4417	-7.3395	-0.2334
H	-4.1856	-8.2512	-1.1784
H	-4.1764	-8.3099	0.6387

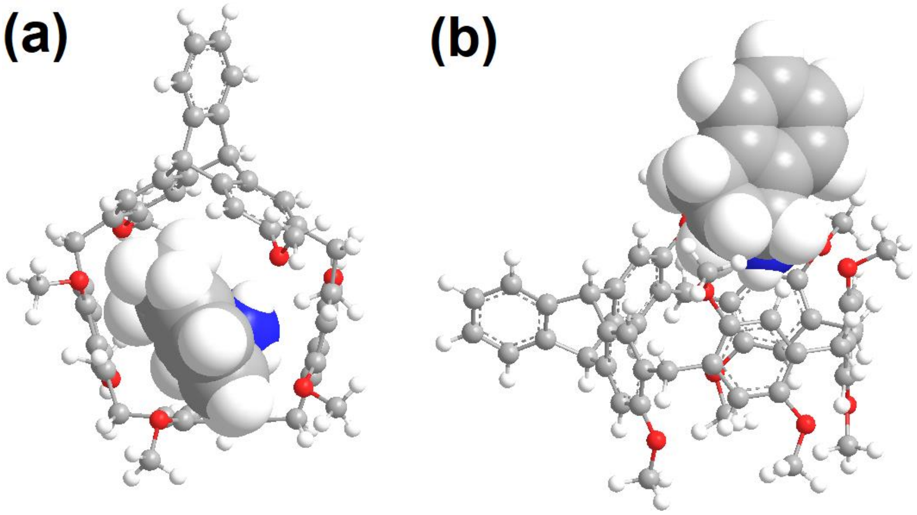

Figure S70. Calculated structure of the complex *M*-**H**·*R*-**G2** (a) top view, (b) side view at the B3LYP/6-31G level.

The atomic coordinates of *M*-**H**·*R*-**G2**.

C	2.2475	-4.9284	-0.329
C	3.7696	-4.8766	-0.329
C	4.4858	-3.5325	-0.329
C	3.6799	-2.2402	-0.329
C	2.1578	-2.292	-0.329
C	1.4416	-3.6361	-0.329
C	4.101	-0.7766	-0.329
C	2.8392	0.0762	-0.329
C	1.6382	-0.8604	-0.329
N	0.1746	-0.4393	-0.329
H	1.7302	-5.8992	-0.329
H	4.3517	-5.81	-0.329
H	5.5852	-3.4951	-0.329
H	0.3422	-3.6735	-0.329
H	4.7044	-0.5576	0.5803
H	4.7196	-0.5521	-1.2266
H	2.8174	0.7177	-1.2382
H	2.8168	0.7338	0.5687
H	1.0341	-0.6866	0.5895
H	0.1117	0.6038	-0.329
H	-0.2958	-0.8161	-1.1827
H	-0.2962	-0.8165	0.5243
O	-3.6716	-0.2203	4.4598

O	1.6142	-0.2324	-4.4279
O	1.0423	-5.6678	-4.7288
O	-4.0772	-2.0081	-4.029
O	2.1862	-6.6868	0.8509
O	-3.8159	-6.9262	-1.395
O	2.1122	-1.7846	3.9859
O	-2.8127	-5.7285	4.3313
C	-2.7041	0.4724	3.4477
C	-3.207	1.2149	2.1204
C	-2.1679	1.8949	1.1726
C	-2.5006	2.6808	-0.3507
C	-1.4979	1.8598	-1.5289
C	-1.9841	1.0863	-2.824
C	-0.9429	0.3589	-3.7796
C	0.6169	0.4451	-3.4167
C	1.1171	1.1934	-2.087
C	0.049	1.8935	-1.1488
C	0.3765	2.7045	0.3645
C	-0.6084	1.8852	1.5453
C	-0.128	1.1329	2.8503
C	-1.146	0.3964	3.8079
C	-1.8356	4.2939	-0.1943
C	-2.637	5.6568	-0.3562
C	-1.8453	7.0392	-0.1122
C	-0.3278	7.0563	0.2774
C	0.4736	5.6745	0.4194
C	-0.2943	4.3072	0.1885
C	-1.4622	-0.58	-5.1528
C	-1.514	-2.2748	-4.8116
C	-0.229	-3.1959	-5.012
C	-0.2362	-4.7324	-4.6114
C	-1.5432	-5.4056	-3.9968
C	-2.8489	-4.4951	-3.8368
C	-2.8329	-2.9558	-4.2093
C	-1.5353	-7.0413	-3.4112
C	-1.1636	-7.0997	-1.7197
C	0.3444	-6.9496	-1.2093
C	0.7191	-6.8915	0.3158
C	-0.4306	-7.0273	1.4278
C	-1.9457	-7.1606	0.9152
C	-2.2911	-7.1667	-0.6211
C	-0.0488	-6.9351	3.1243
C	-0.2254	-5.3068	3.7064

C	1.0279	-4.3236	3.6069
C	0.915	-2.8065	4.088
C	-0.4501	-2.2354	4.6748
C	-1.6881	-3.2231	4.7747
C	-1.585	-4.7271	4.2895
C	-0.5917	-0.574	5.1312
C	-4.7364	0.0206	4.5508
C	-5.1694	-2.0512	-3.9564
C	-3.8774	-5.4875	4.4222
C	-4.9082	-6.9693	-1.3224
C	2.6595	0.0918	-4.4772
C	3.2044	-1.7416	3.9133
C	2.0876	-5.3435	-4.7781
C	3.2784	-6.6437	0.7783
H	-4.2794	1.2459	1.8774
H	-3.5796	2.6789	-0.6236
H	-3.0569	1.0523	-3.0648
H	2.1865	1.2197	-1.8309
H	1.4551	2.7201	0.6385
H	0.9437	1.1221	3.0981
H	-3.7031	5.6566	-0.6273
H	-2.3826	7.9928	-0.2218
H	0.1862	8.0129	0.4529
H	1.5437	5.6755	0.6741
H	-2.4809	-0.2444	-5.4499
H	-0.7247	-0.4209	-5.9711
H	0.685	-2.7566	-5.4382
H	-3.7779	-4.9479	-3.46
H	-0.7694	-7.6233	-3.9711
H	-2.5486	-7.4756	-3.5647
H	1.1508	-6.8845	-1.9547
H	-2.7583	-7.2493	1.6512
H	-0.7375	-7.6016	3.6901
H	1.007	-7.2552	3.2714
H	1.9804	-4.6962	3.2019
H	-2.638	-2.8553	5.1897
H	-1.3171	-0.4896	5.9711
H	0.4128	-0.2072	5.4397
H	-5.1945	-0.6137	5.3423
H	-4.8514	1.0936	4.8232
H	-5.2447	-0.1731	3.5798
H	-5.5741	-1.0225	-3.8272
H	-5.5852	-2.4987	-4.8868

H	-5.4586	-2.676	-3.0819
H	-4.4727	-6.428	4.42
H	-4.0533	-4.9401	5.3752
H	-4.188	-4.85	3.5644
H	-5.3578	-6.6451	-2.2876
H	-5.2237	-8.0129	-1.0988
H	-5.2526	-6.2939	-0.5075
H	3.1975	-0.5043	-5.2479
H	2.7004	1.1706	-4.748
H	3.1424	-0.0614	-3.4861
H	3.532	-0.6789	3.8672
H	3.6546	-2.2296	4.8065
H	3.5361	-2.2722	2.9928
H	2.7497	-6.2347	-4.8563
H	2.2326	-4.6948	-5.6707
H	2.3423	-4.772	-3.8576
H	3.7072	-6.3892	1.7734
H	3.6672	-7.6334	0.4495
H	3.5705	-5.8651	0.0385

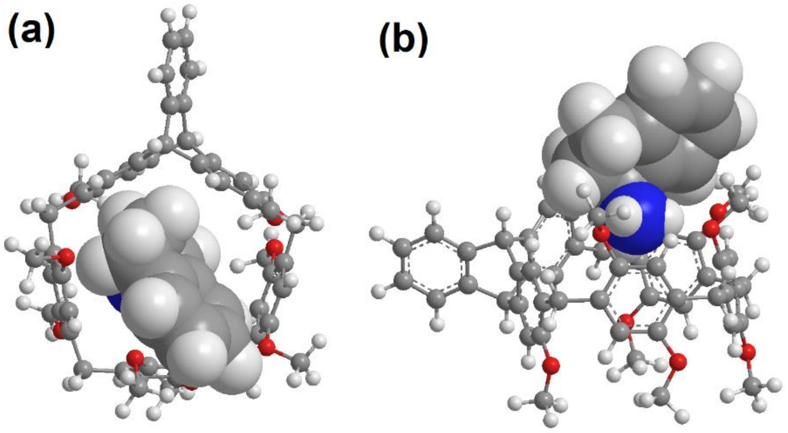

Figure S71. Calculated structure of the complex *M*-**H**·*S*-**G2** (a) top view, (b) side view at the B3LYP/6-31G level.

The atomic coordinates of *M*-**H**·*S*-**G2**.

C	2.55581	-4.22755	-2.72785
C	2.2229	-4.40458	-4.07358
C	1.32355	-3.53716	-4.7153
C	0.77733	-2.49104	-3.98298

C	1.12921	-2.30426	-2.62448
C	2.00533	-3.1719	-1.98451
C	-0.21717	-1.45986	-4.4352
C	-0.56364	-0.64822	-3.16635
C	0.40717	-1.11327	-2.05121
N	1.42411	-0.00091	-1.75874
H	3.2469	-4.91862	-2.24463
H	2.66198	-5.23125	-4.63641
H	1.06315	-3.68971	-5.76145
H	2.26114	-3.0589	-0.92229
H	0.2035	-0.81731	-5.23191
H	-1.11937	-1.92844	-4.87363
H	-1.60993	-0.83529	-2.85211
H	-0.50938	0.44462	-3.35627
H	-0.13291	-1.35967	-1.0979
H	1.06693	0.67325	-1.06073
H	2.34137	-0.37809	-1.4105
H	1.62857	0.54827	-2.62597
O	-1.95198	4.77181	0.45544
O	-1.33656	-4.32073	-0.15971
O	3.18092	-3.34613	0.95013
O	-0.80526	-1.96964	4.53119
O	4.03008	0.52979	-1.38908
O	4.15512	0.96331	4.1224
O	0.68147	2.26197	-3.42138
O	2.68851	5.00334	0.93666
C	-2.40354	3.53285	0.04168
C	-3.13482	2.65365	0.86222
C	-3.55455	1.45258	0.31363
C	-4.31161	0.35309	1.05398
C	-3.48976	-0.91058	0.82167
C	-2.92178	-1.70886	1.80861
C	-2.1848	-2.84574	1.44712
C	-2.07185	-3.17606	0.07578
C	-2.62224	-2.37019	-0.93545
C	-3.30861	-1.22573	-0.54867
C	-3.90787	-0.185	-1.49285
C	-3.29383	1.14028	-1.04965
C	-2.5326	1.99887	-1.82948
C	-2.04485	3.1995	-1.28357
C	-5.6031	0.16744	0.26296
C	-6.89029	0.25447	0.76824
C	-7.97608	0.06263	-0.10697

C	-7.7626	-0.20857	-1.45655
C	-6.45643	-0.29952	-1.97376
C	-5.38503	-0.11284	-1.11522
C	-1.48901	-3.67204	2.49233
C	-0.07983	-3.17618	2.66413
C	0.92154	-3.55254	1.77472
C	2.21752	-3.03473	1.92039
C	2.55554	-2.1386	2.94614
C	1.55079	-1.76002	3.85109
C	0.26013	-2.27745	3.71276
C	3.94163	-1.56586	3.0603
C	4.07743	-0.34852	2.1896
C	4.08937	-0.46629	0.80508
C	4.16954	0.68268	0.00352
C	4.27592	1.97237	0.54294
C	4.27208	2.09635	1.94253
C	4.17768	0.95813	2.74799
C	4.34533	3.19949	-0.32626
C	2.97401	3.51973	-0.8484
C	2.49149	2.90034	-1.99403
C	1.15397	3.08062	-2.38364
C	0.28063	3.93391	-1.69508
C	0.78658	4.6131	-0.5734
C	2.10397	4.41042	-0.15719
C	-1.15975	4.10444	-2.09522
C	-2.51665	5.31892	1.67358
C	-0.53437	-1.21366	5.73756
C	1.93336	6.02823	1.633
C	4.39001	2.2227	4.80231
C	-1.35777	-4.87757	-1.4971
C	0.93502	2.79465	-4.75074
C	3.73192	-4.68351	1.08592
C	5.18942	-0.06356	-2.03723
H	-3.36531	2.90228	1.89332
H	-4.48148	0.57296	2.12358
H	-3.03116	-1.45584	2.86521
H	-2.53889	-2.64344	-1.98124
H	-3.74888	-0.40752	-2.56257
H	-2.31542	1.761	-2.87007
H	-7.07001	0.46427	1.82031
H	-8.99228	0.12772	0.28155
H	-8.6119	-0.35383	-2.12375
H	-6.30402	-0.51277	-3.02886

H	-2.03726	-3.63032	3.46069
H	-1.48994	-4.74634	2.19714
H	0.69188	-4.23637	0.95245
H	1.79332	-1.05775	4.6459
H	4.69542	-2.32871	2.75816
H	4.17999	-1.3175	4.1201
H	4.02797	-1.4603	0.34748
H	4.32742	3.09181	2.38134
H	4.7531	4.06742	0.24208
H	5.05696	3.03453	-1.16491
H	3.15163	2.25375	-2.57775
H	0.11276	5.27354	-0.01986
H	-1.47588	5.16435	-1.94609
H	-1.28727	3.90518	-3.18087
H	-2.1113	6.33881	1.67259
H	-3.61005	5.33975	1.61634
H	-2.16532	4.75095	2.53902
H	-1.52487	-1.19019	6.21183
H	0.18561	-1.74369	6.36848
H	-0.19396	-0.20604	5.48494
H	2.6489	6.34601	2.40312
H	1.69878	6.85504	0.95519
H	1.03334	5.59746	2.08067
H	4.37043	1.91119	5.85593
H	5.37183	2.62465	4.5343
H	3.58327	2.9277	4.58468
H	-0.82775	-5.82844	-1.35123
H	-2.38587	-5.05697	-1.82559
H	-0.81146	-4.2244	-2.18415
H	0.47409	2.04752	-5.40462
H	0.4491	3.76935	-4.86067
H	2.01199	2.87484	-4.92241
H	4.48861	-4.71306	0.2954
H	4.1876	-4.80359	2.07333
H	2.94939	-5.42977	0.92267
H	4.91331	-0.04874	-3.09622
H	6.07248	0.55601	-1.85018
H	5.3435	-1.08347	-1.67341

Table S7. Computed Energies.

Energy (Hartree/Particle)	Thermal Correction to G	G
P-**H**	0.790794	-2535.16831
R-**G1**	0.196528	-519.961289
S-**G1**	0.195489	-519.958565
P-**H**·*R*-**G1**	1.010296	-3055.150322
P-**H**·*S*-**G1**	1.009938	-3055.146189

Table S8. Free Energies for *P*-**H**·*R*-**G1** and *P*-**H**·*S*-**G1** in the Gas Phase at 298K.

Complexes	ΔG (kcal mol^{-1})
P-**H**·*R*-**G1**	-13.003890
P-**H**·*S*-**G1**	-12.119728

8. CD Spectra of Chiral Hosts and the Host-Guest Complexes

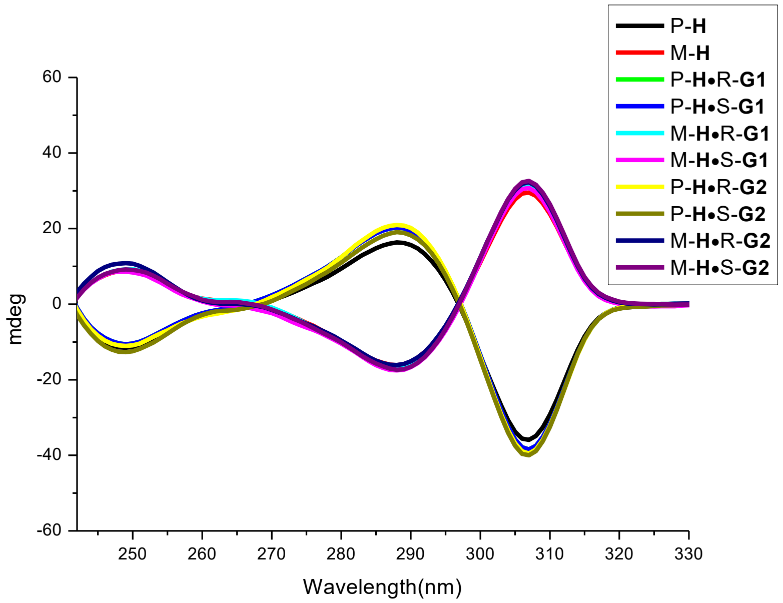

Figure S72. CD spectra of chiral hosts and the host-guest complexes (CH_2Cl_2, *c* = 5×10^{-6} M^{-1}, T = 298 K).

9. ^{1}H NMR and 2D NMR Spectra for the Complexes

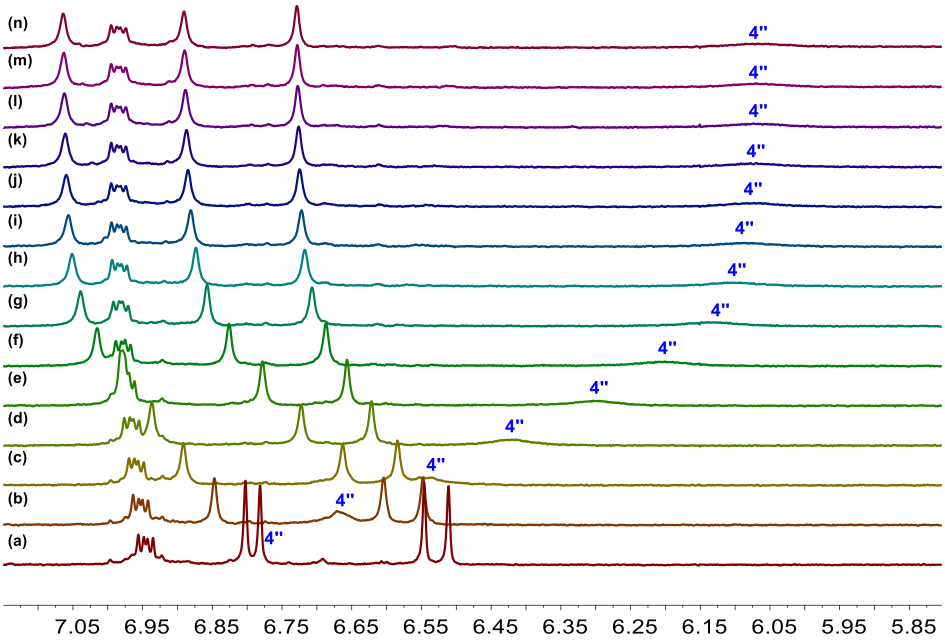

Figure S73. Partial ^{1}H NMR spectra (400 MHz, 298K, $CDCl_3$) of *M*-**H** with different equivalents of *S*-**G1**: (a) 0.00, (b) 0.20, (c) 0.40, (d) 0.60, (e) 0.80, (f) 1.00, (g) 1.20, (h) 1.40, (i)1.60, (j) 1.80, (k) 2.00, (l) 2.20, (m) 2.40, (n) 2.50. $[M\text{-}\mathbf{H}]_0$ = 3.00 mM.

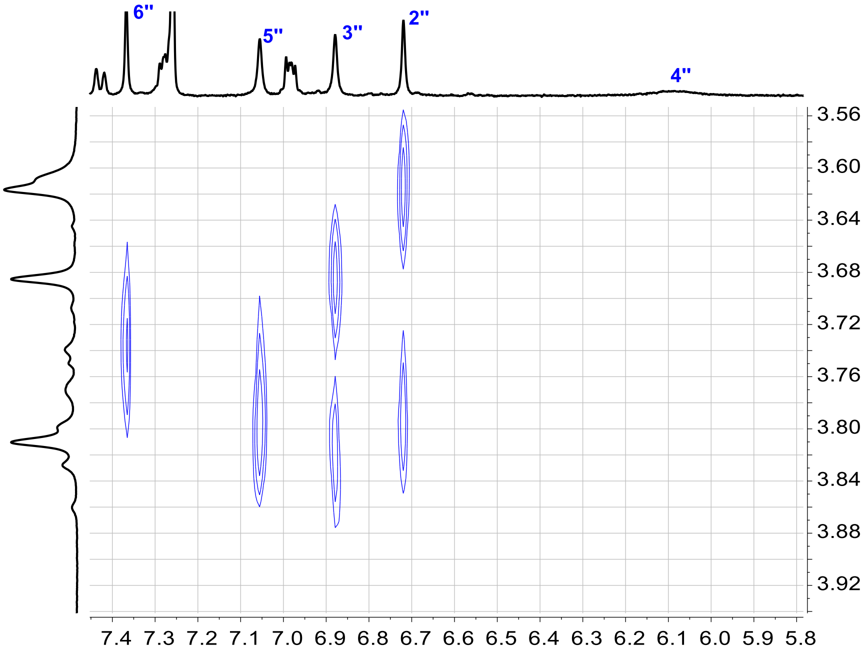

Figure S74. Partial 2D ROESY spectrum (400 MHz, 298K, $CDCl_3$) of *M*-**H**·*S*-**G1**.

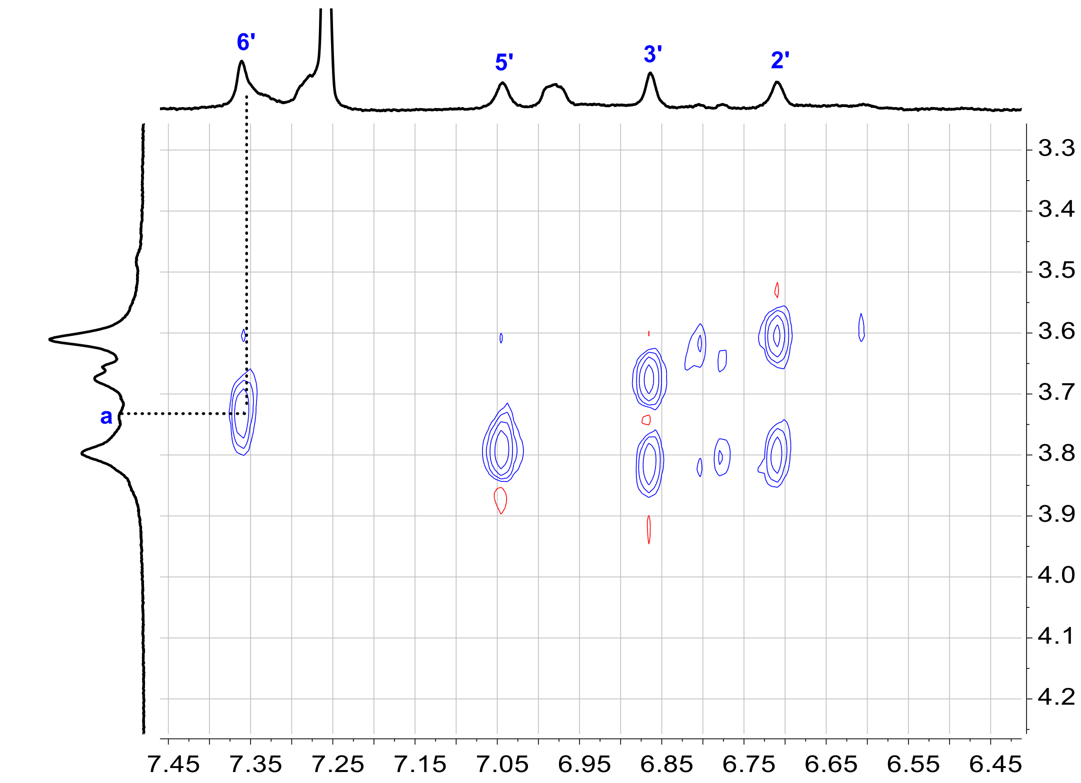

Figure S75. Partial 2D ROESY spectrum (500 MHz, 298K, $CDCl_3$) of *P*-**H**·*R*-**G1**.

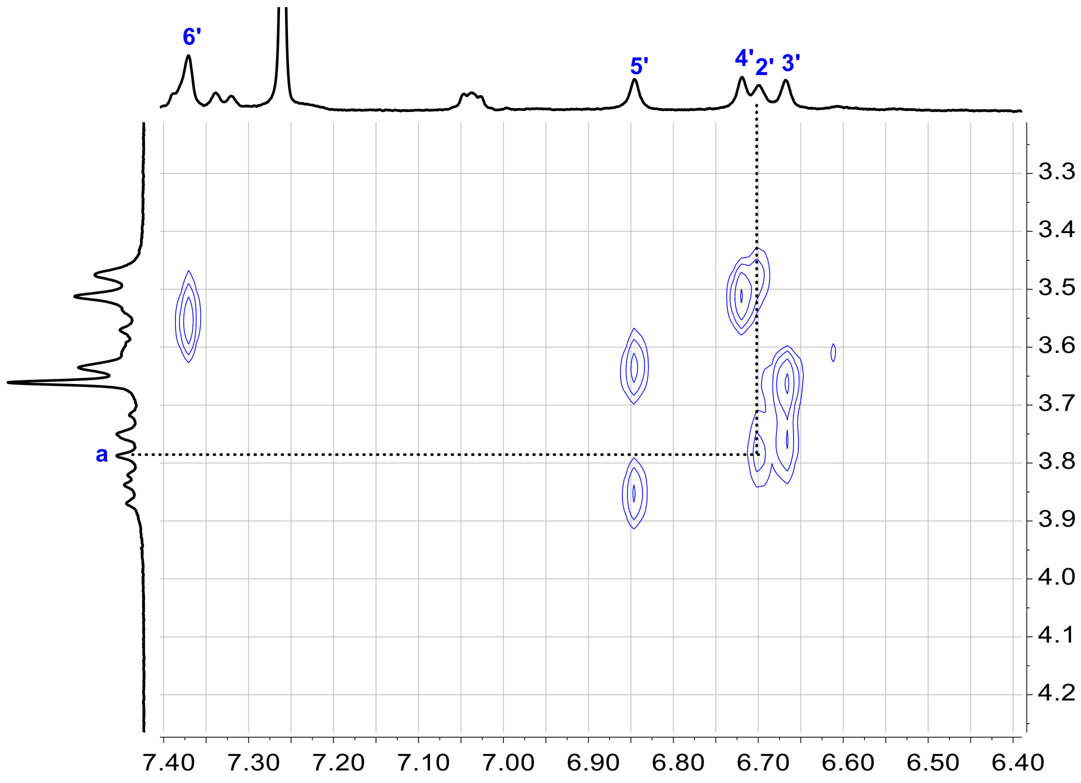

Figure S76. Partial 2D ROESY spectrum (400 MHz, 298K, $CDCl_3$) of *P*-**H**·*S*-**G1**
